# Medical Students’ Attitudes, Perceptions, and Self-Reported Familiarity With AI in Health Care: Systematic Review and Meta-Analysis

**DOI:** 10.2196/89411

**Published:** 2026-08-18

**Authors:** Wei Zhang, Jiaxue Han, Xin Han, Hang Xu, Langkun Wang, Tianhai Lin, Ping Tan, Peng Zhang, Xiaonan Zheng

**Affiliations:** 1 Department of Urology, West China Hospital, Sichuan University, No. 37 Guoxue Alley, Wuhou District, Chengdu, Sichuan Province, 610041, China, 86 18328582843; 2 Mental Health Center, West China Hospital, Sichuan University, Chengdu, Sichuan Province, China

**Keywords:** artificial intelligence, medical students, attitudes, perceptions, AI literacy, meta-analysis, systematic review, medical education

## Abstract

**Background:**

AI is increasingly encountered in clinical care and medical education, but medical students’ attitudes, perceptions, and self-reported familiarity have been assessed using heterogeneous survey instruments, AI referents, and response scales. Prior reviews often combined mixed health profession populations or summarized central estimates without fully showing variation across settings.

**Objective:**

This study aimed to synthesize quantitative evidence on medical students’ AI-related attitudes, perceptions, and self-reported familiarity while examining construct harmonization, participant independence, heterogeneity, prediction intervals, risk of bias, and certainty of evidence.

**Methods:**

We searched PubMed (MEDLINE), Embase, Web of Science, Scopus, PsycINFO, and Cochrane CENTRAL from inception to April 1, 2026; supplementary searches are described in the appendices. Eligible studies enrolled students in MD, MBBS, MBChB, or DO-equivalent medical programs, or reported separable medical student data from mixed samples. Proportion outcomes were harmonized into 9 domains and synthesized using random-effects meta-analysis with Freeman-Tukey double-arcsine transformation, Hartung-Knapp-Sidik-Jonkman–adjusted CIs, and prediction intervals. Subgroup analyses and meta-regressions were exploratory because of multiple testing, ecological confounding, and construct heterogeneity. Risk of bias and certainty were assessed using the Joanna Briggs Institute analytical cross-sectional checklist and the GRADE (Grading of Recommendations, Assessment, Development and Evaluation) framework, respectively.

**Results:**

Ninety-six cross-sectional studies from 37 countries (>45,000 medical students) were included. Summary estimates suggested favorable attitudes but wide between-setting dispersion. Positive attitude toward AI was 76.9% (95% CI 72.2%‐81.4%; prediction interval 42.2%‐98.3%; *I*²=98.3%; 44 studies; N=20,806), perceived career benefit was 78.4% (95% CI 69.5%‐86.2%; prediction interval 45.3%‐98.3%; *I*²=98.0%; 16 studies; N=9799), and support for curricular integration was 76.6% (95% CI 71.8%‐81.1%; prediction interval 47.8%‐96.1%; *I*²=97.2%; 38 studies; N=16,308). Concern about physician replacement was 39.9% (95% CI 33.6%‐46.5%; prediction interval 6.6%‐80.1%; *I*²=98.8%; 32 studies; N=16,642), willingness to learn about or adopt AI was 71.5% (95% CI 64.8%‐77.8%; prediction interval 37.9%‐95.4%; *I*²=97.7%; 22 studies; N=9199), and ethical concerns were endorsed by 62.8% (95% CI 53.9%‐71.3%; prediction interval 21.9%‐95.0%; *I*²=98.8%; 28 studies; N=14,571). Self-reported familiarity or knowledge was 63.3% (95% CI 55.9%‐70.3%; prediction interval 7.8%‐100.0%; *I*²=99.5%; 52 studies; N=27,817), and trust in AI-assisted decisions was 50.6% (95% CI 28.5%‐72.6%; prediction interval 7.3%‐93.3%; *I*²=98.1%; 8 studies; N=3007). All domains had very low certainty because of cross-sectional self-report designs, frequent use of nonvalidated or adapted instruments, wide prediction intervals, and small study effects in several domains.

**Conclusions:**

Medical students’ AI-related attitudes and curricular interest appear broadly favorable, but these estimates should not be interpreted as stable global prevalences. This review adds value by restricting the population to medical students, transparently harmonizing nonequivalent constructs, auditing mixed populations and participant independence, and reporting prediction intervals and certainty. Given very low certainty, the findings support locally adapted, exploratory AI-literacy planning and standardized measurement in future studies rather than strong claims about curriculum effectiveness.

## Introduction

AI is increasingly encountered across clinical care, including image interpretation, predictive risk modeling, and clinical decision support [1,2]. Generative AI has also become salient in medical education, where the performance of large language models (LLMs) on medical examinations has prompted consideration of AI-assisted learning [3]. The educational relevance of AI extends beyond teaching how algorithms are built: medical education must prepare learners to understand capabilities and limitations, critically appraise outputs, and preserve professional judgment and oversight when AI-enabled tools are used [4-6]. As AI systems are increasingly incorporated into information-gathering and decision support workflows, future clinicians will need to judge when an AI output is useful, when its limitations matter, and when human review must contextualize or override the output [1,2,6]. These competencies matter before graduation because medical students are preparing for environments in which AI-mediated information may increasingly shape how evidence is accessed, interpreted, and applied [1,2,6].

Understanding how medical students perceive AI is important for needs assessment and curriculum planning [7]. Learner acceptance and perceived usefulness can shape willingness to engage with a technology, as described in the Technology Acceptance Model [8] and the Unified Theory of Acceptance and Use of Technology [9]. Yet, perception-based measures are not objective performance assessments [7]. Favorable attitudes and perceived usefulness may support engagement with AI-enabled tools [8,9], whereas concerns about professional displacement may temper medical students’ attitudes toward specific AI applications [10]. Likewise, self-reported familiarity is a perception-based measure and does not by itself establish applied critical appraisal or preserved independent reasoning [7,11]. Attitude and familiarity data should therefore be treated as signals for educational needs assessment rather than as proxies for AI literacy [7,11]. The rapid diffusion of generative AI systems such as ChatGPT further sharpens this distinction because uncritical use may encourage cognitive dependence and weaken independent clinical reasoning [11].

Several prior reviews have examined AI in health professions education but not specifically among medical students enrolled in medical education programs (MD, MBBS, MBChB, or DO) [12-14]. Mousavi Baigi et al [12] reviewed health care students’ attitudes, knowledge, and skills related to AI without meta-analytic pooling. Shishehgar et al [13] synthesized the knowledge, perceptions, and experiences of health students and academics regarding AI in health education and practice. Amiri et al [14] conducted a systematic review and meta-analysis of medical, dental, and nursing students’ attitudes and knowledge toward AI. Although these reviews provide useful background, their inclusion of mixed health professions populations limits the specificity of inference for medical students [12-14]. In addition, the quantitative synthesis by Amiri et al did not apply the Hartung-Knapp-Sidik-Jonkman (HKSJ) correction recommended for random-effects CIs [15] or report prediction intervals, which are important for showing the likely range of effects across settings rather than only the pooled average [16].

Several limitations of the existing literature make a medical-student-specific synthesis necessary [12-14,16]. Prior reviews have synthesized populations extending beyond medical students to other health professions students and, in some cases, academics, and have grouped outcomes under broad review-level categories such as attitudes, knowledge, skills, perceptions, and experiences [12-14]. Measurement approaches and AI referents also vary across the literature: a validated readiness instrument such as Medical AI Readiness Scale for Medical Students (MAIRS-MS) [7] coexists with application-specific medical student perception surveys focused on radiology AI [10] and a rapidly changing generative AI context [11]. These differences in population, construct definition, and AI referent can blur conceptually distinct outcomes and make it difficult to determine whether between-study variation reflects who was surveyed, what was asked, or which technology was being considered [7,10-14,16]. In addition, narrative summaries or conventional random-effects CIs alone do not show the range of true values that may be expected across comparable settings [16]. Transparent outcome harmonization, explicit handling of mixed populations, and prediction interval–based interpretation are therefore important when pooled proportions are used to inform medical education decisions [12-14,16].

The present review was therefore designed to synthesize quantitative evidence on medical students’ AI-related attitudes, perceptions, self-reported familiarity, prior use, ethical concerns, willingness to learn, and trust; document how heterogeneous survey outcomes were harmonized into operational domains; report CIs and prediction intervals for summary estimates; assess risk of bias and certainty of evidence; examine participant independence and mixed population disaggregation; evaluate subgroup patterns and feasibility-screened meta-regression; and explore the implications of these findings for AI literacy curriculum development.

## Methods

### Ethical Considerations

This review synthesized data from previously published studies only and involved no direct participant contact, no collection of identifiable personal data, and no experimental intervention. Under the policies of the institutional review board of West China Hospital, Sichuan University, systematic reviews are exempt from ethical review. Informed consent was therefore not applicable.

### Study Registration and Reporting

The protocol was prospectively registered in PROSPERO (CRD420251120543). The review was conducted with reference to the Cochrane Handbook for Systematic Reviews of Interventions [17] and reported in accordance with PRISMA (Preferred Reporting Items for Systematic Reviews and Meta-Analyses) 2020 [18], PRISMA 2020 for Abstracts, and the PRISMA-S (Preferred Reporting Items for Systematic Reviews and Meta-Analyses literature search extension) reporting [19]. Completed reporting checklists are provided in Checklist 1.

### Eligibility Criteria

Eligible participants were students enrolled in medical education programs (MD, MBBS, MBChB, or DO-equivalent programs), aligned with World Federation for Medical Education standards [20], from entry into training through internship. Studies with mixed populations were included only when medical student data were reported separately or could be extracted without including nonmedical participants. Mixed population records were reviewed in dedicated supplementary tables; any study without separable medical student data was excluded.

### Information Sources and Search Strategy

We searched PubMed (MEDLINE), Embase, Web of Science, Scopus, PsycINFO, and Cochrane CENTRAL from inception to April 1, 2026. Search strategies used 3 concept blocks: medical student population terms, AI-related exposure terms, and survey terms related to attitudes, perceptions, and familiarity. For each database, the search platform, date of search, coverage, complete line-by-line query, limits, and number of records retrieved were documented in Multimedia Appendix 1 in accordance with PRISMA-S [19]. Supplementary searching included Google Scholar screening of the first 200 relevance-sorted records and backward and forward citation searching of included studies and prior reviews. Where the search records available to the review team did not contain a raw export, time-stamped log, or other procedural detail for a supplementary search (such as the exact search date, query string, sort order, or screener identity), that detail is marked as not recorded rather than reconstructed; checklist items that did not apply to the search methods are marked as not applicable in the completed PRISMA-S checklist (Checklist 1).

### Selection Process and Data Collection

Records were imported into EndNote 21 (Clarivate) and deduplicated using automated matching by DOI, title, author, year, and journal, followed by manual verification [21] (Table S1 in Multimedia Appendix 2). The PRISMA 2020 study selection flow diagram is shown in the Results section, and reports not retrieved, eligibility-exclusion reasons, and the exclusion-impact comparison are documented in Tables S2 and S3 in Multimedia Appendix 2. During eligibility adjudication, mixed population screening records and full separability decisions were documented in Tables S4 and S5 in Multimedia Appendix 2. Two reviewers (WZ and JH) independently screened titles and abstracts, then full texts, with disagreements resolved by a third reviewer (XZ). Two reviewers independently extracted data using a piloted standardized form. Extracted variables included bibliographic details, study setting, population characteristics, instrument type and validation status, type of AI evaluated, and outcome data for 9 proportion domains, 5 MAIRS-MS outcomes (the total score and 4 subscales), and other noncomparable continuous measures; study-level characteristics are summarized in Table S6 in Multimedia Appendix 2.

### Data Harmonization

Before synthesis, extracted survey items were mapped to the 9 proportion domains using an a priori operational framework. For every study-domain contribution, the harmonization record captured the original item wording or closest reported paraphrase, response scale, scale points, dichotomization rule, neutral-response handling, event count, denominator, and rationale for domain assignment. When papers enrolled mixed populations, only medical student–specific data were retained; if medical student event counts or denominators could not be separated from nonmedical groups, that domain was coded as nonextractable. These decisions are reported in the mixed population documentation and outcome harmonization tables.

### Outcome Domains

Nine proportion domains were prespecified on the basis of the Technology Acceptance Model [8], the Theory of Planned Behavior [22], and the AI Literacy framework [23]: (P1) positive attitude toward AI, (P2) AI perceived as beneficial to medical career, (P3) support for AI curricular integration, (P4) concern about physician replacement, (P5) self-reported familiarity or knowledge, (P6) prior AI tool use, (P7) willingness to learn about or adopt AI, (P8) ethical concerns, and (P9) trust in AI-assisted decisions. These domains were treated as operational approximations rather than standardized constructs. For Likert-type items, endorsement categories such as agree or strongly agree, yes, aware, familiar, or equivalent positive responses were extracted as events when reported; neutral responses remained in the denominator unless a source study explicitly excluded them. The full outcome harmonization table and harmonization verification table report item wording, denominator, cutoff notes, assigned domain, and assignment rationale.

### Risk-of-Bias Assessment

Two reviewers independently assessed risk of bias using the Joanna Briggs Institute Critical Appraisal Checklist for Analytical Cross-Sectional Studies [24]. This tool was selected because the included surveys were analytical cross-sectional studies that frequently combined descriptive estimation of attitudes or familiarity with comparisons by training stage, exposure, region, or other participant characteristics. Although the review synthesized proportional self-report outcomes rather than causal associations, the Joanna Briggs Institute (JBI) domains remained relevant to sampling, participant description, exposure and outcome measurement, confounding, and statistical reporting. We also considered the JBI Critical Appraisal Checklist for Prevalence Studies; because most included surveys reported analytical comparisons across training stage, exposure, or region in addition to descriptive proportions, the analytical cross-sectional checklist was judged to capture the relevant domains, including confounding and exposure and outcome measurement, more completely and was applied to all studies for consistency. Studies scoring 6‐8 were classified as low risk of bias, 4‐5 as moderate risk, and 0‐3 as high risk. No study met the high-risk scoring threshold, but common limitations including convenience sampling, nonvalidated instruments, unclear response-rate denominators, and self-selection were incorporated into the certainty assessment and interpretation. The item-level JBI checklist is provided in Table S7 in Multimedia Appendix 2.

### Synthesis Methods

For proportion outcomes, study-specific estimates were pooled after Freeman-Tukey double-arcsine transformation [25,26] using random-effects models [27] with HKSJ–adjusted CIs [15]. Random-effects models were prespecified for all meta-analyses because true effect sizes were expected to vary across populations, settings, instruments, and item wording [28]. Prediction intervals were calculated and reported for the primary proportional meta-analyses whenever 3 or more studies contributed [16]. For these analyses, CIs describe uncertainty around the pooled summary estimate, whereas prediction intervals describe the range of true proportions that may be expected in a new comparable setting. The width of the prediction intervals from the primary proportional meta-analyses was treated as the empirical rationale for proceeding with the prespecified subgroup analyses and feasibility-screened meta-regressions, which explored potential sources of between-setting dispersion. All analyses were performed with R (version 4.5.3; R Foundation for Statistical Computing) using the meta (version 8.5-0) and metafor (version 5.0-1) packages, both obtained from the Comprehensive R Archive Network.

Certainty of evidence was assessed for each proportional outcome using the GRADE (Grading of Recommendations, Assessment, Development and Evaluation) framework for bodies of observational evidence and absolute event-rate estimates [29-31]. The pooled proportion was the absolute estimate of interest. Risk of bias considered JBI ratings and recurring limitations in sampling, response-rate reporting, and instrument validation. Prediction intervals were used as the primary basis for interpreting the practical magnitude of between-setting heterogeneity because they describe the range of true proportions that may be expected in a new comparable setting [16]. *I*² and τ² were reported for completeness but were not used to label heterogeneity as low, moderate, high, or extreme. Imprecision considered CI width, prediction interval width, and the number of contributing studies and participants. Indirectness considered whether survey items, AI technologies, populations, and educational contexts matched the target construct, and publication bias considered funnel plot asymmetry and small study effect tests where feasible. Because all evidence came from cross-sectional self-report surveys and prediction intervals were very wide across the proportional domains, certainty ratings were interpreted conservatively.

For continuous outcomes reported on the same scale, pooled means were synthesized using random-effects meta-analysis with the same HKSJ framework. These analyses were treated as secondary descriptive syntheses; prediction intervals were not reported or interpreted for the continuous outcomes. Continuous outcomes reported on noncomparable scales were summarized descriptively.

Prespecified subgroup analyses examined publication period, World Bank income level, World Health Organization (WHO) region, type of AI evaluated, and risk of bias. AI type was assigned using a deterministic hierarchy: studies mentioning ChatGPT, LLMs, chatbots, or generative AI were classified as ChatGPT or LLM or generative AI; otherwise, studies focused on radiology, ophthalmology, specialty- or domain-specific AI, or clinical decision tools were classified as domain-specific AI. All remaining studies were classified as broad or general AI. For studies mentioning more than 1 AI type, this hierarchy supplied 1 study-level assignment and studies were not split or weighted across AI-type categories. World Bank income level and WHO region were assigned according to the country or countries in which the study population was surveyed. Single-country studies used that country’s region and income band; multicountry studies were coded dimension-wise, with studies spanning multiple WHO regions classified as Multiple regions or Other, and studies spanning multiple-income bands or global or unspecified country mixes classified as mixed income, without weighting by participant distribution. Where *k*≥10, univariable meta-regression was performed using mixed-effects models to examine whether each moderator, considered separately, was associated with between-study variation. Because multiple subgroup analyses can inflate type I error and study-level moderators are vulnerable to ecological confounding, we added feasibility-screened meta-regression and bubble plots only when *k* was at least 10, at least 2 moderator levels were present, and no level contained fewer than 3 studies. All subgroup and meta-regression findings were interpreted as exploratory and hypothesis-generating.

### Participant Independence Verification

To assess potential participant duplication, we conducted a 4-dimensional participant independence assessment across all included studies, examining author and ethics approval concordance, overlap between multinational and local surveys, spatiotemporal institutional concordance, and survey platform congruence (Table S8 in Multimedia Appendix 2). Studies with possible overlap were retained in the primary analysis only when duplication could not be confirmed and were prespecified for sensitivity analysis. The finalized proportion and continuous extraction datasets are provided in Tables S9 and S10 in Multimedia Appendix 2, and the outcome harmonization and source verification records are provided in Tables S11 and S12 in Multimedia Appendix 2.

## Results

### Study Selection

**Figure 1.**
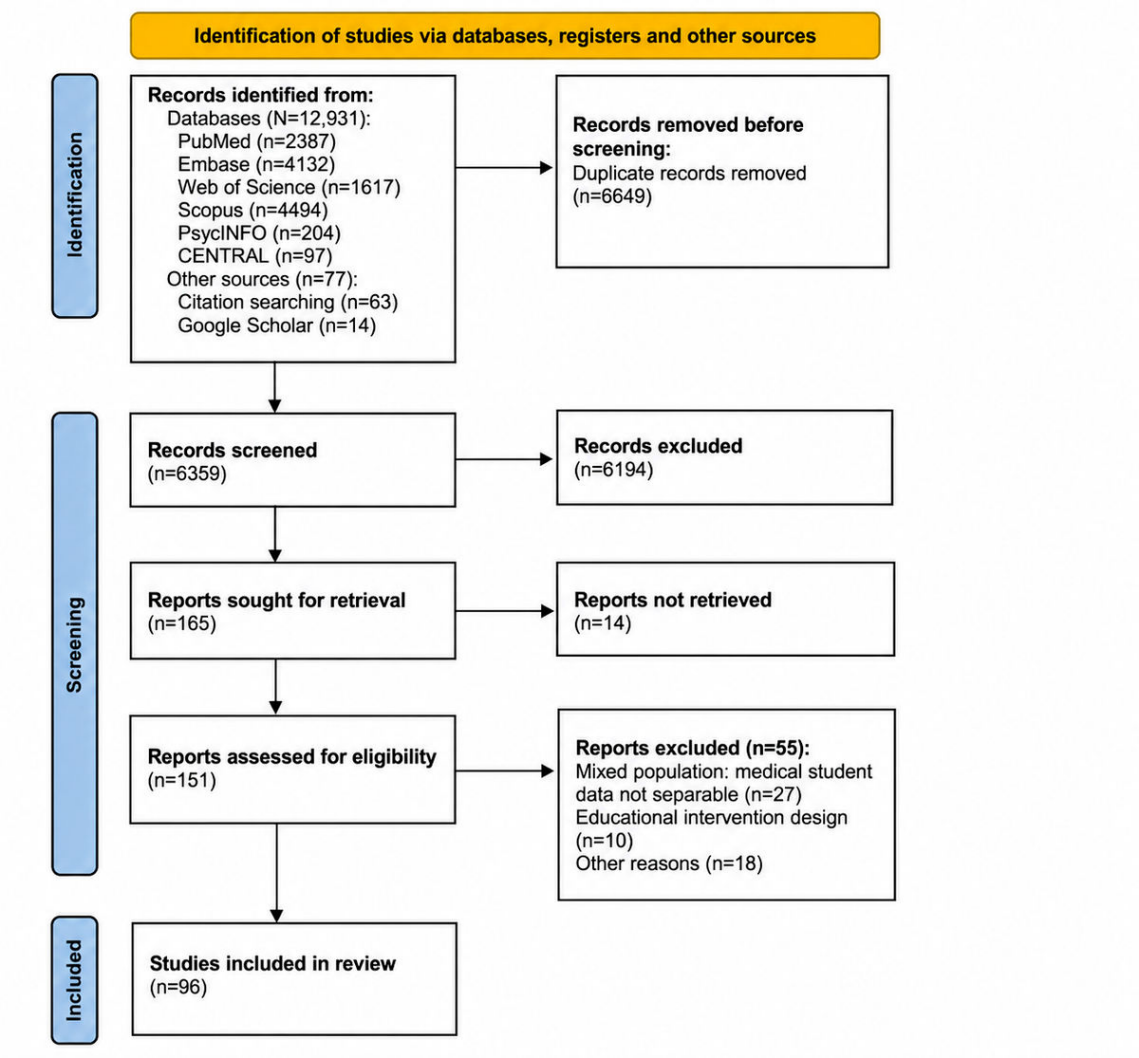
PRISMA (Preferred Reporting Items for Systematic Reviews and Meta-Analyses) 2020 flow diagram of study identification, screening, and inclusion, drawn with the official PRISMA 2020 template. Records identified through supplementary citation searching and Google Scholar were exported to the reference manager and deduplicated together with database records; all records therefore flow through a single screening pathway.

Database searching yielded 12,931 records and supplementary searching contributed 77 more, for a total of 13,008. After removing 6649 duplicates, 6359 unique records were screened at the title and abstract level; 6194 were excluded. Of 165 reports sought for retrieval, 14 could not be retrieved and 151 were assessed at full text; 55 of these were excluded, most often because they enrolled mixed populations from which medical student data could not be separated (n=27) or used an educational intervention design (n=10), with 18 excluded for other prespecified reasons (Figure 1). Two of these exclusions (Sami et al [32]; Farooq and Usmani [33]) were studies that initially appeared eligible but were removed after detailed examination because their reported proportions combined medical with dental and allied health students and medical student–specific counts could not be recovered. A total of 96 studies were included [10,34-128].

### Study Characteristics

The 96 cross-sectional surveys were published between 2019 and 2026; 82 (85%) appeared from 2023 onward (Table 1). They originated from 37 countries spanning 6 WHO regions, led by the Eastern Mediterranean (38/96, 40%), Europe (19/96, 20%), and the Western Pacific (12/96, 13%). Forty-three studies (45%) came from high-income countries, 28 (29%) from low- or lower-middle-income countries, 20 (21%) from upper-middle-income countries, and 5 (5%) were multicountry mixed-income studies. Sample sizes ranged from 20 to 4492 medical students. Fifty-nine studies (62%) assessed attitudes toward broad or general AI, 27 (28%) focused on ChatGPT or other LLMs, and 10 (10%) evaluated domain-specific applications such as radiology AI. Thirteen studies used the validated MAIRS-MS [7]; the remainder relied on adapted or self-developed questionnaires.

**Table 1.** Characteristics of 96 included studies (2019‐2026; 37 countries; >45,000 medical students).

Characteristic and category		Studies, n (k)	Total percentage
Publication period			
2023 onward		82	85.4
Pre-2023		14	14.6
WHO^a^ region			
Eastern Mediterranean		38	39.6
Europe		19	19.8
Western Pacific		12	12.5
South-East Asia		11	11.5
Americas		10	10.4
Other^b^		6	6.2
World Bank income			
High income		43	44.8
Low- or lower-middle income		28	29.2
Upper-middle income		20	20.8
Mixed income		5	5.2
Data type			
Proportion only		72	75.0
Continuous only		13	13.5
Both		11	11.5
Sample size			
<100		5	5.2
100‐499		63	65.6
500‐999		18	18.8
≥1000		10	10.4
JBI^c^ risk of bias			
Low		17	17.7
Moderate		79	82.3
AI type evaluated			
Broad or general AI		59	61.5
ChatGPT or LLM^d^		27	28.1
Domain-specific AI		10	10.4

a WHO: World Health Organization.

b Includes Africa (n=1) and multinational (n=5).

c JBI: Joanna Briggs Institute.

d LLM: large language model.

### Risk of Bias and Participant Independence

On the JBI checklist, 18% (17/96) of the studies were rated low risk and 82% (79/96) were rated moderate risk; none scored in the high-risk range (Table 2). The absence of high-risk classifications reflects the prespecified scoring threshold rather than absence of methodological limitations. The most common shortcomings were inadequate identification and management of confounding, convenience or voluntary sampling, self-developed questionnaires, unclear response rate denominators, and self-report outcomes; these limitations informed the very low certainty ratings. The participant independence assessment identified 8 clusters requiring detailed review. Four were cleared after source-level examination. In each of the remaining 4 clusters, only 1 eligible report contributed data to the meta-analysis; therefore, no pair of included studies had confirmed participant overlap. No included study was removed for duplication, and the retained studies were covered by the prespecified leave-one-out sensitivity analyses.

**Table 2.** Methodological quality (risk of bias) of the 96 included studies, appraised with the Joanna Briggs Institute critical appraisal checklist for analytical cross-sectional studies^a^.

JBI^b^ appraisal item	Yes, n (%)	Unclear, n (%)	No, n (%)
Were the criteria for inclusion in the sample clearly defined?	96 (100)	0 (0)	0 (0)
Were the study subjects and the setting described in detail?	96 (100)	0 (0)	0 (0)
Was the exposure measured in a valid and reliable way?	78 (81)	18 (19)	0 (0)
Were objective, standard criteria used for measurement of the condition?	0 (0)	0 (0)	96 (100)
Were confounding factors identified?	17 (18)	0 (0)	79 (82)
Were strategies to deal with confounding factors stated?	15 (16)	0 (0)	81 (84)
Were the outcomes measured in a valid and reliable way?	96 (100)	0 (0)	0 (0)
Was appropriate statistical analysis used?	96 (100)	0 (0)	0 (0)

a Each item was scored Yes=1 and No or Unclear=0 (maximum 8). Overall methodological quality was summarized with review-defined descriptive thresholds (6-8=low risk of bias, 4-5=moderate, and 0-3=high); these thresholds are descriptive and were not used to exclude studies. Across the 96 studies, 17 (18%) were low risk, and 79 (82%) were moderate risk, and none were high risk; total scores ranged from 4 to 7 (score 4: n=16; 5: n=63; 6: n=4; and 7: n=13).

b JBI: Joanna Briggs Institute.

### Proportion Outcomes

**Figure 2.**
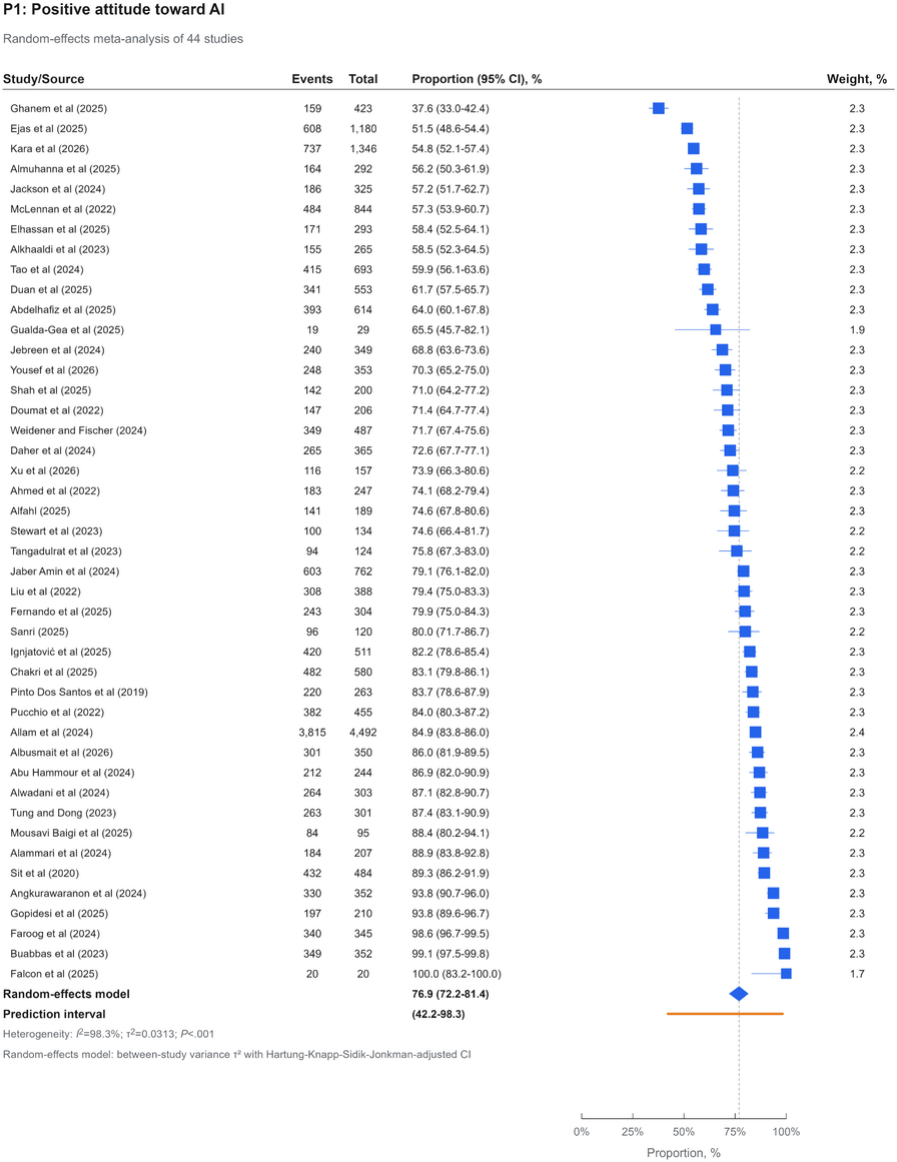
Forest plot of positive attitude toward AI (domain P1) across 44 studies (N=20,806). Blue squares indicate study-specific proportions, with square size proportional to the study’s random-effects weight; horizontal blue lines indicate 95% CIs. The blue diamond indicates the pooled random-effects estimate and its 95% CI, and the orange horizontal line indicates the 95% prediction interval [10,35,38,39,41,44,46,50,55,57,58,60,62-67,69-71,81,82,84,86-88,90-92,94,95,100,107,108,110,111,113,116-118,121,122,128].

**Figure 3.**
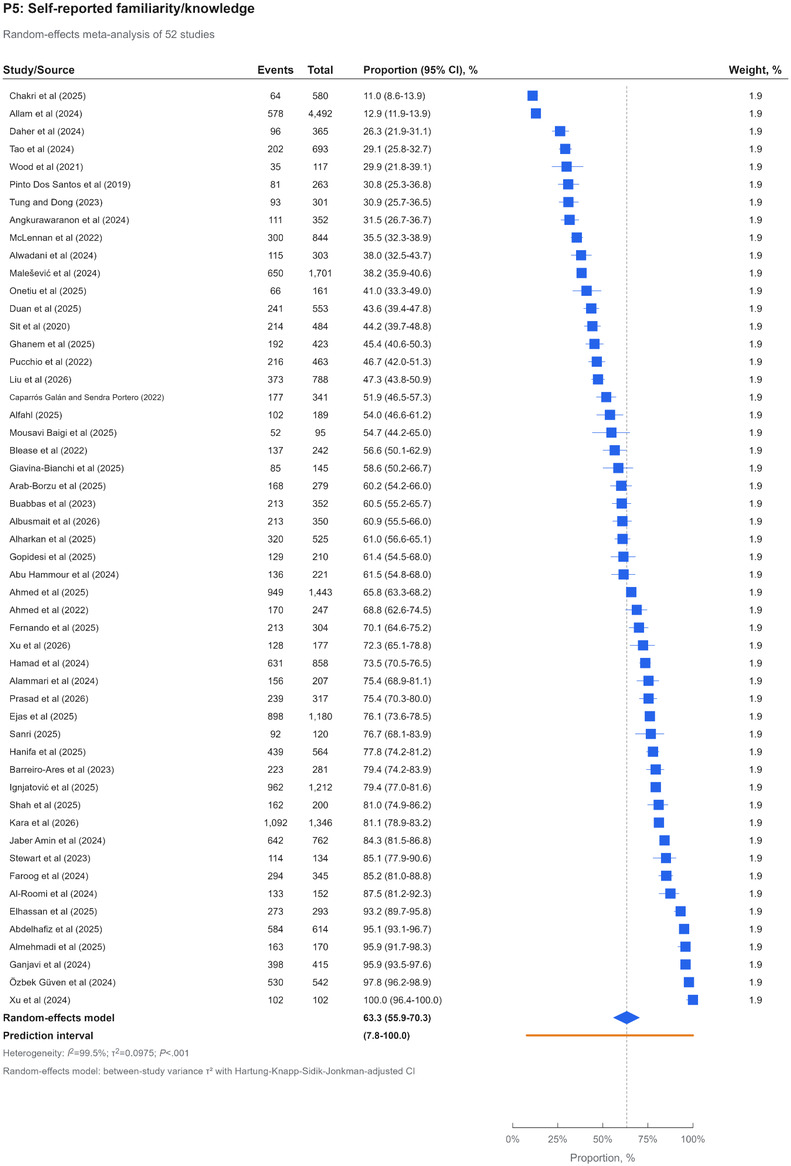
Forest plot of self-reported familiarity with or knowledge of AI (domain P5) across 52 studies (N=27,817). Blue squares indicate study-specific proportions, with square size proportional to the study’s random-effects weight; horizontal blue lines indicate 95% CIs. The blue diamond indicates the pooled random-effects estimate and its 95% CI, and the orange horizontal line indicates the 95% prediction interval [10,35,38-40,44-47,50,52-55,58-62,64-67,69,70,76,81,84-88,90,92,94,98-100,102,103,105,110,111,113,114,117,119,121-123,127,128].

**Figure 4.**
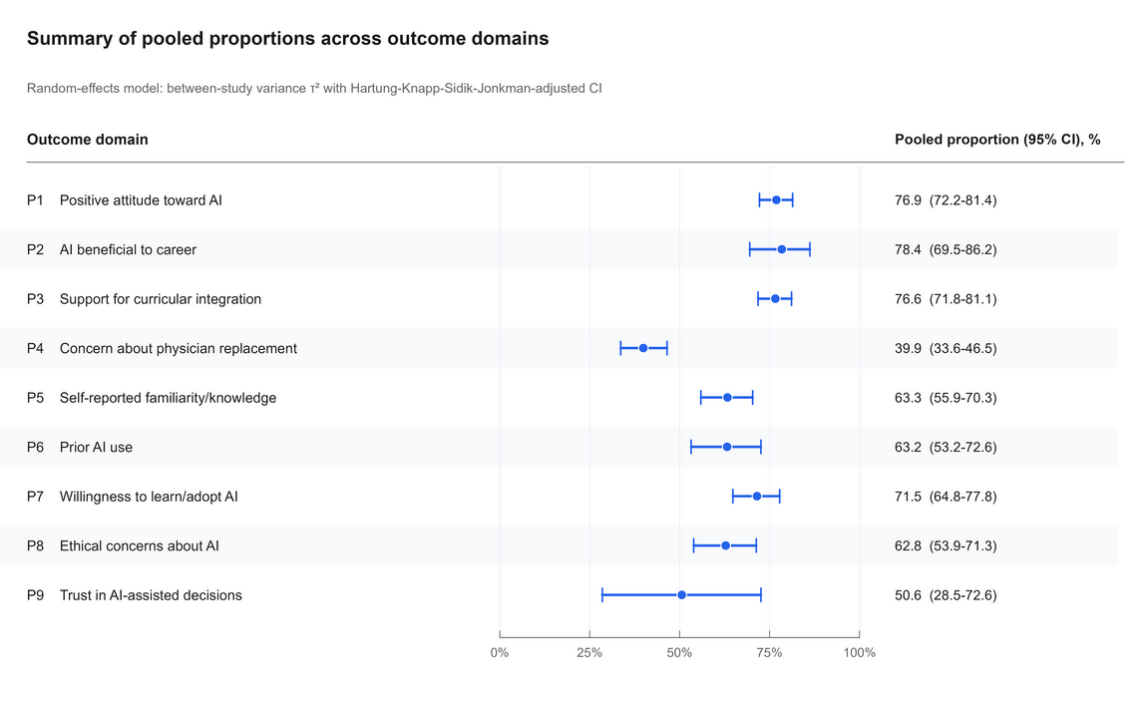
Cross-domain graphical summary of the random-effects pooled proportions and 95% CIs for the 9 outcome domains (P1-P9).

Table 3 provides the complete numerical synthesis for all 9 proportional domains, including pooled estimates, prediction intervals, and complementary model statistics. Positive attitude toward AI was endorsed by a pooled 76.9% (Figure 2; Table 3), and self-reported familiarity by 63.3% (Figure 3). Each primary proportional meta-analysis is accompanied by a domain-level forest plot in which the 95% prediction interval is printed beneath the pooled estimate (Figures 2 and 3; Figures S1-S7 in Multimedia Appendix 2). Figure 4 provides a complementary cross-domain visual overview of pooled proportions and 95% CIs only. Certainty of evidence is summarized later in the dedicated GRADE Summary of Findings. These estimates should be read as average summaries of heterogeneous self-report items rather than stable global prevalences. The 95% prediction intervals were wide across all domains and, in several domains, extended from low endorsement to near-universal endorsement, indicating that the true proportion expected in a new comparable setting could differ markedly from the pooled mean [16]. *I*² and τ² are reported in Table 3 for completeness; the practical magnitude of between-setting heterogeneity is interpreted from the prediction intervals and not from the *I*² values [16]. Because each domain draws on a different set of studies, instruments, denominators, and item wordings, comparisons across domains (eg, attitude vs trust, or familiarity vs curricular support) are indirect and should be read as descriptive and exploratory rather than as a ranking of constructs.

**Table 3.** Primary proportional meta-analyses, 95% prediction intervals, and model statistics (Freeman-Tukey double-arcsine transformation; Hartung-Knapp-Sidik-Jonkman [HKSJ]–adjusted CIs).

Outcome domain	*k*	N	Pooled proportion, % (95% CI)	95% PI^a^, %	*I*², %	τ²
P1: positive attitude	44	20,806	76.9 (72.2‐81.4)	42.2‐98.3	98.3	0.031
P2: career benefit	16	9799	78.4 (69.5‐86.2)	45.3‐98.3	98.0	0.025
P3: curricular integration	38	16,308	76.6 (71.8‐81.1)	47.8‐96.1	97.2	0.021
P4: replacement concern	32	16,642	39.9 (33.6‐46.5)	6.6‐80.1	98.8	0.042
P5: familiarity or knowledge	52	27,817	63.3 (55.9‐70.3)	7.8‐100.0	99.5	0.097
P6: prior AI use	36	17,374	63.2 (53.2‐72.6)	8.5‐100.0	99.4	0.091
P7: willingness to learn or adopt	22	9199	71.5 (64.8‐77.8)	37.9‐95.4	97.7	0.026
P8: ethical concerns	28	14,571	62.8 (53.9‐71.3)	21.9‐95.0	98.8	0.042
P9: trust in AI-assisted decisions^b^	8	3007	50.6 (28.5‐72.6)	7.3‐93.3	98.1	0.041

a PI: prediction interval.

b *k*<10; interpret with caution.

### Subgroup Analyses

Because the primary meta-analyses yielded very wide prediction intervals (Table 3), indicating substantial expected between-setting dispersion, the prespecified subgroup analyses and meta-regressions were undertaken to explore potential sources of this variation. Selected prespecified subgroup analyses are summarized for 4 main domains across 4 moderators (Table 4; Figures S8-S27 in Multimedia Appendix 2). Four statistically significant subgroup interactions emerged among the displayed domains. Self-reported familiarity was higher in studies published from 2023 onward (66.4%) than before 2023 (45.6%; *P*=.04). AI type was associated with positive attitude (broad or general AI, 80.1%; ChatGPT or LLM, 66.7%; domain-specific AI, 83.2%; *P*=.005), replacement concern (45.9%, 43.4%, and 27.2%, respectively; *P*=.01), and familiarity (60.9%, 77.4%, and 45.1%, respectively; *P*=.008). Prior AI use was 64.9% in studies published from 2023 onward, while the pre-2023 level was not pooled because only 1 study contributed. No statistically significant differences were detected between low-risk and moderate-risk studies in any displayed domain; these null comparisons should not be interpreted as evidence of equivalence because subgroup power was limited.

**Table 4.** Prespecified subgroup analyses for selected proportion domains by publication period, World Bank income, AI type, and risk of bias.

Subgroup	P1, % (95% CI)	P4, % (95% CI)	P5, % (95% CI)	P6, % (95% CI)
Publication period				
2023 onward	76.8 (71.2‐82.0)	41.9 (34.9‐49.2)	66.4 (58.3‐74.1)	64.9 (55.4‐73.9)
Pre-2023	77.7 (67.4‐86.5)	31.4 (15.2‐50.4)	45.6 (34.4‐56.9)	Not pooled (*k*=1)
*P* value for interaction	.98	.17	.04	Not run
World Bank income				
High income	79.2 (71.1‐86.2)	33.9 (24.6‐44.0)	65.9 (54.6‐76.4)	62.1 (47.2‐75.9)
Upper-middle income	77.0 (65.4‐86.8)	47.4 (36.9‐58.0)	60.9 (44.4‐76.3)	75.0 (52.4‐92.1)
Lower-middle or low income	74.9 (65.7‐83.1)	47.8 (35.8‐59.9)	63.6 (48.9‐77.1)	49.8 (29.3‐70.3)
*P* value for interaction	.69	Not run	.68	Not run
AI type				
Broad or general	80.1 (74.8‐84.9)	45.9 (36.6‐55.4)	60.9 (52.5‐69.0)	57.0 (40.0‐73.2)
ChatGPT or LLM^a^	66.7 (56.3‐76.3)	43.4 (29.7‐57.6)	77.4 (62.6‐89.4)	69.1 (57.6‐79.6)
Domain-specific	83.2 (69.8‐93.3)	27.2 (17.8‐37.7)	45.1 (23.2‐68.1)	Not run
*P* value for interaction	.005	.01	.008	.31
JBI^b^ risk of bias				
Low	74.2 (21.1‐100.0)	51.6 (16.2‐86.1)	63.4 (32.7‐89.2)	76.5 (54.1‐93.0)
Moderate	77.2 (72.7‐81.4)	38.3 (31.9‐44.9)	63.2 (55.7‐70.4)	59.1 (47.7‐70.0)
*P* value for interaction	.94	.17	.89	.10

a LLM: large language model.

b JBI: Joanna Briggs Institute.

### Meta-Regression

Univariable meta-regression was performed for 20 domain-moderator combinations (Tables S13 and S14 in Multimedia Appendix 2). These meta-regression tests assess whether a moderator explains between-study variance across the full set of contributing studies and therefore complements, rather than duplicates, the categorical subgroup comparisons reported in Table 4; the small difference in the number of significant findings reflects the different statistical questions addressed by each approach. Five models reached statistical significance: AI type was associated with between-study variation in positive attitude (P1; *P*=.005), perceived career benefit (P2; *P*=.04), replacement concern (P4; *P*=.01), and self-reported familiarity (P5; *P*=.008), while publication period was associated with variation in self-reported familiarity (P5; *P*=.04).

### Sensitivity Analyses

Across all 9 domains, sensitivity analyses produced directionally consistent estimates, although the magnitude of leave-one-out shifts varied by domain (Table 5; Table S15 in Multimedia Appendix 2). The largest maximum change was observed for trust in AI-assisted decisions (6.7 percentage points), followed by perceived career benefit and ethical concerns (2.8 percentage points each); all other domains shifted by 1.7 percentage points or less. Leave-one-out analysis confirmed that no single study changed the overall interpretation of any summary estimate (Figures S28-S36 in Multimedia Appendix 2).

**Table 5.** Sensitivity analyses and small study effects assessment for 9 proportion domains.

Domain	Primary, %	Sensitivity range, %	Maximum Δ, pp^a^	Egger *P* value
P1: positive attitude	76.9	76.1‐77.7	0.8	<.001
P2: career benefit	78.4	77.2‐81.2	2.8	.13
P3: curricular integration	76.6	75.6‐77.5	1.0	<.001
P4: replacement concern	39.9	38.7‐40.9	1.3	.46
P5: familiarity	63.3	62.1‐64.4	1.1	<.001
P6: prior AI use	63.2	61.6‐64.9	1.7	<.001
P7: willingness to adopt	71.5	70.2‐72.9	1.3	<.001
P8: ethical concerns	62.8	61.2‐65.6	2.8	.48
P9: trust in AI	50.6	44.1‐57.3	6.7	Not performed (*k*<10)

a pp: percentage points.

### Small Study Effects

Small study effect tests were performed for domains with *k*≥10 and not performed for trust in AI-assisted decisions (*k*=8). Regression tests on logit proportions [129,130] indicated asymmetry for several domains, including positive attitude, curricular integration, familiarity or knowledge, prior AI use, and willingness to learn or adopt AI (funnel plots, Figures S37-S44 in Multimedia Appendix 2; Table S16 in Multimedia Appendix 2). These results are reported as small study effects rather than proof of publication bias because funnel asymmetry in proportion meta-analyses can also arise from heterogeneity, instrument differences, sampling frames, and outcome definition.

### Continuous Outcomes and Certainty of Evidence

Continuous outcomes were synthesized from the extracted MAIRS-MS data. Twelve studies contributed to the total readiness score, and 11 studies contributed to each subscale synthesis. The contributing studies differed in setting and instrument implementation, and their study-level estimates varied across the forest plots (Table S10 and Figures S45-S49 in Multimedia Appendix 2). The pooled means therefore have limited stand-alone and cross-setting interpretability. These secondary analyses are presented descriptively and are not used as a basis for strong conclusions or curricular recommendations. Bubble plots for the statistically significant meta-regression moderators are provided in Figures S50-S54 in Multimedia Appendix 2.

Certainty of evidence was assessed with the GRADE framework applied to each pooled proportion as a single-group absolute estimate and was rated very low for all 9 domains. Table 6 presents the reader-facing Summary of Findings, restricted to the key estimate, certainty rating, and interpretation for each outcome; the full prediction intervals and complementary model statistics remain in Table 3, and the detailed GRADE evidence profile is provided in Table S17 in Multimedia Appendix 2.

**Table 6.** Grading of Recommendations, Assessment, Development and Evaluation summary of findings for medical students’ AI-related attitudes, perceptions, and self-reported familiarity (single-group proportional outcomes)^a^.

Outcome	Participants (studies), n (*k*^b^)	Pooled absolute proportion, % (95% CI)	Certainty of evidence (GRADE^c^)	What the evidence means
P1: positive attitude toward AI	20,806 (44)	76.9 (72.2‐81.4)	⊕○○○ Very low	Favorable attitudes were common but varied markedly across settings.
P2: AI perceived as beneficial to career	9799 (16)	78.4 (69.5‐86.2)	⊕○○○ Very low	Career benefit was commonly perceived, but applicability to individual schools is uncertain.
P3: support for AI curricular integration	16,308 (38)	76.6 (71.8‐81.1)	⊕○○○ Very low	Support was common; this does not establish curriculum effectiveness.
P4: concern about physician replacement	16,642 (32)	39.9 (33.6‐46.5)	⊕○○○ Very low	A substantial minority expressed concern, with marked contextual variation.
P5: self-reported familiarity or knowledge	27,817 (52)	63.3 (55.9‐70.3)	⊕○○○ Very low	Self-reported familiarity is not equivalent to objective AI literacy.
P6: prior AI use	17,374 (36)	63.2 (53.2‐72.6)	⊕○○○ Very low	Prior use does not establish competent or clinically appropriate use.
P7: willingness to learn about or adopt AI	9199 (22)	71.5 (64.8‐77.8)	⊕○○○ Very low	Willingness was common; local needs assessment remains necessary.
P8: ethical concerns about AI	14,571 (28)	62.8 (53.9‐71.3)	⊕○○○ Very low	Ethical concern may reflect appropriate awareness rather than resistance.
P9: trust in AI-assisted decisions	3007 (8)	50.6 (28.5‐72.6)	⊕○○○ Very low	Trust was highly uncertain; only 8 studies contributed.

a Population: students enrolled in doctor of medicine MD, MBBS, MBChB, or DO-equivalent medical programs. Settings: 37 countries across 6 WHO regions. Outcome assessment: harmonized self-report survey items from cross-sectional studies. This reader-facing table is structured in the GRADEpro Summary of Findings format, which presents the key outcome estimates and certainty ratings concisely without reproducing the detailed certainty-domain judgments. Because the review synthesizes noncomparative single-group proportions, comparator, relative-effect, and absolute-difference columns are not applicable. Full numerical results, including 95% prediction intervals, *I*², and τ², are reported in Table 3; the detailed domain-level GRADE evidence profile is provided in Table S17 in Multimedia Appendix 2. ⊕○○○ denotes very low certainty.

b k: number of studies.

c GRADE: Grading of Recommendations, Assessment, Development and Evaluation.

## Discussion

### Principal Findings

This systematic review and meta-analysis synthesized 96 cross-sectional surveys of medical students and found that attitudes toward AI and interest in curricular integration were generally favorable on average but highly variable across settings (Table 3). Favorable attitudes, perceived career benefit, and support for curricular integration were the most consistently endorsed domains, whereas self-reported familiarity and trust in AI-assisted decisions were endorsed less consistently and with greater between-study variability; because each domain draws on different studies, instruments, and denominators, these patterns are descriptive rather than a ranking across constructs (Table 3). These values should be interpreted as average summaries of nonequivalent self-report items rather than stable global prevalences (Table 3; Table S11 in Multimedia Appendix 2). The central message is therefore not that medical students uniformly accept AI but that receptivity, familiarity, concern, and trust vary substantially by context, technology, instrument, and respondent population (Table 3; Tables S6 and S11 in Multimedia Appendix 2). This broad pattern of favorable but uneven perceptions is directionally consistent with prior review-level evidence in health professions learners [12-14].

The prediction intervals are important for interpreting these results [16]. For several domains, the interval expected in a new comparable setting spanned from low endorsement to near-universal endorsement, even when the CI around the average summary was relatively narrow [16]. This distinction addresses a key limitation of interpreting the pooled mean alone: a precise-looking average can obscure wide between-setting variation [16]. In practical terms, a medical school should not assume that a global summary estimate reflects its own students and should compare the synthesis with a local baseline before curriculum planning, implementation, or evaluation [16,131]. In several domains, the dispersion of true effects is large enough that the summary estimate has limited stand-alone interpretive value and is best interpreted together with its prediction interval [16].

### Comparison With Prior Work

The findings are broadly consistent with prior reviews showing interest in AI among health professions learners [12-14], but this review narrows the inference to students in medical education programs and adds several safeguards that were not consistently present in earlier work [12-14]. We reviewed mixed populations, documented disaggregation decisions, harmonized nonidentical survey items into operational domains, assessed participant independence, used HKSJ-adjusted CIs, reported prediction intervals, and applied the GRADE framework to proportional self-report outcomes [15,16,29-31]. These steps improve transparency while making the limitations of the evidence more explicit, which is central to responsible interpretation of heterogeneous review findings [16,18,29-31]. First, the review provides medical education–specific benchmark estimates in which between-setting dispersion is displayed rather than hidden, so curriculum committees can see both the central tendency and the realistic range they may encounter [16]. Second, the harmonization taxonomy, mixed population disaggregation log, and participant independence audit are reusable procedures that future syntheses of heterogeneous survey evidence can adopt directly, complementing standardized review process reporting under PRISMA 2020 [18]. Third, by documenting where construct equivalence breaks down, the review specifies a measurement agenda that separates exposure, perceived familiarity, conceptual knowledge, and trust, and supports greater use of validated instruments such as MAIRS-MS [7,23].

Recent medical education literature argues that learners need foundational AI concepts and professionally relevant competencies [132]. Ethical reviews highlight bias, privacy, accountability, authorship, and responsible use as substantive educational concerns [133]. Critical-appraisal guidance also emphasizes validation, model limitations, and uncertainty when evaluating AI-enabled evidence [134]. Reviews of AI and generative AI in medical education describe rapidly expanding educational uses and the need for deliberate curricular responses [135,136]. Curriculum-focused literature further emphasizes structured training, critical appraisal, and human oversight [137-140]. Our findings are consistent with this direction: favorable attitudes and interest in curricular exposure coexisted with uneven self-reported familiarity and trust (Table 3). This pattern can inform educational needs assessment, but cross-sectional perception data do not demonstrate that any specific curriculum improves competence, trust calibration, ethical reasoning, or patient care outcomes [131,139]. A recent Best Evidence Medical Education scoping review similarly documented rapid growth in AI-related publications in medical education, concentrated in undergraduate training, and mapped priorities for the evaluative research the field still needs [141]. By quantifying the perception layer of this literature while displaying its uncertainty, our synthesis clarifies what the current descriptive evidence can and cannot support as those evaluative studies are developed [16,141].

This difference from prior reviews is clinically and educationally relevant [6,12-14]. Mixed health profession syntheses can be useful for mapping broad interest in AI, but medical students are still developing foundational clinical reasoning and evidence appraisal practices while learning how technology is incorporated into supervised clinical work [6,132,137-139]. Educational priorities at this stage therefore cannot be assumed to mirror those of practicing physicians, dental students, nursing students, or faculty [6,12-14,132]. A medical student–specific synthesis provides a more relevant evidence base for undergraduate medical education planning, while the wide between-setting dispersion still requires local adaptation [16,131]. For curriculum committees and other educational decision makers, an estimate anchored to a clearly defined learner population and accompanied by explicit construct mapping and prediction intervals is more actionable than a broad average whose constituent populations, instruments, and settings cannot be separated [16,23,131]. Accordingly, the harmonization documentation and prediction intervals are presented alongside the summary estimates rather than left as technical appendices [16,23].

The methodological contrast with prior syntheses is consequential rather than merely technical [12-16]. The only previous meta-analysis in this area pooled attitude and knowledge outcomes across medical, dental, and nursing students and did not apply HKSJ-adjusted CIs or report prediction intervals [14-16]. In the present review, the 95% prediction intervals were broad across all 9 domains—for example, 42.2%‐98.3% for positive attitude and 7.8%‐100% for self-reported familiarity—showing that a pooled mean may be a poor proxy for the true proportion in a new comparable setting (Table 3) [16,142]. This changes how the evidence should be used: pooled values are contextual benchmarks, not transportable targets, and should be compared with local needs assessments before curricular priorities are selected [16,131]. In practice, a local needs assessment showing strong support for curricular integration but limited familiarity could support prioritizing foundational concepts and supervised use, whereas high exposure accompanied by poorly calibrated reliance could indicate a greater need for critical appraisal, model limitations, and trust calibration [131,132,139]. These examples illustrate decision pathways for locally measured profiles, not associations between pooled domains, because each domain includes a different set of studies (Table 3). Medical student–only eligibility and explicit construct mapping reduce population and measurement ambiguity [7,23], while GRADE makes the very low certainty of each domain estimate transparent [29-31]. Together, these features move the field from broad descriptions of enthusiasm toward testable, context-specific curriculum planning: pilots can target documented local gaps and assess domain-specific competencies rather than satisfaction alone [131,132,139,141]. Because all included studies were cross-sectional, these methodological features do not show that educational or patient outcomes have already improved (Table 1) [17]. Their practical contribution is instead to strengthen the evidence base for designing, targeting, and evaluating interventions intended to improve those outcomes [131,139,141]. We regard these differences not as criticism of earlier contributions but as the methodological maturation of evidence synthesis in a rapidly developing field [12-16,141].

### Heterogeneity, Subgroup Analyses, Meta-Regression, and Sensitivity Findings

The 95% prediction intervals were wide across all proportional domains and, for several outcomes, spanned low to near-universal endorsement, indicating that the true proportion expected in a new comparable setting may differ markedly from the pooled mean (Table 3) [16,142]. Following Borenstein [16], we therefore interpret the practical magnitude of between-setting heterogeneity from the prediction intervals rather than from *I*². *I*² and τ² remain reported in Table 3 as complementary model statistics, but neither *I*² thresholds nor the *I*² range are used to characterize how widely true proportions vary across settings [16]. The observed dispersion may reflect contextual differences in country, institutional environment, clinical exposure, prior AI training, and survey period, as suggested by the diversity described in prior reviews and primary studies [10,12-14,107,116,128]. It also plausibly reflects measurement heterogeneity: studies used different item wording, Likert thresholds, handling of neutral responses, and AI referents ranging from broad AI to radiology algorithms and generative AI [7,10,12-14,23]. The harmonization table is therefore not a cosmetic supplement; it is necessary for understanding what was actually pooled and where construct equivalence becomes uncertain [23,28]. The wide prediction intervals provided the empirical rationale for the prespecified subgroup analyses and feasibility-screened meta-regressions: they demonstrate that true proportions are expected to vary widely across comparable settings, while not identifying the causes of that variation by themselves [16,142].

The subgroup analyses should be interpreted as descriptive probes rather than explanatory models, and the most consistent signal involved the type of AI asked about [143]. Studies framed around ChatGPT or other generative AI reported higher self-reported familiarity (77.4%) than studies of broad or general AI (60.9%) or domain-specific applications (45.1%; *P*=.008; Table 4), which is plausible given the rapid diffusion of generative tools in medical education after late 2022 [11,135,141]. At the same time, ChatGPT or LLM-focused studies reported lower positive attitude (66.7%) than broad-AI (80.1%) or domain-specific studies (83.2%; *P*=.005; Table 4). One cautious interpretation is that direct exposure may coexist with more tempered enthusiasm as students encounter limitations, ethical ambiguities, and academic integrity concerns described in the generative-AI literature [11,133,136,144]. Concern about physician replacement was lower in studies of domain-specific applications (27.2%) than in studies of broad AI (45.9%) or generative AI (43.4%; *P*=.01; Table 4); this pattern is compatible with prior work showing that the framing and intended role of AI can shape student attitudes and professional concerns [10,122,128]. Self-reported familiarity was also higher in studies published from 2023 onward than before 2023 (66.4% vs 45.6%; *P*=.04; Table 4), a pattern temporally consistent with wider generative-AI exposure [11,135,141]. All of these are study-level associations that meet few of the established credibility criteria for subgroup effects: they are observational, rest on between-study rather than within-study comparisons, involve multiple tests without multiplicity adjustment, and concern moderators that are correlated with region, language, curriculum, sampling strategy, and survey instrument [143]. They are therefore best read as coherent, hypothesis-generating patterns rather than established effects [143].

Equally informative are the moderators for which no statistically significant subgroup difference was detected (Table 4). The displayed analyses provided no clear evidence of differences by World Bank income level or JBI risk-of-bias category (Table 4). These null findings do not establish equivalence and may reflect limited subgroup power; accordingly, they should not be used either to assert a universal income-level pattern or to conclude that risk-of-bias category has no moderating role [143]. Prespecified feasibility rules further limited unstable moderator comparisons: of 36 candidate domain-moderator combinations, 20 were analyzed and 16 were not run, most commonly because a moderator level contained fewer than 3 studies or because a domain had fewer than 10 studies (Tables S13 and S14 in Multimedia Appendix 2). These decisions are reported in full so that absence of an analysis is not mistaken for absence of an effect, consistent with cautious interpretation of subgroup evidence [143]. Leave-one-out analyses address a different question—the influence of individual studies on the pooled summaries—and no single omission changed the interpretation of any domain; the maximum shift was 6.7 percentage points for trust, the smallest domain (*k*=8), and 2.8 percentage points or less for the remaining 8 domains (Table 5; Figures S28-S36 in Multimedia Appendix 2).

The meta-regressions provide a complementary study-level analysis of moderator patterns (Tables S13 and S14 in Multimedia Appendix 2). Of the 20 feasibility-screened univariable models, 5 were statistically significant and identified the same 2 moderators as the categorical comparisons: AI type (for positive attitude, perceived career benefit, replacement concern, and familiarity) and publication period (for familiarity) (Table S13 in Multimedia Appendix 2). This convergence is internally coherent, but it does not upgrade the causal status of either moderator because meta-regression on study-level summaries remains vulnerable to aggregation bias and confounding by correlated study characteristics [17,143]. The number of studies per moderator level was sometimes small, and nominal *P* values were not adjusted for multiplicity [143]. The bubble plots and full model listings are therefore provided in Tables S13 and S14 and Figures S50-S54 in Multimedia Appendix 2, and our interpretation is confined to the descriptive statement that the AI referent and survey period are candidate axes for future individual-level studies of effect modification [143].

Finally, small study effects deserve explicit discussion because they feed directly into the certainty assessment [29-31]. Egger regression tests on logit proportions indicated funnel asymmetry for 5 of the 8 testable domains: positive attitude, curricular integration, familiarity, prior AI use, and willingness to learn or adopt AI; no asymmetry was detected for career benefit, replacement concern, or ethical concerns, and the test was not performed for trust (*k*=8; Table 5; Table S16 and Figures S37-S44 in Multimedia Appendix 2). In proportion meta-analyses, funnel asymmetry cannot be equated with publication bias because heterogeneity, instrument differences, sampling frames, and outcome definitions can produce the same signature [26,129,130]. These findings were therefore treated in the GRADE assessment as a possible publication bias signal rather than as demonstrated bias; they contribute to, but are not the sole reason for, the very low certainty ratings (Table 6; Table S17 in Multimedia Appendix 2) [29-31].

### Risk of Bias, Certainty, and Outcome Harmonization

Risk-of-bias findings should also be read conservatively in light of the design and measurement limitations of cross-sectional survey evidence [12-14,24]. No study met our review-defined high-risk threshold (Table 2), but this does not mean that the evidence base is methodologically strong. Voluntary cross-sectional surveys, convenience sampling, locally adapted questionnaires, uncertain response rate denominators, and self-selection are recurring limitations in this literature and can constrain representativeness and measurement validity [12-14,24]. The item-level pattern in our review (Table 2) locates these weaknesses precisely: inclusion criteria, outcome measurement, and statistical analysis were adequately reported in all 96 studies; confounding factors were identified in only 17 studies (18%) and addressed in 15 (16%); no study was rated Yes on JBI item 4, which asks whether objective, standard criteria were used to measure the condition; and exposure measurement was unclear in 18 studies (19%). For the perception-based outcomes synthesized here, the item 4 pattern should be interpreted in the context of what the checklist asks rather than as evidence that the surveys measured an objective clinical condition (Table 2) [24]. These limitations are especially important because the outcomes are perceptions and self-reported familiarity rather than objective literacy or behavior [7,23,132,139].

For this reason, GRADE certainty was very low across domains (Table 6; Table S17 in Multimedia Appendix 2). Downgrading was driven not only by risk of bias but also by inconsistency, indirectness, and imprecision [29-31]. The very wide prediction intervals were central to the inconsistency and imprecision judgments because they indicate substantial uncertainty about the true proportion expected in a new comparable setting [16,30,31]. Indirectness arose because domains such as familiarity, positive attitude, ethical concern, and trust were measured with nonequivalent items that required operational harmonization [23,30]. Publication bias was difficult to separate from other sources of funnel asymmetry, but the observed small study effects in several domains were treated as an additional reason for caution rather than as proof of selective publication [26,129,130].

A related issue is that self-reported familiarity should not be treated as equivalent to AI literacy [7,23,132]. Exposure to ChatGPT or diagnostic AI does not by itself demonstrate understanding of model development, validation, calibration, data drift, fairness, privacy, or clinical accountability—competencies emphasized in AI-literacy and professional-use frameworks [23,132,134,139,145]. Conversely, students with limited exposure may still hold well-founded concerns about safety and professional responsibility [133,144,145]. Future studies should therefore distinguish exposure, perceived familiarity, conceptual knowledge, applied appraisal skills, and behavior [23,132,139]. Validated instruments such as MAIRS-MS [7] are helpful, but the field also needs practical assessments of whether students can recognize unreliable AI outputs, identify unsupported claims, and decide when clinician oversight is required [132,134,139]. The continuous MAIRS-MS syntheses likewise showed variation in study-level estimates across settings despite the use of a validated instrument [7]; accordingly, the pooled means are treated as secondary descriptive summaries rather than transportable estimates of readiness (Table S10 and Figures S45-S49 in Multimedia Appendix 2).

### Implications for AI Literacy Curriculum Development

The curricular implication is exploratory and context-dependent because the available evidence describes perceptions rather than intervention effects [12-14,141]. The findings suggest the need to consider locally adapted AI literacy curricula that address core terminology, common clinical and educational use cases, critical appraisal of AI-enabled evidence, data bias, privacy, accountability, uncertainty, and appropriate human oversight [132,134,139]. Curriculum design should also distinguish between AI used for learning support and AI used in clinical decision-making because the educational aims, disclosure expectations, and safety consequences differ across these contexts [11,136,144,145]. Learning support use may emphasize appropriate prompting, verification of generated content, academic integrity, and transparent disclosure [11,136,144], whereas clinical use requires attention to validation, workflow, responsibility, patient communication, and professional judgment [132,134,139,145].

Trust calibration could reasonably be treated as a specific curricular goal because safe AI use requires neither reflexive acceptance nor reflexive rejection but judgment about when an output is sufficiently reliable for a particular task [139,145]. Trust in AI-assisted decisions may refer to distinct constructs—trust in diagnostic accuracy, willingness to follow AI recommendations, trust in opaque systems, or trust in specific clinical applications—and prior student surveys likewise show that attitudes vary with the type and role of AI being considered [10,98,122,128]. A low or moderate summary estimate for trust should therefore not be interpreted simply as resistance to innovation, and a high estimate should not be assumed to reflect calibrated confidence [139,145]. Students need to learn when AI outputs may be useful, when they may be misleading, and how to verify claims against clinical evidence and patient context [132,134,139]. Educational interventions should therefore measure not only attitudes but also applied competencies, such as recognizing model limitations, identifying biased outputs, communicating uncertainty, and maintaining clinical reasoning while using AI tools [139].

Ethical concern should likewise be interpreted constructively because current ethical guidance for AI in health and health professions education treats bias, transparency, privacy, authorship, accountability, and professional responsibility as substantive competencies rather than peripheral concerns [133,144,145]. The relatively high endorsement of ethical concerns in our synthesis may therefore identify curricular topics that require explicit discussion rather than resistance that needs to be overcome, an interpretation consistent with this guidance [133,144,145]. Curricula should avoid a purely promotional framing and instead help students understand when AI tools are useful, what evidence is needed before clinical deployment, how patient values and local context affect decisions, and why professional responsibility cannot simply be delegated to an algorithm [132,139,144,145].

The distinction between educational use and clinical use also matters for policy because these contexts carry different requirements for assessment integrity, validation, accountability, and patient safety [11,139,144,145]. Generative AI may support explanation, formative feedback, simulation, drafting, and self-study [11,135,136,144], whereas diagnostic decision support or treatment recommendation creates different requirements for validation, accountability, and patient safety [132,134,139,145]. Medical schools may therefore need tiered guidance, aligned with ethical and governance frameworks for AI in health and health professions education, that separates acceptable learning assistance, disclosure expectations, assessment integrity, supervision, and clinical safety [133,144,145]. Because the present evidence is based on perceptions, these policy implications should be tested through curriculum pilots, objective performance measures, longitudinal follow-up, and evaluation of unintended consequences such as automation bias or reduced independent reasoning [11,139,141].

### Strengths and Limitations

This review has several methodological strengths that are directly relevant to random-effects evidence synthesis and certainty assessment [15,16,29-31,142]. It used a medical student–specific eligibility definition, reviewed mixed population studies, documented participant independence checks, harmonized outcome domains transparently, and conducted proportional meta-analyses with HKSJ-adjusted CIs and prediction intervals [15,16,142]. The synthesis also reports subgroup analyses, feasibility-screened meta-regression, leave-one-out sensitivity analyses, small study effect tests, risk of bias, and GRADE certainty [29-31]. Together, these features help distinguish the pooled mean from the between-setting dispersion that is central to interpreting a random-effects meta-analysis [16,142].

Limitations remain substantial because the evidence base consists entirely of cross-sectional self-report studies, which cannot establish changes over time, causal effects of AI exposure, or curriculum effectiveness [12-14]. Survey instruments, AI referents, response scales, and dichotomization rules varied widely, a recurring challenge in this literature [7,12-14,23]. Some mixed population studies required disaggregation, and some potentially relevant data were nonextractable (Tables S3-S5 in Multimedia Appendix 2). Meta-regression was limited by study-level confounding and multiple testing, which constrains causal interpretation of moderator findings [17,143]. Google Scholar and citation search reproducibility depended on the search records available to the review team; transparent reporting of such search process limitations is particularly important for systematic review reproducibility [19]. Finally, trust in AI-assisted decisions was supported by fewer than 10 studies and should be treated as especially uncertain (Tables 3 and 6). More broadly, meta-analysis of cross-sectional perception data has inherent interpretive limits even when conducted rigorously: pooling can quantify and display variation across settings, but it cannot convert nonequivalent self-report items into a single transportable prevalence [16,28].

### Conclusions

Among medical students, AI-related attitudes and interest in curricular integration appear generally favorable, but familiarity, trust, and ethical concern vary substantially across settings. The principal contribution of this review is not a single transportable prevalence estimate but a medical student–specific synthesis that makes outcome harmonization, mixed population disaggregation, participant independence checks, between-setting dispersion, risk of bias, and certainty explicit. The findings support locally tailored AI literacy needs assessment and curriculum development, while not establishing the effectiveness of any particular curriculum. Future studies should use standardized, validated measures; report medical student data separately; distinguish perceived familiarity from objective competence; preserve item wording and neutral response handling; and evaluate educational interventions with longitudinal and performance-based outcomes. Until stronger evidence is available, curriculum planning should be treated as context-sensitive competency development rather than as a response to a presumed universal prevalence.

## Supplementary material

10.2196/89411 Multimedia Appendix 1 Complete search strategies for all 6 databases.

10.2196/89411 Multimedia Appendix 2 Supplementary study-level data and analyses, including study selection and eligibility documentation, study characteristics, risk-of-bias assessments, outcome extraction and harmonization, participant independence checks, subgroup and meta-regression analyses, sensitivity analyses, small study effects, GRADE (Grading of Recommendations, Assessment, Development and Evaluation) evidence profiles, and supplementary figures.

10.2196/89411 Checklist 1 PRISMA 2020 expanded checklist, PRISMA 2020 for Abstracts checklist, and PRISMA-S (Preferred Reporting Items for Systematic Reviews and Meta-Analyses literature search extension) checklist.

## Abbreviations

GRADE: Grading of Recommendations, Assessment, Development and Evaluation
HKSJ: Hartung-Knapp-Sidik-Jonkman
JBI: Joanna Briggs Institute
LLM: large language model
MAIRS-MS: Medical AI Readiness Scale for Medical Students
PRISMA: Preferred Reporting Items for Systematic Reviews and Meta-Analyses
PRISMA-S: Preferred Reporting Items for Systematic Reviews and Meta-Analyses literature search extension
WHO: World Health Organization
