# Supplementary material for: Medical Students’ Attitudes, Perceptions, and Self-Reported Familiarity With AI in Health Care: Systematic Review and Meta-Analysis
Source: JMIR Med Educ. 2026 Aug 18;12:e89411. doi: 10.2196/89411 (PMC13484950; doi:10.2196/89411)
Supplement: Supplementary file 1 [file mededu-v12-e89411-s001.docx]

## Multimedia Appendix 1. Complete search strategies for all 6 databases (PubMed/MEDLINE, Embase, Web of Science, Scopus, PsycINFO, Cochrane CENTRAL)

All databases were searched from database inception to **April 1, 2026**. No language restrictions were applied. Search strategies were structured around three concept blocks combined with Boolean **AND** logic: **(1) population: medical students; (2) exposure: artificial intelligence and related technologies; (3) outcomes: attitudes, perceptions, knowledge, and related constructs**. Controlled vocabulary and free-text terms were combined with Boolean **OR** within each concept block.

1. **PubMed/MEDLINE (via National Library of Medicine)**

(

"Students, Medical"[MeSH Terms]

OR "Education, Medical, Undergraduate"[MeSH Terms]

OR "Education, Medical, Graduate"[MeSH Terms]

OR "Clinical Clerkship"[MeSH Terms]

OR "medical student"[tiab]

OR "medical students"[tiab]

OR "medical undergraduate"[tiab]

OR "medical undergraduates"[tiab]

OR "undergraduate medical"[tiab]

OR "medical intern"[tiab]

OR "medical interns"[tiab]

OR "medical school"[tiab]

OR "medical schools"[tiab]

OR "medical education"[tiab]

OR "medical trainee"[tiab]

OR "medical trainees"[tiab]

OR "clinical clerkship"[tiab]

OR "clinical clerkships"[tiab]

OR "preclinical student"[tiab]

OR "preclinical students"[tiab]

OR "clinical student"[tiab]

OR "clinical students"[tiab]

OR "MBBS student"[tiab]

OR "MBBS students"[tiab]

)

AND

(

"Artificial Intelligence"[MeSH Terms]

OR "Machine Learning"[MeSH Terms]

OR "Deep Learning"[MeSH Terms]

OR "Neural Networks, Computer"[MeSH Terms]

OR "Natural Language Processing"[MeSH Terms]

OR "Decision Support Systems, Clinical"[MeSH Terms]

OR "artificial intelligence"[tiab]

OR "AI"[tiab]

OR "machine learning"[tiab]

OR "deep learning"[tiab]

OR "neural network"[tiab]

OR "neural networks"[tiab]

OR "natural language processing"[tiab]

OR "NLP"[tiab]

OR "large language model"[tiab]

OR "large language models"[tiab]

OR "LLM"[tiab]

OR "LLMs"[tiab]

OR "ChatGPT"[tiab]

OR "GPT-4"[tiab]

OR "GPT-3"[tiab]

OR "GPT4"[tiab]

OR "generative artificial intelligence"[tiab]

OR "generative AI"[tiab]

OR "clinical decision support"[tiab]

OR "chatbot"[tiab]

OR "chatbots"[tiab]

OR "intelligent system"[tiab]

OR "intelligent systems"[tiab]

OR "computer aided diagnosis"[tiab]

OR "computer assisted diagnosis"[tiab]

)

AND

(

"Attitude of Health Personnel"[MeSH Terms]

OR "Health Knowledge, Attitudes, Practice"[MeSH Terms]

OR "Attitude to Computers"[MeSH Terms]

OR "Surveys and Questionnaires"[MeSH Terms]

OR "attitude"[tiab]

OR "attitudes"[tiab]

OR "perception"[tiab]

OR "perceptions"[tiab]

OR "perceived"[tiab]

OR "knowledge"[tiab]

OR "awareness"[tiab]

OR "familiarity"[tiab]

OR "opinion"[tiab]

OR "opinions"[tiab]

OR "belief"[tiab]

OR "beliefs"[tiab]

OR "acceptance"[tiab]

OR "readiness"[tiab]

OR "willingness"[tiab]

OR "survey"[tiab]

OR "surveys"[tiab]

OR "questionnaire"[tiab]

OR "questionnaires"[tiab]

OR "cross-sectional"[tiab]

OR "perspective"[tiab]

OR "perspectives"[tiab]

OR "preparedness"[tiab]

OR "viewpoint"[tiab]

OR "viewpoints"[tiab]

OR "self-efficacy"[tiab]

)

AND

(

"1000/01/01"[Date - Publication] : "2026/04/01"[Date - Publication]

)

**2. Embase (via Elsevier Embase.com)**

Date limit applied in platform interface: database inception to April 1, 2026.

(

'medical student'/exp

OR 'medical education'/exp

OR 'clinical clerkship'/exp

OR 'medical student*':ti,ab,kw

OR 'medical undergraduate*':ti,ab,kw

OR 'undergraduate medical':ti,ab,kw

OR 'medical intern*':ti,ab,kw

OR 'medical school*':ti,ab,kw

OR 'medical education':ti,ab,kw

OR 'medical trainee*':ti,ab,kw

OR 'clinical clerkship*':ti,ab,kw

OR 'preclinical student*':ti,ab,kw

OR 'clinical student*':ti,ab,kw

OR 'mbbs student*':ti,ab,kw

)

AND

(

'artificial intelligence'/exp

OR 'machine learning'/exp

OR 'deep learning'/exp

OR 'neural network'/exp

OR 'natural language processing'/exp

OR 'clinical decision support system'/exp

OR 'chatbot'/exp

OR 'artificial intelligence':ti,ab,kw

OR 'ai':ti,ab,kw

OR 'machine learning':ti,ab,kw

OR 'deep learning':ti,ab,kw

OR 'neural network*':ti,ab,kw

OR 'natural language processing':ti,ab,kw

OR 'nlp':ti,ab,kw

OR 'large language model*':ti,ab,kw

OR 'llm':ti,ab,kw

OR 'llms':ti,ab,kw

OR 'chatgpt':ti,ab,kw

OR 'gpt-4':ti,ab,kw

OR 'gpt-3':ti,ab,kw

OR 'gpt4':ti,ab,kw

OR 'generative artificial intelligence':ti,ab,kw

OR 'generative ai':ti,ab,kw

OR 'clinical decision support':ti,ab,kw

OR 'chatbot*':ti,ab,kw

OR 'intelligent system*':ti,ab,kw

OR 'computer aided diagnos*':ti,ab,kw

OR 'computer assisted diagnos*':ti,ab,kw

)

AND

(

'health personnel attitude'/exp

OR 'attitude to computer'/exp

OR 'questionnaire'/exp

OR 'knowledge'/exp

OR 'attitude*':ti,ab,kw

OR 'perception*':ti,ab,kw

OR 'perceived':ti,ab,kw

OR 'knowledge':ti,ab,kw

OR 'awareness':ti,ab,kw

OR 'familiarity':ti,ab,kw

OR 'opinion*':ti,ab,kw

OR 'belief*':ti,ab,kw

OR 'acceptance':ti,ab,kw

OR 'readiness':ti,ab,kw

OR 'willingness':ti,ab,kw

OR 'survey*':ti,ab,kw

OR 'questionnaire*':ti,ab,kw

OR 'cross-sectional':ti,ab,kw

OR 'perspective*':ti,ab,kw

OR 'viewpoint*':ti,ab,kw

OR 'preparedness':ti,ab,kw

OR 'self-efficacy':ti,ab,kw

)

**3. Web of Science Core Collection (via Clarivate)**

Date limit applied in platform interface: database inception to April 1, 2026.

TS=("medical student" OR "medical students" OR "medical undergraduate"

OR "medical undergraduates" OR "undergraduate medical" OR "medical intern"

OR "medical interns" OR "medical school" OR "medical schools"

OR "medical education" OR "medical trainee" OR "medical trainees"

OR "clinical clerkship" OR "preclinical student" OR "preclinical students"

OR "MBBS student" OR "MBBS students")

AND

TS=("artificial intelligence" OR "machine learning" OR "deep learning"

OR "neural network" OR "neural networks" OR "natural language processing"

OR "large language model" OR "large language models" OR "ChatGPT"

OR "GPT-4" OR "GPT-3" OR "generative AI" OR "generative artificial intelligence"

OR "clinical decision support" OR "chatbot" OR "chatbots"

OR "intelligent system" OR "intelligent systems" OR "AI" OR "NLP" OR "LLM")

AND

TS=("attitude" OR "attitudes" OR "perception" OR "perceptions" OR "perceived"

OR "knowledge" OR "awareness" OR "familiarity" OR "opinion" OR "opinions"

OR "belief" OR "beliefs" OR "acceptance" OR "readiness" OR "willingness"

OR "survey" OR "surveys" OR "questionnaire" OR "questionnaires"

OR "cross-sectional" OR "perspective" OR "perspectives"

OR "preparedness" OR "viewpoint" OR "self-efficacy")

**4. Scopus (via Elsevier)**

Date limit applied in platform interface: database inception to April 1, 2026.

TITLE-ABS-KEY("medical student" OR "medical students" OR "medical undergraduate"

OR "medical undergraduates" OR "undergraduate medical" OR "medical intern"

OR "medical interns" OR "medical school" OR "medical schools"

OR "medical education" OR "medical trainee" OR "medical trainees"

OR "clinical clerkship" OR "preclinical student" OR "preclinical students"

OR "MBBS student" OR "MBBS students")

AND

TITLE-ABS-KEY("artificial intelligence" OR "machine learning" OR "deep learning"

OR "neural network" OR "neural networks" OR "natural language processing"

OR "large language model" OR "large language models" OR "ChatGPT"

OR "GPT-4" OR "GPT-3" OR "generative AI" OR "generative artificial intelligence"

OR "clinical decision support" OR "chatbot" OR "chatbots"

OR "intelligent system" OR "intelligent systems" OR "AI" OR "NLP" OR "LLM")

AND

TITLE-ABS-KEY("attitude" OR "attitudes" OR "perception" OR "perceptions"

OR "perceived" OR "knowledge" OR "awareness" OR "familiarity"

OR "opinion" OR "opinions" OR "belief" OR "beliefs" OR "acceptance"

OR "readiness" OR "willingness" OR "survey" OR "surveys"

OR "questionnaire" OR "questionnaires" OR "cross-sectional"

OR "perspective" OR "perspectives" OR "preparedness" OR "viewpoint"

OR "self-efficacy")

**5. APA PsycINFO (via Ovid)**

Date limit applied in platform interface: database inception to April 1, 2026.

((Medical Students/ or ("medical student*" or "medical undergraduate*" or "medical intern*" or "medical school*" or "medical education" or "medical trainee*" or "MBBS student*" or "clinical clerkship*").ti,ab.) AND (exp Artificial Intelligence/ or Machine Learning/ or Deep Learning/ or Neural Networks/ or Natural Language Processing/ or Chatbots/ or ("artificial intelligence" or "machine learning" or "deep learning" or "neural network*" or "natural language processing" or "large language model*" or "ChatGPT" or "GPT-4" or "generative AI" or "clinical decision support" or "chatbot*" or "AI" or "NLP" or "LLM").ti,ab.) AND (Attitudes/ or Health Knowledge/ or Student Attitudes/ or Self Efficacy/ or Perception/ or ("attitude*" or "perception*" or "knowledge" or "awareness" or "familiarity" or "opinion*" or "belief*" or "acceptance" or "readiness" or "survey*" or "questionnaire*" or "cross-sectional" or "perspective*").ti,ab.))

**6. Cochrane CENTRAL (via Cochrane Library)**

Date limit applied in platform interface: database inception to April 1, 2026.

("medical student" OR "medical students" OR "medical education")

AND

("artificial intelligence" OR "machine learning" OR "deep learning"

OR "ChatGPT" OR "large language model" OR "AI")

AND

("attitude" OR "perception" OR "knowledge" OR "awareness" OR "survey"

OR "questionnaire" OR "cross-sectional")

**Supplementary Table. Record counts by database**

| **Database** | **Platform** | **Search period** | **Records retrieved (before deduplication)** |
| --- | --- | --- | --- |
| PubMed/MEDLINE | National Library of Medicine | Inception to 2026-04-01 | 2,387 |
| Embase | Elsevier Embase.com | Inception to 2026-04-01 | 4,132 |
| Web of Science Core Collection | Clarivate | Inception to 2026-04-01 | 1,617 |
| Scopus | Elsevier | Inception to 2026-04-01 | 4,494 |
| APA PsycINFO | Ovid | Inception to 2026-04-01 | 204 |
| Cochrane CENTRAL | Cochrane Library | Inception to 2026-04-01 | 97 |
| **Total** |  |  | **12,931** |

Note. All databases were searched from inception to April 1, 2026. Counts shown are the numbers of records retrieved from each database before deduplication. The total number of unique records after deduplication is reported in the PRISMA flow diagram (Figure1).
