# Supplementary material for: Medical Students’ Attitudes, Perceptions, and Self-Reported Familiarity With AI in Health Care: Systematic Review and Meta-Analysis
Source: JMIR Med Educ. 2026 Aug 18;12:e89411. doi: 10.2196/89411 (PMC13484950; doi:10.2196/89411)
Supplement: Supplementary file 2 [file mededu-v12-e89411-s002.docx]

**Multimedia Appendix 2**

**Table S1. Sequential database deduplication record using EndNote 21.**

| **Step** | **Database** | **Records Imported** | **Duplicates Found** | **Unique Retained** |
| --- | --- | --- | --- | --- |
| 1 | PubMed/MEDLINE (base) | 2,387 | 0 | 2,387 |
| 2 | + Embase | 4,132 | 2,814 | 3,705 |
| 3 | + Web of Science | 1,617 | 1,203 | 4,119 |
| 4 | + Scopus | 4,494 | 2,457 | 6,156 |
| 5 | + PsycINFO | 204 | 126 | 6,234 |
| 6 | + Cochrane CENTRAL | 97 | 49 | 6,282 |
| 7 | + Other sources | 77 | 0 | 6,359 |
| Total |  | 13,008 | 6,649 | 6,359 |

*Deduplication used automated DOI, title, author, year, and journal matching followed by manual verification.*

**Table S2. Reports not retrieved and reports excluded after eligibility assessment.**

| **PRISMA category or reason** | **No. of studies** | **% of total** |
| --- | --- | --- |
| Mixed population; data not disaggregable for medical students | 27 | 39.1 |
| Reports not retrieved after exhaustive retrieval attempts | 14 | 20.3 |
| Educational intervention or AI tool evaluation study | 10 | 14.5 |
| Non-medical discipline only (nursing, pharmacy, dentistry) | 4 | 5.8 |
| Qualitative only; no quantitative survey data | 3 | 4.3 |
| Not about AI / scale validation study / out of scope | 4 | 5.8 |
| Sampling, selection, or reporting bias | 3 | 4.4 |
| No identifiable medical student population | 2 | 2.9 |
| Other | 2 | 2.9 |
| Total | 69 | 100.0 |

**Table S3. Exclusion-impact comparison after removing nonseparable mixed-population records.**

Excluded records from all proportion-domain syntheses.

| **No.** | **Study ID** | **First author** | **Year** | **Action** | **Reason** |
| --- | --- | --- | --- | --- | --- |
| 25 | Sami 2025 a | Abdul Sami | 2025 | Excluded from all proportion domains | Non-separable MBBS+BDS dental data |
| 42 | Farooq and Usmani 2025 a | Muhammad Farooq | 2025 | Excluded from all proportion domains | Non-separable MBBS+BDS+allied health data |

Domain-level comparison of the initial 98-study synthesis and final 96-study synthesis.

| **Domain ID** | **Domain** | **Old k** | **Old N** | **Old pooled 95% CI** | **New k** | **New N** | **New pooled 95% CI** | **Delta k** | **Delta N** | **Delta pooled pp** |
| --- | --- | --- | --- | --- | --- | --- | --- | --- | --- | --- |
| P1 | Positive attitude toward AI | 46 | 21660 | 76.9% (72.4% to 81.2%) | 44 | 20806 | 76.9% (72.2% to 81.4%) | -2 | -854 | 0.01 |
| P2 | AI perceived as beneficial to career | 17 | 9951 | 77.9% (69.5% to 85.3%) | 16 | 9799 | 78.4% (69.5% to 86.2%) | -1 | -152 | 0.50 |
| P3 | Support for AI curricular integration | 40 | 17162 | 76.3% (71.6% to 80.7%) | 38 | 16308 | 76.6% (71.8% to 81.1%) | -2 | -854 | 0.31 |
| P4 | Concern about physician replacement | 34 | 17496 | 40.4% (34.4% to 46.6%) | 32 | 16642 | 39.9% (33.6% to 46.5%) | -2 | -854 | -0.46 |
| P5 | Self-reported familiarity/knowledge | 53 | 27969 | 63.9% (56.6% to 70.9%) | 52 | 27817 | 63.3% (55.9% to 70.3%) | -1 | -152 | -0.63 |
| P6 | Prior AI use | 37 | 18076 | 63.1% (53.4% to 72.3%) | 36 | 17374 | 63.2% (53.2% to 72.6%) | -1 | -702 | 0.10 |
| P7 | Willingness to learn/adopt AI | 22 | 9199 | 71.5% (64.8% to 77.8%) | 22 | 9199 | 71.5% (64.8% to 77.8%) | 0 | 0 | 0.00 |
| P8 | Ethical concerns about AI | 29 | 14723 | 62.6% (54.0% to 70.8%) | 28 | 14571 | 62.8% (53.9% to 71.3%) | -1 | -152 | 0.21 |
| P9 | Trust in AI-assisted decisions | 9 | 3709 | 51.5% (32.2% to 70.5%) | 8 | 3007 | 50.6% (28.5% to 72.6%) | -1 | -702 | -0.86 |

**Table S4. Mixed-population records assessed for separability of medical-student data**

Note. These are title/recruitment screening flags, not exclusions; adjudicated decisions and full documentation are in Table S5, and the two studies ultimately excluded (Sami 2025; Farooq and Usmani [2025]) are listed in Table S3.

| No. | Study ID | Article title | No. of medical students | Review decision |
| --- | --- | --- | --- | --- |
| 9 | Catan-Inan 2026 a | Artificial intelligence literacy and readiness in future health care professionals: a cross-sectional study | 183 | Confirmed mixed population; see Table S5 |
| 13 | Chakri 2025 a | Knowledge and Perceptions of AI Among Medical Students in Morocco: Cross-Sectional Study | 580 | Not a mixed population on full-text review; single medical-student population |
| 21 | Onetiu 2025 a | Assessing Medical Students’ Perceptions of AI-Integrated Telemedicine: A Cross-Sectional Study in Romania | 161 | Not a mixed population on full-text review; single medical-student population |
| 25 | Sami 2025 a | Medical students’ attitudes toward AI in education: perception, effectiveness, and its credibility | 595 | Confirmed mixed population; see Table S5; ultimately excluded and listed in Table S3. |
| 37 | Ejas 2025 a | Medical Students’ Perceptions of Large Language Models in Healthcare: A Multinational Cross-Sectional Study | 1180 | Not a mixed population on full-text review; single medical-student population |
| 38 | Mousavi Baigi 2025 a | Medical and Paramedical Students’ Perspectives on Artificial Intelligence in a Low- and Middle-Income Country: A Cross-Sectional Study | 95 | Confirmed mixed population; see Table S5 |
| 42 | Farooq and Usmani 2025 a | Artificial Intelligence in Medical Education | 92 | Confirmed mixed population; see Table S5; ultimately excluded and listed in Table S3. |
| 43 | AlZaabi 2025 a | Assessing medical students’ readiness for artificial intelligence after pre-clinical training | 84 | Not a mixed population on full-text review; single medical-student population |
| 49 | Dhurandhar 2025 a | Exploring Medical Artificial Intelligence Readiness Among Future Physicians: Insights From a Medical College in Central India | 482 | Not a mixed population on full-text review; single medical-student population |
| 50 | Allam 2024 b | Knowledge, attitude, and perception of Arab medical students towards artificial intelligence in medicine and radiology: A multi‑national cross‑sectional study‑‑ | 4492 | Not a mixed population on full-text review; single medical-student population |
| 59 | Amin 2024 a | Knowledge, attitude, and practice of artificial intelligence among medical students in Sudan: a cross-sectional study | 762 | Not a mixed population on full-text review; single medical-student population |
| 74 | Al-Roomi 2024 a | Familiarity and Applications of Artificial Intelligence in Health Professions Education: Perspectives of Students in a Community-Oriented Medical School | 152 | Not a mixed population on full-text review; single medical-student population |
| 77 | Tangadulrat 2023 a | Using ChatGPT for Clinical Practice and Medical Education: Cross-Sectional Survey of Medical Students’ and Physicians’ Perceptions | 124 | Confirmed mixed population; see Table S5 |
| 78 | Oluwadiya 2023 a | Exploring Artificial Intelligence in the Nigerian Medical Educational Space: An Online Cross-sectional Study of Perceptions, Risks and Benefits among Students and Lecturers from Ten Universities | 708 | Confirmed mixed population; see Table S5 |
| 81 | Al Hadithy 2023 a | Knowledge, Attitudes, and Perceptions of Artificial Intelligence in Healthcare Among Medical Students at Sultan Qaboos University | 221 | Not a mixed population on full-text review; single medical-student population |
| 83 | Barreiro-Ares 2023 a | Impact of the Rise of Artificial Intelligence in Radiology: What Do Students Think? | 281 | Not a mixed population on full-text review; single medical-student population |
| 86 | Ahmed 2022 a | Knowledge, attitude, and practice of artificial intelligence among doctors and medical students in Pakistan: A cross-sectional online survey | 247 | Confirmed mixed population; see Table S5 |
| 87 | Liu 2022 a | Perceptions of US Medical Students on Artificial Intelligence in Medicine: Mixed Methods Survey Study | 390 | Not a mixed population on full-text review; single medical-student population |
| 93 | Boillat 2022 a | Readiness to Embrace Artificial Intelligence Among Medical Doctors and Students: Questionnaire-Based Study | 102 | Confirmed mixed population; see Table S5 |
| 95 | Bisdas 2021 a | Artificial Intelligence in Medicine: A Multinational Multi-Center Survey on the Medical and Dental Students’ Perception | 2495 | Confirmed mixed population; see Table S5 |

**Table S5. Full mixed-population documentation and separability decisions.**

| **Study** | **Participant groups** | **Disaggregation possible (Y/N)** | **How medical-student data were extracted** | **Excluded non-medical groups** | **Synthesis decision** |
| --- | --- | --- | --- | --- | --- |
| 3. Jamjoom 2026  Perception of integrating an AI teaching module into medical education curriculum | Faculty members (n=68), teaching assistants (n=16), and medical students (n=117). | Y | Academic role was reported; medical-student subset identifiable. No numeric proportion-domain event/total values were contributed by this study. Source: Abstract/Methods: role-stratified survey of 201 participants; roles listed as faculty, teaching assistants, and medical students. | Faculty and teaching assistants excluded from medical-student denominators; no proportion-domain k/N impact. | Included in the systematic review but contributed no pooled outcome |
| 8. Yousef 2026  Artificial intelligence in ophthalmology: trust, bias, and responsibility from the perspective of medical students and ophthalmologists | Medical students (n=353) and ophthalmologists (n=172). | Y | Participant role was reported and results tables compared students with ophthalmologists; medical-student-specific denominators used for extracted proportions. Source: Results/participant demographics and Table 2: 353 medical students and 172 ophthalmologists; outcome comparisons by role. | Ophthalmologists excluded; P1/P4/P7/P8 use n total=353. | Included in the systematic review and quantitative synthesis |
| 9. Catan-Inan 2026  Artificial intelligence literacy and readiness in future health care professionals: a cross-sectional study | Medicine students (n=183) and nursing students (n=260), total n=443. | Y | Department was reported and MAIRS/AILS scores were reported by department. No numeric proportion-domain event/total values were contributed by this study. Source: Methods: Faculty of Medicine and Department of Nursing sample; Table 1 gives department counts and Table 2 reports scale scores by department. | Nursing students excluded from medical-student subset; no proportion-domain k/N impact. | Included in the systematic review but contributed no pooled outcome |
| 24. Almuhanna 2025  Attitudes and Awareness of Medical Students Toward Teleradiology and the Application of Artificial Intelligence in Diagnostic Radiology: A Cross-Sectional Study | Medical students from second to sixth academic years and interns, total n=292. | Y | Interns fall within the eligibility window (training through internship), so the medical-students-and-interns sample required no further disaggregation; P1 and P4 use n total=292. Source: Methods/Participants: 292 medical students and interns. | None; interns are within the eligible medical-training window and were retained. | Included in the systematic review and quantitative synthesis |
| 25. Sami 2025  Medical students’ attitudes toward AI in education: perception, effectiveness, and its credibility | MBBS students (n=595) and BDS/dental students (n=107), total n=702. | N | discipline counts are reported, but MBBS-only event counts for extracted domains were not found; reported proportions are combined MBBS+BDS. Source: Methods questionnaire collected discipline; Table 1 reports MBBS=595 and BDS=107; results present combined AI perception/effectiveness/credibility outcomes. | BDS students not separable from outcome numerators; strict exclusion would remove mixed total from P1/P3/P4/P6/P9. | Excluded because medical-student-specific outcomes were not separable |
| 38. Mousavi Baigi 2025  Medical and Paramedical Students’ Perspectives on Artificial Intelligence in a Low- and Middle-Income Country: A Cross-Sectional Study | Medical students (n=95) and paramedical students (n=113). | Y | Table 1 reports medical and paramedical columns separately; medical-student-specific values used where proportion domains contributed. Source: Table 1 demographics: separate medical-student and paramedical-student columns with totals 95 and 113. | Paramedical students excluded; P1/P3/P5 use n total=95. | Included in the systematic review and quantitative synthesis |
| 42. Farooq and Usmani 2025  Artificial Intelligence in Medical Education | MBBS students (n=92), BDS students (n=58), and allied health sciences students (n=2), total n=152. | N | academic discipline counts are reported, but the extracted P1/P2/P3/P4/P5/P8 outcomes were reported for the combined sample; MBBS-only event counts were not found. Source: Results/Table I: 92 MBBS, 58 BDS, and 2 allied health sciences students; AI attitude/knowledge summaries are presented for all 152 participants. | BDS and allied health students not separable from outcome numerators; strict exclusion would remove mixed total from P1/P2/P3/P4/P5/P8. | Excluded because medical-student-specific outcomes were not separable |
| 46. Naseer 2025  Navigating the integration of artificial intelligence in the medical education curriculum: a mixed‑methods study exploring the perspectives of medical students and faculty in Pakistan‑ | Medical students (n=153) and faculty members (n=83), total n=236. | Y | Participant type was reported and quantitative scores were compared between students and faculty. No numeric proportion-domain event/total values were contributed by this study. Source: Abstract/Methods: quantitative component included 153 medical students and 83 faculty; results compare student and faculty composite scores. | Faculty excluded from medical-student subset; no proportion-domain k/N impact. | Included in the systematic review but contributed no pooled outcome |
| 52. Allam 2024  Medical students and house officers’ perception, attitude and potential barriers towards artificial intelligence in Egypt, cross sectional survey | Medical students (n=999) and house officers (n=347), total n=1346. | Y | House officers fall within the eligibility window; this study contributed no proportion-domain event counts because P1-P5 were not extractable as medical-student-specific values (recorded NA in Table S9). | None; house officers are within the eligible medical-training window. | Included in the systematic review but contributed no pooled outcome |
| 77. Tangadulrat 2023  Using ChatGPT for Clinical Practice and Medical Education: Cross-Sectional Survey of Medical Students’ and Physicians’ Perceptions | Medical students (n=124), interns (n=46), residents (n=37), and attending staff (n=32). | Y | Role-specific results compare medical students with graduated physicians; medical-student-specific event counts and denominators were extractable. Source: Abstract/Results: participant groups listed; results report positive responses separately for medical students versus graduated physicians. | Interns, residents, and attending staff excluded; P1/P9 use n total=124. | Included in the systematic review and quantitative synthesis |
| 78. Oluwadiya 2023  Exploring Artificial Intelligence in the Nigerian Medical Educational Space: An Online Cross-sectional Study of Perceptions, Risks and Benefits among Students and Lecturers from Ten Universities | Clinical-year medical students (n=708) and lecturers (n=294) from ten medical schools; total responses n=1003. | Y | Respondent type was reported and student-versus-lecturer percentages were provided; student-specific values were used for extracted proportions. Source: Abstract/Results: survey administered to clinical-year medical students and lecturers; results report 708 students and 294 lecturers. | Lecturers and one unclassified/missing-role response excluded; P4/P8 use n total=708. | Included in the systematic review and quantitative synthesis |
| 86. Ahmed 2022  Knowledge, attitude, and practice of artificial intelligence among doctors and medical students in Pakistan: A cross-sectional online survey | Medical students (n=247) and doctors (n=223), total n=470. | Y | Occupation was reported and results give doctor-versus-student counts for key knowledge/awareness outcomes; student-specific denominators used. Source: Abstract/Results: 223 doctors and 247 medical students; selected outcomes reported separately for doctors and students. | Doctors excluded; P1/P5/P6 use n total=247. | Included in the systematic review and quantitative synthesis |
| 93. Boillat 2022  Readiness to Embrace Artificial Intelligence Among Medical Doctors and Students: Questionnaire-Based Study | Medical doctors (n=105) and medical students (n=102), total n=207. | Y | Group membership was reported and mean familiarity/training results were compared between doctors and students. No numeric proportion-domain event/total values were contributed by this study. Source: Abstract/Methods/Results: 105 medical doctors and 102 medical students; results compare familiarity and training between groups. | Medical doctors excluded from student subset; no proportion-domain k/N impact. | Included in the systematic review but contributed no pooled outcome |
| 95. Bisdas 2021  Artificial Intelligence in Medicine: A Multinational Multi-Center Survey on the Medical and Dental Students’ Perception | Medical students (n=2495) and dental students (n=638), total n=3133. | Y for participant count; N for proportion outcomes | Table 1 reports medical-student share, but medical-student-only event counts for perception items were not extracted as numeric proportion domains. Source: Results/Table 1: participant characteristics include 2495 medical students among 3133 respondents; title and methods identify medical and dental students. | Dental students (no separable medical-student proportion extracted) | Included in the systematic review but contributed no pooled outcome |
| 96. Wood 2021  Are We Ready to Integrate Artificial Intelligence Literacy into Medical School Curriculum: Students and Faculty Survey | Medical students (121 completed; 117 usable) and clinical faculty (52 completed; 44 usable). | Y | Student and faculty results were reported separately; usable medical-student responses were used where proportion domains contributed. Source: Methods/Results: survey distributed to students and faculty; missing data left 117 usable student and 44 usable faculty responses. | Clinical faculty excluded; P5/P7 use n total=117. | Included in the systematic review and quantitative synthesis |

**Table S6. Characteristics of 96 included cross-sectional studies of medical students’ attitudes toward artificial intelligence in healthcare (2019-2026; 37 countries).**

| **Study #** | **Study ID** | **First author** | **Year** | **Country/region** | **Medical-student n** | **AI type** | **WHO region** | **World Bank income** | **JBI risk** | **Training stage** |
| --- | --- | --- | --- | --- | --- | --- | --- | --- | --- | --- |
| 1 | Cao 2026 a | Xi Cao | 2026 | China | 1062 | ChatGPT/LLM or generative AI | Western Pacific | Upper-middle income | Moderate | Mixed undergraduate/postgraduate |
| 2 | Kara 2026 a | Ali Veysel Kara | 2026 | Türkiye | 1346 | ChatGPT/LLM or generative AI | Europe | Upper-middle income | Moderate | Mixed (prep-year 6) |
| 3 | Jamjoom 2026 a | Jude Jamjoom | 2026 | Saudi Arabia | 117 | Broad/general AI | Eastern Mediterranean | High income | Moderate | Mixed undergraduate years |
| 4 | Xu 2026 a | Rixiang Xu | 2026 | China | 661 | Broad/general AI | Western Pacific | Upper-middle income | Low | Fourth-year clinical students |
| 5 | Albusmait 2026 a | Amna Albusmait | 2026 | Bahrain | 350 | Broad/general AI | Eastern Mediterranean | High income | Moderate | Mixed (years 1-6) |
| 6 | Xu 2026 b | Alan Yuesheng Xu | 2026 | United States | 177 | ChatGPT/LLM or generative AI | Americas | High income | Moderate | Undergraduate medical students |
| 7 | Prasad 2026 a | Sumit Prasad | 2026 | India | 317 | Broad/general AI | South-East Asia | Low/lower-middle income | Moderate | Mixed undergraduate + postgraduate |
| 8 | Yousef 2026 a | Yacoub A. Yousef | 2026 | Jordan | 353 | Domain-specific AI | Eastern Mediterranean | Low/lower-middle income | Moderate | Medical student subgroup from mixed sample |
| 9 | Catan-Inan 2026 a | Funda Catan-Inan | 2026 | Türkiye | 183 | Broad/general AI | Europe | Upper-middle income | Low | Mixed undergraduate years |
| 10 | Hu 2025 a | Na Hu | 2025 | China | 1133 | ChatGPT/LLM or generative AI | Western Pacific | Upper-middle income | Low | Mixed medical student levels |
| 11 | Ghanem 2025 a | Omar A. Ghanem | 2025 | Egypt | 423 | ChatGPT/LLM or generative AI | Eastern Mediterranean | Low/lower-middle income | Low | Mixed undergraduate years |
| 12 | Ahmed 2025 a | Weam Mohamed Meargni Ahmed | 2025 | Sudan | 1443 | ChatGPT/LLM or generative AI | Eastern Mediterranean | Low/lower-middle income | Low | Mixed undergraduate years |
| 13 | Chakri 2025 a | Imad Chakri | 2025 | Morocco | 580 | Broad/general AI | Eastern Mediterranean | Low/lower-middle income | Low | Mixed undergraduate years (1-7) |
| 14 | Giavina-Bianchi 2025 a | Bruna Giavina-Bianchi | 2025 | Brazil | 145 | Broad/general AI | Americas | Upper-middle income | Moderate | Mixed undergraduate years (1-6) |
| 15 | Fussholler 2025 a | Anna Fußhöller | 2025 | Germany | 84 | ChatGPT/LLM or generative AI | Europe | High income | Moderate | Mixed undergraduate years (preclinical + clinical) |
| 16 | Tajima 2025 a | Hiroshi Tajima | 2025 | Japan | 118 | ChatGPT/LLM or generative AI | Western Pacific | High income | Moderate | First-year medical students |
| 17 | Elhassan 2025 a | Safia Elwaleed Elhassan | 2025 | Saudi Arabia | 293 | ChatGPT/LLM or generative AI | Eastern Mediterranean | High income | Moderate | Mixed undergraduate years (1-6) |
| 18 | Tran 2025 a | Cecilia Tran | 2025 | Canada | 166 | ChatGPT/LLM or generative AI | Americas | High income | Moderate | Mixed undergraduate years (1-4) |
| 19 | Almehmadi 2025 a | Bayan Almehmadi | 2025 | Saudi Arabia | 170 | ChatGPT/LLM or generative AI | Eastern Mediterranean | High income | Low | Mixed undergraduate years (1-6) |
| 20 | Arab-Borzu 2025 a | Zahra Arab-Borzu | 2025 | Iran | 280 | Broad/general AI | Eastern Mediterranean | Low/lower-middle income | Low | Mixed preclinical and clinical students |
| 21 | Onetiu 2025 a | Florina Onetiu | 2025 | Romania | 161 | Broad/general AI | Europe | High income | Low | Mixed undergraduate years (1-6) |
| 22 | Alfahl 2025 a | Samah Alfahl | 2025 | Saudi Arabia | 189 | Broad/general AI | Eastern Mediterranean | High income | Moderate | Mixed undergraduate years |
| 23 | Zhang 2025 a | Min Zhang | 2025 | China | 254 | Broad/general AI | Western Pacific | Upper-middle income | Low | Third-year medical undergraduates |
| 24 | Almuhanna 2025 a | Afnan Almuhanna | 2025 | Saudi Arabia | 292 | Domain-specific AI | Eastern Mediterranean | High income | Moderate | Medical students and interns |
| 26 | Abdelhafiz 2025 a | Ahmed Samir Abdelhafiz | 2025 | Egypt | 614 | ChatGPT/LLM or generative AI | Eastern Mediterranean | Low/lower-middle income | Moderate | Undergraduate medical students |
| 27 | Liu 2026 a | Mingchan Liu | 2026 | China | 788 | Broad/general AI | Western Pacific | Upper-middle income | Moderate | Mixed undergraduate years (1-5) |
| 28 | Duan 2025 a | Shuo Duan | 2025 | China | 553 | ChatGPT/LLM or generative AI | Western Pacific | Upper-middle income | Moderate | Mixed undergraduate and postgraduate medical students |
| 29 | Hanifa 2025 a | Hamdah Hanifa | 2025 | Syria | 564 | Broad/general AI | Eastern Mediterranean | Low/lower-middle income | Moderate | Mixed preclinical and clinical years |
| 30 | Fernando 2025 a | Poorna Fernando | 2025 | Sri Lanka | 304 | Broad/general AI | South-East Asia | Low/lower-middle income | Moderate | Mixed undergraduate years (1-final) |
| 31 | Gualda-Gea 2025 a | Juan José Gualda-Gea | 2025 | Spain | 29 | ChatGPT/LLM or generative AI | Europe | High income | Moderate | Fifth-year medical students |
| 32 | Ignjatovic 2025 a | Aleksandra Ignjatović | 2025 | Serbia | 1212 | ChatGPT/LLM or generative AI | Europe | Upper-middle income | Moderate | Undergraduate medical students |
| 33 | Sanri 2025 a | Emre Sanri | 2025 | Türkiye | 120 | Broad/general AI | Europe | Upper-middle income | Moderate | First- to fourth-year medical students |
| 34 | Shah 2025 a | M Hassaan Shah | 2025 | Pakistan | 200 | Broad/general AI | Eastern Mediterranean | Low/lower-middle income | Moderate | Mixed undergraduate medical years |
| 35 | Gopidesi 2025 a | Madhulatha Gopidesi | 2025 | India | 210 | Broad/general AI | South-East Asia | Low/lower-middle income | Moderate | MBBS phase II-IV (undergraduate) |
| 36 | Ringeval 2025 a | Mickaël Ringeval | 2025 | Canada | 361 | Broad/general AI | Americas | High income | Low | Mixed medical students (prepandemic and postpandemic cohorts) |
| 37 | Ejas 2025 a | Faiza Ejas | 2025 | Ecuador; Ethiopia; India; Mauritius; Pakistan; United Arab Emirates; United States | 1180 | ChatGPT/LLM or generative AI | Multiple regions | Mixed income | Moderate | Mixed medical students across years 1-7 |
| 38 | Mousavi Baigi 2025 a | Seyyedeh Fatemeh Mousavi Baigi | 2025 | Iran | 95 | Broad/general AI | Eastern Mediterranean | Low/lower-middle income | Moderate | Medical student subgroup from mixed sample |
| 39 | Falcon 2025 a | Robbi Miguel G. Falcon | 2025 | Philippines | 20 | ChatGPT/LLM or generative AI | Western Pacific | Low/lower-middle income | Moderate | Second-year preclinical medical students |
| 40 | Rezazadeh 2025 a | Hossein Rezazadeh | 2025 | Iran | 340 | Broad/general AI | Eastern Mediterranean | Low/lower-middle income | Moderate | Mixed undergraduate years (1-5) |
| 41 | Sunmboye 2025 a | Kehinde Sunmboye | 2025 | United Kingdom | 230 (200 analyzed) | Broad/general AI | Europe | High income | Low | Undergraduate medical students |
| 43 | AlZaabi 2025 a | Adhari AlZaabi | 2025 | Oman | 84 | Broad/general AI | Eastern Mediterranean | High income | Moderate | Students entering clinical years after preclinical training |
| 44 | Ziapour 2025 a | Arash Ziapour | 2025 | Iran | 800 | Broad/general AI | Eastern Mediterranean | Low/lower-middle income | Moderate | Mixed undergraduate years (1-5) |
| 45 | Alharkan 2025 a | Khalid S. Alharkan | 2025 | Saudi Arabia | 525 | Broad/general AI | Eastern Mediterranean | High income | Moderate | Mixed undergraduate years (2-6) |
| 46 | Naseer 2025 a | Muhammad Ahsan Naseer | 2025 | Pakistan | 153 | Broad/general AI | Eastern Mediterranean | Low/lower-middle income | Moderate | Mixed undergraduate years (2-4) |
| 47 | Gonzalez-Bravo 2025 a | Adrián E. González-Bravo | 2025 | Puerto Rico | 194 | ChatGPT/LLM or generative AI | Americas | High income | Moderate | First- and second-year students |
| 48 | Almarzouki 2025 a | Abeer F. Almarzouki | 2025 | Saudi Arabia | 354 | Broad/general AI | Eastern Mediterranean | High income | Moderate | Mixed undergraduate years (basic + clinical) |
| 49 | Dhurandhar 2025 a | Diwakar Dhurandhar | 2025 | India | 482 | Broad/general AI | South-East Asia | Low/lower-middle income | Moderate | Mixed undergraduate years + internship |
| 50 | Allam 2024 b | Ahmed Hafez Allam | 2024 | Algeria; Egypt; Iraq; Jordan; Libya; Palestine; Sudan; Syria; Yemen | 4492 | Domain-specific AI | Eastern Mediterranean | Mixed income | Moderate | Mixed undergraduate years |
| 51 | Jebreen 2024 a | Kamel Jebreen | 2024 | Palestine | 349 | Broad/general AI | Eastern Mediterranean | Low/lower-middle income | Moderate | Mixed undergraduate years |
| 52 | Allam 2024 c | Rasha Mahmoud Allam | 2024 | Egypt | 999 | Broad/general AI | Eastern Mediterranean | Low/lower-middle income | Moderate | Medical students + house officers |
| 53 | Tao 2024 a | Wenjuan Tao | 2024 | China | 693 | ChatGPT/LLM or generative AI | Western Pacific | Upper-middle income | Moderate | Mixed medical students |
| 54 | Ganjavi 2024 a | Conner Ganjavi | 2024 | United States | 415 | ChatGPT/LLM or generative AI | Americas | High income | Moderate | Mixed U.S. medical students |
| 55 | Alwadani 2024 a | Fahad Abdullah Saeed Alwadani | 2024 | Saudi Arabia | 303 | Broad/general AI | Eastern Mediterranean | High income | Moderate | Clinical-year students + interns |
| 56 | Abu Hammour 2024 a | Adnan Abu Hammour | 2024 | Jordan | 244 | ChatGPT/LLM or generative AI | Eastern Mediterranean | Upper-middle income | Moderate | Senior clinical students (year 4+) |
| 57 | Faroog 2024 a | Zainudheen Faroog | 2024 | Saudi Arabia | 345 | Domain-specific AI | Eastern Mediterranean | High income | Moderate | Mixed undergraduate years |
| 58 | Laupichler 2024 a | Matthias Carl Laupichler | 2024 | Germany | 377 | Broad/general AI | Europe | High income | Moderate | Mixed medical student semesters |
| 59 | Amin 2024 a | Mohammed Hammad Jaber Amin | 2024 | Sudan | 762 | Broad/general AI | Eastern Mediterranean | Low/lower-middle income | Moderate | Mixed undergraduate years |
| 60 | Jackson 2024 a | Preetha Jackson | 2024 | India | 325 | Broad/general AI | South-East Asia | Low/lower-middle income | Moderate | Mixed undergraduate years |
| 61 | Angkurawaranon 2024 a | Salita Angkurawaranon | 2024 | Thailand | 352 | Domain-specific AI | South-East Asia | Upper-middle income | Moderate | Mixed undergraduate years (1-6) |
| 62 | Lugito 2024 a | Nata Pratama Hardjo Lugito | 2024 | Indonesia | 650 | Broad/general AI | South-East Asia | Upper-middle income | Moderate | Mixed pre-clinical and clinical students |
| 63 | Daher 2024 a | Omar A. Daher | 2024 | Lebanon | 365 | Domain-specific AI | Eastern Mediterranean | Low/lower-middle income | Moderate | Mixed undergraduate years |
| 64 | Weidener 2024 a | Lukas Weidener | 2024 | Germany; Austria; Switzerland | 487 | Broad/general AI | Europe | High income | Moderate | Mixed undergraduate years incl practical year |
| 65 | Luong 2025 a | Jason Luong | 2025 | Global Survey - Africa, Asia, Europe, and North America | 223 | Broad/general AI | Multiple regions | Mixed income | Moderate | Mixed medical school years |
| 66 | Al Shahrani 2024 a | Abeer Al Shahrani | 2024 | Saudi Arabia | 572 | Broad/general AI | Eastern Mediterranean | High income | Moderate | Mixed undergraduate years |
| 67 | Malesevic 2024 a | Anamaria Malešević | 2024 | Croatia; Slovakia | 1701 | Broad/general AI | Europe | High income | Moderate | Mixed undergraduate years |
| 68 | Hamad 2024 a | Mohammad Hamad | 2024 | Jordan | 858 | Broad/general AI | Eastern Mediterranean | Upper-middle income | Moderate | Mixed undergraduate years |
| 69 | Alammari 2024 a | Dalia M. Alammari | 2024 | Saudi Arabia | 207 | Broad/general AI | Eastern Mediterranean | High income | Moderate | Senior medical students + interns |
| 70 | Gandhi 2024 a | Rohankumar Gandhi | 2024 | India | 605 | Broad/general AI | South-East Asia | Low/lower-middle income | Low | Mixed undergraduate and postgraduate |
| 71 | Ozbek Guven 2024 a | Gamze Özbek Güven | 2024 | Türkiye | 542 | Broad/general AI | Europe | Upper-middle income | Moderate | Mixed undergraduate years |
| 72 | Xu 2024 a | Alan Y. Xu | 2024 | United States | 102 | ChatGPT/LLM or generative AI | Americas | High income | Low | Mixed medical school years |
| 73 | Sorte 2024 a | Smita R. Sorte | 2024 | India | 217 | Broad/general AI | South-East Asia | Low/lower-middle income | Moderate | Mixed undergraduate years |
| 74 | Al-Roomi 2024 a | Khaldoon Al-Roomi | 2024 | Bahrain | 152 | Broad/general AI | Eastern Mediterranean | High income | Moderate | Mixed premedical/preclinical/clinical years |
| 75 | Liu 2024 a | Fuze Liu | 2024 | China | 311 | ChatGPT/LLM or generative AI | Western Pacific | Upper-middle income | Low | Mixed undergraduate/master/doctoral clinical medicine students |
| 76 | Alkhaaldi 2023 a | Saif M I Alkhaaldi | 2023 | United Arab Emirates | 265 | ChatGPT/LLM or generative AI | Eastern Mediterranean | High income | Low | Graduating medical students / residency applicants |
| 77 | Tangadulrat 2023 a | Pasin Tangadulrat | 2023 | Thailand | 124 | ChatGPT/LLM or generative AI | South-East Asia | Upper-middle income | Moderate | Final-year medical students (subgroup from mixed cohort) |
| 78 | Oluwadiya 2023 a | Kehinde Sunday Oluwadiya | 2023 | Nigeria | 708 | ChatGPT/LLM or generative AI | Africa | Low/lower-middle income | Moderate | Clinical-year medical students (subgroup from mixed cohort) |
| 79 | Tung 2023 a | Alvin Yong Zong Tung | 2023 | Malaysia | 301 | Broad/general AI | Western Pacific | Upper-middle income | Moderate | Mixed undergraduate years |
| 80 | Stewart 2023 a | Jonathon Stewart | 2023 | Australia | 134 | Broad/general AI | Western Pacific | High income | Moderate | Mixed medical school years |
| 81 | Al Hadithy 2023 a | Zinah A. Al Hadithy | 2023 | Oman | 221 | Broad/general AI | Eastern Mediterranean | High income | Moderate | Clinical-year medical students |
| 82 | Buabbas 2023 a | Ali Jasem Buabbas | 2023 | Kuwait | 352 | Broad/general AI | Eastern Mediterranean | High income | Low | Mixed medical students |
| 83 | Barreiro-Ares 2023 a | Andrés Barreiro-Ares | 2023 | Spain | 281 | Domain-specific AI | Europe | High income | Moderate | Mixed medical students |
| 84 | Biri 2023 a | Sairavi Kiran Biri | 2023 | India | 172 | ChatGPT/LLM or generative AI | South-East Asia | Low/lower-middle income | Moderate | Mixed undergraduate years |
| 85 | Doumat 2022 a | George Doumat | 2022 | Lebanon | 206 | Broad/general AI | Eastern Mediterranean | Low/lower-middle income | Moderate | Mixed medical students |
| 86 | Ahmed 2022 a | Zaboor Ahmed | 2022 | Pakistan | 247 | Broad/general AI | Eastern Mediterranean | Low/lower-middle income | Moderate | Mixed doctors/medical students |
| 87 | Liu 2022 a | David Shalom Liu | 2022 | United States | 390 | Broad/general AI | Americas | High income | Moderate | Mixed medical students |
| 88 | Caparros Galan and Sendra Portero 2022 a | G. Caparrós Galán | 2022 | Spain | 341 | Domain-specific AI | Europe | High income | Moderate | Mixed medical students |
| 89 | Gillissen 2022 a | Adrian Gillissen | 2022 | Germany | 1053 | Broad/general AI | Europe | High income | Moderate | Mixed undergraduate/graduate |
| 90 | Pucchio 2022 a | Aidan Pucchio | 2022 | Canada | 486 | Broad/general AI | Americas | High income | Moderate | Mixed undergraduate years |
| 91 | McLennan 2022 a | Stuart McLennan | 2022 | Germany | 844 | Broad/general AI | Europe | High income | Moderate | First-year medical students |
| 92 | Blease 2022 a | Charlotte Blease | 2022 | Ireland; Northern Ireland | 252 | Broad/general AI | Europe | High income | Moderate | Final-year medical students |
| 93 | Boillat 2022 a | Thomas Boillat | 2022 | Multinational / Global | 102 | Broad/general AI | Multiple regions | Mixed income | Moderate | Medical student subgroup from mixed sample |
| 94 | Blacketer 2021 a | Charlotte Blacketer | 2021 | Australia; New Zealand; United States | 245 | Broad/general AI | Multiple regions | High income | Moderate | Senior medical students (penultimate/final year) |
| 95 | Bisdas 2021 a | Sotirios Bisdas | 2021 | 63 countries across all continents | 2495 | Broad/general AI | Multiple regions | Mixed income | Moderate | Mixed medical and dental students |
| 96 | Wood 2021 a | Elena A Wood | 2021 | United States | 117 | Broad/general AI | Americas | High income | Moderate | Mixed medical school years |
| 97 | Sit 2020 a | Cherry Sit | 2020 | United Kingdom | 484 | Domain-specific AI | Europe | High income | Moderate | Mixed medical school years |
| 98 | Pinto dos Santos 2019 a | D. Pinto dos Santos | 2019 | Germany | 263 | Domain-specific AI | Europe | High income | Moderate | Mixed undergraduate medical students |

*AI: artificial intelligence; JBI: Joanna Briggs Institute; LLM: large language model.*

**Table S7. Joanna Briggs Institute Critical Appraisal Checklist for Analytical Cross-Sectional Studies: item-level ratings for all 96 included studies.**

| **#** | **Study ID** | **Author** | **Year** | **Q1** | **Q2** | **Q3** | **Q4** | **Q5** | **Q6** | **Q7** | **Q8** | **Overall** | **Score** |
| --- | --- | --- | --- | --- | --- | --- | --- | --- | --- | --- | --- | --- | --- |
| 1 | Cao 2026 a | Xi Cao | 2026 | Yes | Yes | Yes | No | No | No | Yes | Yes | Moderate | 5.0 |
| 2 | Kara 2026 a | Ali Veysel Kara | 2026 | Yes | Yes | Yes | No | No | No | Yes | Yes | Moderate | 5.0 |
| 3 | Jamjoom 2026 a | Jude Jamjoom | 2026 | Yes | Yes | Yes | No | No | No | Yes | Yes | Moderate | 5.0 |
| 4 | Xu 2026 a | Rixiang Xu | 2026 | Yes | Yes | Unclear | No | Yes | Yes | Yes | Yes | Low | 6.0 |
| 5 | Albusmait 2026 a | Amna Albusmait | 2026 | Yes | Yes | Yes | No | No | No | Yes | Yes | Moderate | 5.0 |
| 6 | Xu 2026 b | Alan Yuesheng Xu | 2026 | Yes | Yes | Unclear | No | No | No | Yes | Yes | Moderate | 4.0 |
| 7 | Prasad 2026 a | Sumit Prasad | 2026 | Yes | Yes | Yes | No | No | No | Yes | Yes | Moderate | 5.0 |
| 8 | Yousef 2026 a | Yacoub A. Yousef | 2026 | Yes | Yes | Yes | No | No | No | Yes | Yes | Moderate | 5.0 |
| 9 | Catan-Inan 2026 a | Funda Catan-Inan | 2026 | Yes | Yes | Yes | No | Yes | Yes | Yes | Yes | Low | 7.0 |
| 10 | Hu 2025 a | Na Hu | 2025 | Yes | Yes | Yes | No | Yes | Yes | Yes | Yes | Low | 7.0 |
| 11 | Ghanem 2025 a | Omar A. Ghanem | 2025 | Yes | Yes | Yes | No | Yes | Yes | Yes | Yes | Low | 7.0 |
| 12 | Ahmed 2025 a | Weam Mohamed Meargni Ahmed | 2025 | Yes | Yes | Yes | No | Yes | Yes | Yes | Yes | Low | 7.0 |
| 13 | Chakri 2025 a | Imad Chakri | 2025 | Yes | Yes | Yes | No | Yes | No | Yes | Yes | Low | 6.0 |
| 14 | Giavina-Bianchi 2025 a | Bruna Giavina-Bianchi | 2025 | Yes | Yes | Unclear | No | No | No | Yes | Yes | Moderate | 4.0 |
| 15 | Fussholler 2025 a | Anna Fußhöller | 2025 | Yes | Yes | Unclear | No | No | No | Yes | Yes | Moderate | 4.0 |
| 16 | Tajima 2025 a | Hiroshi Tajima | 2025 | Yes | Yes | Yes | No | No | No | Yes | Yes | Moderate | 5.0 |
| 17 | Elhassan 2025 a | Safia Elwaleed Elhassan | 2025 | Yes | Yes | Yes | No | No | No | Yes | Yes | Moderate | 5.0 |
| 18 | Tran 2025 a | Cecilia Tran | 2025 | Yes | Yes | Yes | No | No | No | Yes | Yes | Moderate | 5.0 |
| 19 | Almehmadi 2025 a | Bayan Almehmadi | 2025 | Yes | Yes | Yes | No | Yes | Yes | Yes | Yes | Low | 7.0 |
| 20 | Arab-Borzu 2025 a | Zahra Arab-Borzu | 2025 | Yes | Yes | Yes | No | Yes | Yes | Yes | Yes | Low | 7.0 |
| 21 | Onetiu 2025 a | Florina Onetiu | 2025 | Yes | Yes | Yes | No | Yes | Yes | Yes | Yes | Low | 7.0 |
| 22 | Alfahl 2025 a | Samah Alfahl | 2025 | Yes | Yes | Yes | No | No | No | Yes | Yes | Moderate | 5.0 |
| 23 | Zhang 2025 a | Min Zhang | 2025 | Yes | Yes | Yes | No | Yes | Yes | Yes | Yes | Low | 7.0 |
| 24 | Almuhanna 2025 a | Afnan Almuhanna | 2025 | Yes | Yes | Unclear | No | No | No | Yes | Yes | Moderate | 4.0 |
| 26 | Abdelhafiz 2025 a | Ahmed Samir Abdelhafiz | 2025 | Yes | Yes | Yes | No | No | No | Yes | Yes | Moderate | 5.0 |
| 27 | Liu 2026 a | Mingchan Liu | 2026 | Yes | Yes | Yes | No | No | No | Yes | Yes | Moderate | 5.0 |
| 28 | Duan 2025 a | Shuo Duan | 2025 | Yes | Yes | Yes | No | No | No | Yes | Yes | Moderate | 5.0 |
| 29 | Hanifa 2025 a | Hamdah Hanifa | 2025 | Yes | Yes | Yes | No | No | No | Yes | Yes | Moderate | 5.0 |
| 30 | Fernando 2025 a | Poorna Fernando | 2025 | Yes | Yes | Yes | No | No | No | Yes | Yes | Moderate | 5.0 |
| 31 | Gualda-Gea 2025 a | Juan José Gualda-Gea | 2025 | Yes | Yes | Unclear | No | No | No | Yes | Yes | Moderate | 4.0 |
| 32 | Ignjatovic 2025 a | Aleksandra Ignjatović | 2025 | Yes | Yes | Yes | No | No | No | Yes | Yes | Moderate | 5.0 |
| 33 | Sanri 2025 a | Emre Sanri | 2025 | Yes | Yes | Yes | No | No | No | Yes | Yes | Moderate | 5.0 |
| 34 | Shah 2025 a | M Hassaan Shah | 2025 | Yes | Yes | Yes | No | No | No | Yes | Yes | Moderate | 5.0 |
| 35 | Gopidesi 2025 a | Madhulatha Gopidesi | 2025 | Yes | Yes | Yes | No | No | No | Yes | Yes | Moderate | 5.0 |
| 36 | Ringeval 2025 a | Mickaël Ringeval | 2025 | Yes | Yes | Yes | No | Yes | Yes | Yes | Yes | Low | 7.0 |
| 37 | Ejas 2025 a | Faiza Ejas | 2025 | Yes | Yes | Yes | No | No | No | Yes | Yes | Moderate | 5.0 |
| 38 | Mousavi Baigi 2025 a | Seyyedeh Fatemeh Mousavi Baigi | 2025 | Yes | Yes | Yes | No | No | No | Yes | Yes | Moderate | 5.0 |
| 39 | Falcon 2025 a | Robbi Miguel G. Falcon | 2025 | Yes | Yes | Yes | No | No | No | Yes | Yes | Moderate | 5.0 |
| 40 | Rezazadeh 2025 a | Hossein Rezazadeh | 2025 | Yes | Yes | Yes | No | No | No | Yes | Yes | Moderate | 5.0 |
| 41 | Sunmboye 2025 a | Kehinde Sunmboye | 2025 | Yes | Yes | Yes | No | Yes | Yes | Yes | Yes | Low | 7.0 |
| 43 | AlZaabi 2025 a | Adhari AlZaabi | 2025 | Yes | Yes | Yes | No | No | No | Yes | Yes | Moderate | 5.0 |
| 44 | Ziapour 2025 a | Arash Ziapour | 2025 | Yes | Yes | Yes | No | No | No | Yes | Yes | Moderate | 5.0 |
| 45 | Alharkan 2025 a | Khalid S. Alharkan | 2025 | Yes | Yes | Yes | No | No | No | Yes | Yes | Moderate | 5.0 |
| 46 | Naseer 2025 a | Muhammad Ahsan Naseer | 2025 | Yes | Yes | Yes | No | No | No | Yes | Yes | Moderate | 5.0 |
| 47 | Gonzalez-Bravo 2025 a | Adrián E. González-Bravo | 2025 | Yes | Yes | Yes | No | No | No | Yes | Yes | Moderate | 5.0 |
| 48 | Almarzouki 2025 a | Abeer F. Almarzouki | 2025 | Yes | Yes | Yes | No | No | No | Yes | Yes | Moderate | 5.0 |
| 49 | Dhurandhar 2025 a | Diwakar Dhurandhar | 2025 | Yes | Yes | Yes | No | No | No | Yes | Yes | Moderate | 5.0 |
| 50 | Allam 2024 b | Ahmed Hafez Allam | 2024 | Yes | Yes | Yes | No | No | No | Yes | Yes | Moderate | 5.0 |
| 51 | Jebreen 2024 a | Kamel Jebreen | 2024 | Yes | Yes | Yes | No | No | No | Yes | Yes | Moderate | 5.0 |
| 52 | Allam 2024 c | Rasha Mahmoud Allam | 2024 | Yes | Yes | Yes | No | No | No | Yes | Yes | Moderate | 5.0 |
| 53 | Tao 2024 a | Wenjuan Tao | 2024 | Yes | Yes | Yes | No | No | No | Yes | Yes | Moderate | 5.0 |
| 54 | Ganjavi 2024 a | Conner Ganjavi | 2024 | Yes | Yes | Yes | No | No | No | Yes | Yes | Moderate | 5.0 |
| 55 | Alwadani 2024 a | Fahad Abdullah Saeed Alwadani | 2024 | Yes | Yes | Yes | No | No | No | Yes | Yes | Moderate | 5.0 |
| 56 | Abu Hammour 2024 a | Adnan Abu Hammour | 2024 | Yes | Yes | Yes | No | No | No | Yes | Yes | Moderate | 5.0 |
| 57 | Faroog 2024 a | Zainudheen Faroog | 2024 | Yes | Yes | Yes | No | No | No | Yes | Yes | Moderate | 5.0 |
| 58 | Laupichler 2024 a | Matthias Carl Laupichler | 2024 | Yes | Yes | Yes | No | No | No | Yes | Yes | Moderate | 5.0 |
| 59 | Amin 2024 a | Mohammed Hammad Jaber Amin | 2024 | Yes | Yes | Yes | No | No | No | Yes | Yes | Moderate | 5.0 |
| 60 | Jackson 2024 a | Preetha Jackson | 2024 | Yes | Yes | Yes | No | No | No | Yes | Yes | Moderate | 5.0 |
| 61 | Angkurawaranon 2024 a | Salita Angkurawaranon | 2024 | Yes | Yes | Yes | No | No | No | Yes | Yes | Moderate | 5.0 |
| 62 | Lugito 2024 a | Nata Pratama Hardjo Lugito | 2024 | Yes | Yes | Yes | No | No | No | Yes | Yes | Moderate | 5.0 |
| 63 | Daher 2024 a | Omar A. Daher | 2024 | Yes | Yes | Yes | No | No | No | Yes | Yes | Moderate | 5.0 |
| 64 | Weidener 2024 a | Lukas Weidener | 2024 | Yes | Yes | Yes | No | No | No | Yes | Yes | Moderate | 5.0 |
| 65 | Luong 2025 a | Jason Luong | 2025 | Yes | Yes | Yes | No | No | No | Yes | Yes | Moderate | 5.0 |
| 66 | Al Shahrani 2024 a | Abeer Al Shahrani | 2024 | Yes | Yes | Yes | No | No | No | Yes | Yes | Moderate | 5.0 |
| 67 | Malesevic 2024 a | Anamaria Malešević | 2024 | Yes | Yes | Yes | No | No | No | Yes | Yes | Moderate | 5.0 |
| 68 | Hamad 2024 a | Mohammad Hamad | 2024 | Yes | Yes | Yes | No | No | No | Yes | Yes | Moderate | 5.0 |
| 69 | Alammari 2024 a | Dalia M. Alammari | 2024 | Yes | Yes | Unclear | No | No | No | Yes | Yes | Moderate | 4.0 |
| 70 | Gandhi 2024 a | Rohankumar Gandhi | 2024 | Yes | Yes | Yes | No | Yes | Yes | Yes | Yes | Low | 7.0 |
| 71 | Ozbek Guven 2024 a | Gamze Özbek Güven | 2024 | Yes | Yes | Yes | No | No | No | Yes | Yes | Moderate | 5.0 |
| 72 | Xu 2024 a | Alan Y. Xu | 2024 | Yes | Yes | Unclear | No | Yes | Yes | Yes | Yes | Low | 6.0 |
| 73 | Sorte 2024 a | Smita R. Sorte | 2024 | Yes | Yes | Yes | No | No | No | Yes | Yes | Moderate | 5.0 |
| 74 | Al-Roomi 2024 a | Khaldoon Al-Roomi | 2024 | Yes | Yes | Unclear | No | No | No | Yes | Yes | Moderate | 4.0 |
| 75 | Liu 2024 a | Fuze Liu | 2024 | Yes | Yes | Yes | No | Yes | Yes | Yes | Yes | Low | 7.0 |
| 76 | Alkhaaldi 2023 a | Saif M I Alkhaaldi | 2023 | Yes | Yes | Yes | No | Yes | Yes | Yes | Yes | Low | 7.0 |
| 77 | Tangadulrat 2023 a | Pasin Tangadulrat | 2023 | Yes | Yes | Yes | No | No | No | Yes | Yes | Moderate | 5.0 |
| 78 | Oluwadiya 2023 a | Kehinde Sunday Oluwadiya | 2023 | Yes | Yes | Yes | No | No | No | Yes | Yes | Moderate | 5.0 |
| 79 | Tung 2023 a | Alvin Yong Zong Tung | 2023 | Yes | Yes | Yes | No | No | No | Yes | Yes | Moderate | 5.0 |
| 80 | Stewart 2023 a | Jonathon Stewart | 2023 | Yes | Yes | Yes | No | No | No | Yes | Yes | Moderate | 5.0 |
| 81 | Al Hadithy 2023 a | Zinah A. Al Hadithy | 2023 | Yes | Yes | Yes | No | No | No | Yes | Yes | Moderate | 5.0 |
| 82 | Buabbas 2023 a | Ali Jasem Buabbas | 2023 | Yes | Yes | Yes | No | Yes | No | Yes | Yes | Low | 6.0 |
| 83 | Barreiro-Ares 2023 a | Andrés Barreiro-Ares | 2023 | Yes | Yes | Unclear | No | No | No | Yes | Yes | Moderate | 4.0 |
| 84 | Biri 2023 a | Sairavi Kiran Biri | 2023 | Yes | Yes | Yes | No | No | No | Yes | Yes | Moderate | 5.0 |
| 85 | Doumat 2022 a | George Doumat | 2022 | Yes | Yes | Unclear | No | No | No | Yes | Yes | Moderate | 4.0 |
| 86 | Ahmed 2022 a | Zaboor Ahmed | 2022 | Yes | Yes | Yes | No | No | No | Yes | Yes | Moderate | 5.0 |
| 87 | Liu 2022 a | David Shalom Liu | 2022 | Yes | Yes | Yes | No | No | No | Yes | Yes | Moderate | 5.0 |
| 88 | Caparros Galan and Sendra Portero 2022 a | G. Caparrós Galán | 2022 | Yes | Yes | Unclear | No | No | No | Yes | Yes | Moderate | 4.0 |
| 89 | Gillissen 2022 a | Adrian Gillissen | 2022 | Yes | Yes | Unclear | No | No | No | Yes | Yes | Moderate | 4.0 |
| 90 | Pucchio 2022 a | Aidan Pucchio | 2022 | Yes | Yes | Yes | No | No | No | Yes | Yes | Moderate | 5.0 |
| 91 | McLennan 2022 a | Stuart McLennan | 2022 | Yes | Yes | Unclear | No | No | No | Yes | Yes | Moderate | 4.0 |
| 92 | Blease 2022 a | Charlotte Blease | 2022 | Yes | Yes | Yes | No | No | No | Yes | Yes | Moderate | 5.0 |
| 93 | Boillat 2022 a | Thomas Boillat | 2022 | Yes | Yes | Yes | No | No | No | Yes | Yes | Moderate | 5.0 |
| 94 | Blacketer 2021 a | Charlotte Blacketer | 2021 | Yes | Yes | Yes | No | No | No | Yes | Yes | Moderate | 5.0 |
| 95 | Bisdas 2021 a | Sotirios Bisdas | 2021 | Yes | Yes | Unclear | No | No | No | Yes | Yes | Moderate | 4.0 |
| 96 | Wood 2021 a | Elena A Wood | 2021 | Yes | Yes | Unclear | No | No | No | Yes | Yes | Moderate | 4.0 |
| 97 | Sit 2020 a | Cherry Sit | 2020 | Yes | Yes | Unclear | No | No | No | Yes | Yes | Moderate | 4.0 |
| 98 | Pinto dos Santos 2019 a | D. Pinto dos Santos | 2019 | Yes | Yes | Unclear | No | No | No | Yes | Yes | Moderate | 4.0 |

*Q1: inclusion criteria defined; Q2: subjects/setting described; Q3: exposure measured validly; Q4: objective criteria used; Q5: confounders identified; Q6: confounder strategies stated; Q7: outcomes measured validly; Q8: appropriate statistics. Y: yes; N: no; U: unclear. Yes=1; No or Unclear=0. Summary categories (6-8, 4-5, 0-3) are review-defined thresholds for descriptive grouping only; they are not official JBI cut-offs, were not used to exclude any study, and do not replace item-level appraisal.*

**Table S8. Assessment of potential participant overlap among the 96 included studies and related screened records.**

| **Cluster** | **Included study retained** | **Basis for detailed review** | **Final assessment** | **Action** |
| --- | --- | --- | --- | --- |
| 1 | Xu 2024 | Institution and potential related screened record | No second eligible report from the same participant cohort contributed data | Retained |
| 2 | Sorte 2024 | Institution and adjacent study period | No second eligible report from the same participant cohort contributed data | Retained |
| 3 | Doumat 2022 | Author, country, and study period | No second eligible report from the same participant cohort contributed data | Retained |
| 4 | Alwadani 2024 | Institutional and temporal screening concern | No second eligible report from the same participant cohort contributed data | Retained |
| 5-8 | 4 additional clusters | Various | Cleared after investigation | Cleared after investigation; retained with no participant-overlap flag |

All 96 included studies were assessed for potential participant duplication using author lists, ethics-approval information, study setting, institutional affiliation, data-collection period, sample characteristics, and survey-platform details. Eight clusters were flagged for detailed review. Four were cleared after source-level examination. In each of the remaining 4 clusters, only 1 eligible report contributed data to the meta-analysis; therefore, no participant sample was counted more than once. No included study was removed for confirmed participant duplication. The 4 retained studies were included in the prespecified leave-one-out sensitivity analyses to assess their influence on the pooled estimates. External screened records did not contribute data to the synthesis and are not listed as included studies.

**Table S9. Proportion extraction data for 9 outcome domains (P1-P9) across all contributing studies. Values shown as n event/n total; NR: not reported.**

| **#** | **Study ID** | **Year** | **N** | **P1, n/N** | **P2, n/N** | **P3, n/N** | **P4, n/N** | **P5, n/N** | **P6, n/N** | **P7, n/N** | **P8, n/N** | **P9, n/N** |
| --- | --- | --- | --- | --- | --- | --- | --- | --- | --- | --- | --- | --- |
| 1 | Cao 2026 a | 2026 | 1062 | NR | 779/1062 | NR | 461/1062 | NR | 1056/1062 | 762/1062 | 697/1062 | NR |
| 2 | Kara 2026 a | 2026 | 1346 | 737/1346 | NR | 1065/1346 | NR | 1092/1346 | 934/1346 | NR | 907/1346 | 532/1346 |
| 3 | Jamjoom 2026 a | 2026 | 117 | NR | NR | NR | NR | NR | NR | NR | NR | NR |
| 4 | Xu 2026 a | 2026 | 661 | NR | NR | NR | NR | NR | 654/661 | NR | NR | NR |
| 5 | Albusmait 2026 a | 2026 | 350 | 301/350 | 287/350 | 242/350 | 164/350 | 213/350 | NR | NR | NR | NR |
| 6 | Xu 2026 b | 2026 | 177 | 116/157 | 136/157 | 142/177 | 63/177 | 128/177 | 157/177 | 135/157 | NR | NR |
| 7 | Prasad 2026 a | 2026 | 317 | NR | 244/315 | 256/316 | 180/315 | 239/317 | NR | NR | NR | 33/317 |
| 8 | Yousef 2026 a | 2026 | 353 | 248/353 | NR | NR | 105/353 | NR | NR | 277/353 | 246/353 | NR |
| 10 | Hu 2025 a | 2025 | 1133 | NR | NR | NR | NR | NR | 713/1133 | 433/713 | 871/1133 | NR |
| 11 | Ghanem 2025 a | 2025 | 423 | 159/423 | NR | 314/423 | 216/423 | 192/423 | 318/423 | NR | 284/423 | 188/423 |
| 12 | Ahmed 2025 a | 2025 | 1443 | NR | NR | NR | NR | 949/1443 | 605/1443 | NR | NR | NR |
| 13 | Chakri 2025 a | 2025 | 580 | 482/580 | 539/580 | 522/580 | 331/580 | 64/580 | NR | NR | NR | NR |
| 14 | Giavina-Bianchi 2025 a | 2025 | 145 | NR | 125/144 | NR | 43/145 | 85/145 | 61/145 | 107/145 | NR | NR |
| 15 | Fussholler 2025 a | 2025 | 84 | NR | 52/84 | 60/84 | NR | NR | 64/84 | NR | 55/75 | 35/84 |
| 17 | Elhassan 2025 a | 2025 | 293 | 171/293 | NR | NR | NR | 273/293 | NR | 149/293 | 226/293 | NR |
| 18 | Tran 2025 a | 2025 | 166 | NR | NR | 126/164 | NR | NR | 131/166 | 154/165 | 131/166 | NR |
| 19 | Almehmadi 2025 a | 2025 | 170 | NR | 109/170 | NR | 36/170 | 163/170 | 160/170 | NR | 99/170 | NR |
| 20 | Arab-Borzu 2025 a | 2025 | 280 | NR | NR | 211/279 | 214/278 | 168/279 | NR | 229/280 | 169/279 | NR |
| 21 | Onetiu 2025 a | 2025 | 161 | NR | NR | NR | NR | 66/161 | NR | NR | 115/161 | NR |
| 22 | Alfahl 2025 a | 2025 | 189 | 141/189 | NR | 130/189 | 88/189 | 102/189 | 98/189 | NR | NR | NR |
| 23 | Zhang 2025 a | 2025 | 254 | NR | NR | 187/254 | NR | NR | 241/254 | 194/249 | NR | NR |
| 24 | Almuhanna 2025 a | 2025 | 292 | 164/292 | NR | NR | 69/292 | NR | NR | NR | NR | NR |
| 26 | Abdelhafiz 2025 a | 2025 | 614 | 393/614 | NR | NR | NR | 584/614 | 482/614 | 441/614 | 438/614 | 296/614 |
| 27 | Liu 2026 a | 2026 | 788 | NR | NR | NR | 423/788 | 373/788 | 720/788 | NR | 382/788 | NR |
| 28 | Duan 2025 a | 2025 | 553 | 341/553 | NR | NR | NR | 241/553 | NR | 253/553 | NR | NR |
| 29 | Hanifa 2025 a | 2025 | 564 | NR | NR | NR | NR | 439/564 | 157/564 | NR | NR | NR |
| 30 | Fernando 2025 a | 2025 | 304 | 243/304 | NR | 258/304 | NR | 213/304 | NR | NR | 182/304 | NR |
| 31 | Gualda-Gea 2025 a | 2025 | 29 | 19/29 | NR | NR | NR | NR | 9/29 | 24/29 | 14/29 | 17/29 |
| 32 | Ignjatovic 2025 a | 2025 | 1212 | 420/511 | NR | 257/701 | 258/511 | 962/1212 | 511/1212 | NR | 286/511 | NR |
| 33 | Sanri 2025 a | 2025 | 120 | 96/120 | NR | 99/120 | NR | 92/120 | NR | NR | NR | NR |
| 34 | Shah 2025 a | 2025 | 200 | 142/200 | NR | 156/200 | NR | 162/200 | 118/200 | 135/200 | NR | NR |
| 35 | Gopidesi 2025 a | 2025 | 210 | 197/210 | NR | NR | NR | 129/210 | 169/210 | NR | NR | NR |
| 36 | Ringeval 2025 a | 2025 | 361 | NR | NR | 306/361 | NR | NR | NR | NR | NR | NR |
| 37 | Ejas 2025 a | 2025 | 1180 | 608/1180 | NR | NR | NR | 898/1180 | 646/898 | NR | 581/1180 | NR |
| 38 | Mousavi Baigi 2025 a | 2025 | 95 | 84/95 | NR | 75/95 | NR | 52/95 | NR | NR | NR | NR |
| 39 | Falcon 2025 a | 2025 | 20 | 20/20 | NR | 20/20 | NR | NR | NR | NR | 16/20 | NR |
| 40 | Rezazadeh 2025 a | 2025 | 340 | NR | NR | NR | NR | NR | NR | NR | NR | NR |
| 41 | Sunmboye 2025 a | 2025 | 200 | NR | NR | NR | NR | NR | NR | NR | NR | NR |
| 43 | AlZaabi 2025 a | 2025 | 84 | NR | NR | NR | NR | NR | 67/84 | NR | NR | NR |
| 44 | Ziapour 2025 a | 2025 | 800 | NR | NR | NR | NR | NR | NR | NR | NR | NR |
| 45 | Alharkan 2025 a | 2025 | 525 | NR | NR | NR | NR | 320/525 | 320/525 | NR | NR | NR |
| 46 | Naseer 2025 a | 2025 | 153 | NR | NR | NR | NR | NR | NR | NR | NR | NR |
| 47 | Gonzalez-Bravo 2025 a | 2025 | 194 | NR | NR | NR | NR | NR | 141/194 | NR | NR | NR |
| 48 | Almarzouki 2025 a | 2025 | 354 | NR | NR | NR | NR | NR | NR | 229/354 | NR | NR |
| 50 | Allam 2024 b | 2024 | 4492 | 3815/4492 | 3183/4492 | 3132/4492 | 645/4492 | 578/4492 | NR | NR | NR | NR |
| 51 | Jebreen 2024 a | 2024 | 349 | 240/349 | NR | 247/349 | NR | NR | NR | 228/349 | NR | NR |
| 52 | Allam 2024 c | 2024 | 999 | NR | NR | NR | NR | NR | NR | NR | NR | NR |
| 53 | Tao 2024 a | 2024 | 693 | 415/693 | NR | NR | NR | 202/693 | 199/693 | NR | 635/693 | NR |
| 54 | Ganjavi 2024 a | 2024 | 415 | NR | NR | NR | NR | 398/415 | 216/415 | NR | NR | NR |
| 55 | Alwadani 2024 a | 2024 | 303 | 264/303 | 213/303 | 179/303 | 139/303 | 115/303 | 42/303 | NR | NR | NR |
| 56 | Abu Hammour 2024 a | 2024 | 244 | 212/244 | NR | NR | NR | 136/221 | 177/244 | NR | 191/244 | NR |
| 57 | Faroog 2024 a | 2024 | 345 | 340/345 | NR | 332/345 | 49/345 | 294/345 | NR | NR | 8/345 | NR |
| 59 | Amin 2024 a | 2024 | 762 | 603/762 | NR | NR | 240/762 | 642/762 | 267/762 | NR | 285/762 | NR |
| 60 | Jackson 2024 a | 2024 | 325 | 186/325 | NR | 250/325 | 122/325 | NR | NR | NR | 174/325 | NR |
| 61 | Angkurawaranon 2024 a | 2024 | 352 | 330/352 | 312/352 | 271/352 | 159/352 | 111/352 | NR | NR | NR | NR |
| 63 | Daher 2024 a | 2024 | 365 | 265/365 | NR | 351/365 | 90/365 | 96/365 | NR | NR | NR | NR |
| 64 | Weidener 2024 a | 2024 | 487 | 349/487 | NR | 365/487 | 167/487 | NR | 189/487 | 404/487 | 464/487 | NR |
| 65 | Luong 2025 a | 2025 | 223 | NR | NR | 92/220 | NR | NR | NR | NR | NR | NR |
| 66 | Al Shahrani 2024 a | 2024 | 572 | NR | NR | NR | NR | NR | 533/572 | NR | NR | NR |
| 67 | Malesevic 2024 a | 2024 | 1701 | NR | NR | NR | NR | 650/1701 | NR | 762/1701 | 1005/1701 | NR |
| 68 | Hamad 2024 a | 2024 | 858 | NR | NR | NR | NR | 631/858 | NR | NR | NR | NR |
| 69 | Alammari 2024 a | 2024 | 207 | 184/207 | NR | NR | 71/207 | 156/207 | NR | NR | NR | NR |
| 70 | Gandhi 2024 a | 2024 | 605 | NR | NR | NR | NR | NR | 278/605 | 444/605 | NR | NR |
| 71 | Ozbek Guven 2024 a | 2024 | 542 | NR | NR | NR | NR | 530/542 | NR | NR | NR | NR |
| 72 | Xu 2024 a | 2024 | 102 | NR | NR | 102/102 | NR | 102/102 | 70/102 | 55/70 | NR | 54/70 |
| 74 | Al-Roomi 2024 a | 2024 | 152 | NR | NR | 104/152 | NR | 133/152 | 67/152 | NR | 29/151 | NR |
| 76 | Alkhaaldi 2023 a | 2023 | 265 | 155/265 | 78/265 | NR | NR | NR | NR | NR | 168/265 | NR |
| 77 | Tangadulrat 2023 a | 2023 | 124 | 94/124 | NR | NR | NR | NR | NR | NR | NR | 110/124 |
| 78 | Oluwadiya 2023 a | 2023 | 708 | NR | NR | NR | 408/708 | NR | NR | NR | 488/691 | NR |
| 79 | Tung 2023 a | 2023 | 301 | 263/301 | 214/301 | 209/301 | 180/301 | 93/301 | NR | NR | NR | NR |
| 80 | Stewart 2023 a | 2023 | 134 | 100/134 | NR | 79/134 | NR | 114/134 | NR | NR | NR | NR |
| 81 | Al Hadithy 2023 a | 2023 | 221 | NR | NR | NR | 174/221 | NR | 54/221 | NR | NR | NR |
| 82 | Buabbas 2023 a | 2023 | 352 | 349/352 | 329/352 | 289/352 | NR | 213/352 | NR | NR | NR | NR |
| 83 | Barreiro-Ares 2023 a | 2023 | 281 | NR | NR | 225/281 | NR | 223/281 | NR | NR | NR | NR |
| 84 | Biri 2023 a | 2023 | 172 | NR | NR | NR | NR | NR | NR | NR | NR | NR |
| 85 | Doumat 2022 a | 2022 | 206 | 147/206 | NR | NR | 115/206 | NR | NR | NR | NR | NR |
| 86 | Ahmed 2022 a | 2022 | 247 | 183/247 | NR | NR | NR | 170/247 | 20/247 | NR | NR | NR |
| 87 | Liu 2022 a | 2022 | 390 | 308/388 | 355/388 | NR | 70/386 | NR | NR | NR | NR | NR |
| 88 | Caparros Galan and Sendra Portero 2022 a | 2022 | 341 | NR | NR | 211/341 | 125/341 | 177/341 | NR | NR | NR | NR |
| 89 | Gillissen 2022 a | 2022 | 1053 | NR | NR | NR | NR | NR | NR | NR | NR | NR |
| 90 | Pucchio 2022 a | 2022 | 486 | 382/455 | NR | 304/455 | 94/461 | 216/463 | NR | 360/462 | NR | NR |
| 91 | McLennan 2022 a | 2022 | 844 | 484/844 | NR | NR | NR | 300/844 | NR | NR | NR | NR |
| 92 | Blease 2022 a | 2022 | 252 | NR | NR | 190/242 | NR | 137/242 | NR | 99/241 | NR | NR |
| 93 | Boillat 2022 a | 2022 | 102 | NR | NR | NR | NR | NR | NR | NR | NR | NR |
| 94 | Blacketer 2021 a | 2021 | 245 | NR | NR | NR | NR | NR | NR | NR | NR | NR |
| 95 | Bisdas 2021 a | 2021 | 2495 | NR | NR | NR | NR | NR | NR | NR | NR | NR |
| 96 | Wood 2021 a | 2021 | 117 | NR | NR | NR | NR | 35/117 | NR | 103/117 | NR | NR |
| 97 | Sit 2020 a | 2020 | 484 | 432/484 | 430/484 | 378/484 | 234/484 | 214/484 | NR | NR | NR | NR |
| 98 | Pinto dos Santos 2019 a | 2019 | 263 | 220/263 | NR | 187/263 | 40/263 | 81/263 | NR | NR | NR | NR |

*P1: positive attitude; P2: career benefit; P3: curricular integration; P4: replacement concern; P5: familiarity; P6: prior AI use; P7: willingness to adopt; P8: ethical concerns; P9: trust in AI.*

**Table S10. Continuous extraction data for MAIRS-MS and other continuous scales. Values shown as mean(SD) where both mean and SD were reported; NR: not reported or not eligible for pooled mean extraction.**

| **#** | **Study ID** | **Year** | **N** | **Instrument/scale** | **MAIRS-MS total** | **Cognition** | **Ability** | **Vision** | **Ethics** |
| --- | --- | --- | --- | --- | --- | --- | --- | --- | --- |
| 9 | Catan-Inan 2026 a | 2026 | 183 | MAIRS(22–110) | 75.09(19.53) | NR | NR | NR | NR |
| 16 | Tajima 2025 a | 2025 | 118 | Single-item willingness to learn about GenAI(1–5) | NR | NR | NR | NR | NR |
| 20 | Arab-Borzu 2025 a | 2025 | 280 | MAIRS-MS (Persian version)(22–110) | 74.13(12.7) | 25.12(5.77) | 20.08(5.08) | 10.29(2.26) | 10.81(2.09) |
| 26 | Abdelhafiz 2025 a | 2025 | 614 | Attitude score toward ChatGPT and similar chatbots(1–5) | NR | NR | NR | NR | NR |
| 27 | Liu 2026 a | 2026 | 788 | Perceived usefulness (PU) scale(1–5) | NR | NR | NR | NR | NR |
| 29 | Hanifa 2025 a | 2025 | 564 | Arabic MAIRS MS readiness scale(22–110) | 53.45(15.56) | 21.1(6.26) | 18.77(6.31) | 7.73(2.58) | 5.85(2.53) |
| 36 | Ringeval 2025 a | 2025 | 361 | Adapted survey instrument grounded in TAM/TIB (multiple single-item dHealth means)(1–5) | NR | NR | NR | NR | NR |
| 40 | Rezazadeh 2025 a | 2025 | 340 | Persian MAIRS-MS questionnaire(20–100) | 59.21(16.12) | 20.3(7.04) | 21.88(6.74) | 6.09(1.94) | 10.94(3.04) |
| 43 | AlZaabi 2025 a | 2025 | 84 | MAIRS-MS questionnaire (item means reported)(1–5 item mean) | NR | NR (mean only; SD not reported) | NR (mean only; SD not reported) | NR (mean only; SD not reported) | NR (mean only; SD not reported) |
| 44 | Ziapour 2025 a | 2025 | 800 | Persian MAIRS-MS questionnaire(22–110) | 70.59(19.24) | 25.98(7.36) | 25.74(7.52) | 9.73(2.96) | 9.15(3.36) |
| 46 | Naseer 2025 a | 2025 | 153 | Composite attitude score toward AI integration (student subgroup)(1–5) | NR | NR | NR | NR | NR |
| 49 | Dhurandhar 2025 a | 2025 | 482 | Medical Artificial Intelligence Readiness Scale for Medical Students (MAIRS-MS)(22–110) | 74.61(10.137) | 26.23(4.417) | 27.62(4.372) | 10.37(1.803) | 10.39(1.789) |
| 58 | Laupichler 2024 a | 2024 | 377 | SNAIL overall AI literacy score(1–7) | NR | NR | NR | NR | NR |
| 62 | Lugito 2024 a | 2024 | 650 | MAIRS-MS(22–110) | 73.34(13.98) | 24.52(5.26) | 27.78(4.65) | 10.57(2.07) | 10.47(2) |
| 63 | Daher 2024 a | 2024 | 365 | MAIRS-MS-based readiness categorization only; means not reported(5-point Likert / means NR) | NR | NR | NR | NR | NR |
| 64 | Weidener 2024 a | 2024 | 487 | Newly developed 53-item web survey (categorical-only extractable)(NR) | NR | NR | NR | NR | NR |
| 65 | Luong 2025 a | 2025 | 223 | MAIRS-MS (item-average scores)(1–5) | 3.18(1.03) | 2.95(1.06) | 3.2(1.04) | 3.54(0.9) | 3.42(0.9) |
| 66 | Al Shahrani 2024 a | 2024 | 572 | MAIRS-MS(22–110) | 68.39(18.316) | 22.69(7.369) | 26.12(7.302) | 8.97(2.996) | 10.61(3.268) |
| 68 | Hamad 2024 a | 2024 | 858 | MAIRS-MS(22–110) | 64.267(18.26) | 22.57(6.67) | 23.38(7.16) | 9.023(2.99) | 9.292(3.06) |
| 70 | Gandhi 2024 a | 2024 | 605 | Validated mixed-method survey (no pooled continuous extractable outcome)(NR) | NR | NR | NR | NR | NR |
| 71 | Ozbek Guven 2024 a | 2024 | 542 | Medical AI Readiness Scale(22–110) | 61(15.58) | 18.56(5.31) | 23.8(7.67) | 8.58(2.94) | 10.05(2.9) |
| 73 | Sorte 2024 a | 2024 | 217 | Validated exploratory survey (domain percentage scores)(0–100) | NR | NR | NR | NR | NR |
| 75 | Liu 2024 a | 2024 | 311 | Technology Acceptance Model (attitude toward use of LLMs)(1–5) | NR | NR | NR | NR | NR |
| 76 | Alkhaaldi 2023 a | 2023 | 265 | Cross-sectional survey of ChatGPT/AI experiences and perceptions (categorical-only extractable)(NR) | NR | NR | NR | NR | NR |
| 78 | Oluwadiya 2023 a | 2023 | 708 | Adapted KAP survey; medians only / no pooled continuous extractable outcome(NR) | NR | NR | NR | NR | NR |
| 79 | Tung 2023 a | 2023 | 301 | Medical Artificial Intelligence Readiness Scale for Medical Students (MAIRS-MS)(22–110) | 67(14.8) | 21(5.8) | 25(6.8) | 10(2.6) | 11(2.7) |
| 82 | Buabbas 2023 a | 2023 | 352 | No extractable continuous summary outcome(NR) | NR | NR | NR | NR | NR |
| 84 | Biri 2023a | 2023 | 172 | LLM KAP questionnaire (attitude domain)(1–5) | NR | NR | NR | NR | NR |
| 89 | Gillissen 2022a | 2022 | 1053 | No extractable continuous summary outcome(NR) | NR | NR | NR | NR | NR |
| 93 | Boillat 2022 a | 2022 | 102 | Overall familiarity with AIM factor (medical student subgroup)(1–5) | NR | NR | NR | NR | NR |
| 96 | Wood 2021 a | 2021 | 117 | Single-item: AI should be part of medical education and training (medical student subgroup)(1–5) | NR | NR | NR | NR | NR |

*Instrument/scale identifies the reported continuous instrument or scale. MAIRS-MS total and subscale columns contain mean(SD) only when both mean and SD were available. Rows reporting scale information only, medians, categories, or means without SD are marked NR for SD-required pooled mean extraction; AlZaabi* 2025 *reported subscale means without SD and was not included in pooled subscale analyses. MAIRS-MS: Medical AI Readiness Scale for Medical Students.*

**Table S11. Full outcome harmonization table for study-domain contributions.**

| Study (first author, year) | Domain | Original item wording (or closest reported paraphrase) | Response scale | Scale points | Dichotomization rule | Neutral-response handling | Events | Total | Proportion | Domain-assignment rationale | Included in 96-study synthesis (Y/N) |
| --- | --- | --- | --- | --- | --- | --- | --- | --- | --- | --- | --- |
| Ali Veysel Kara 2026 | P1 | Overall perception of AI in medicine (agree + strongly agree) | Likert agreement scale; point count not reported in the source article | Not clearly reported | Agree + Strongly agree vs all other item responses | Neutral responses, if available, were excluded from numerator and retained in denominator | 737 | 1346 | 0.5475 | Mapped to P1 (Positive general attitude toward AI in healthcare) under operational definition: Proportion of students expressing a generally positive, favorable, or supportive attitude toward AI in medicine/healthcare. | Y |
| Amna Albusmait 2026 | P1 | AI will play an important role in healthcare (agree + strongly agree) | 5-point Likert agreement scale: strongly disagree to strongly agree | 5 | Agree + Strongly agree vs all other item responses | Neutral responses, if available, were excluded from numerator and retained in denominator | 301 | 350 | 0.8600 | Mapped to P1 (Positive general attitude toward AI in healthcare) under operational definition: Proportion of students expressing a generally positive, favorable, or supportive attitude toward AI in medicine/healthcare. | Y |
| Alan Yuesheng Xu 2026 | P1 | Experience with ChatGPT improved overall perception of AI’s potential to assist in medical practice | Author-reported proportion; response scale not reported in the source article | Not clearly reported | Author-reported proportion; numerator is the endorsed response category | Neutral handling not explicitly reported; denominator retained as reported | 116 | 157 | 0.7389 | Mapped to P1 (Positive general attitude toward AI in healthcare) under operational definition: Proportion of students expressing a generally positive, favorable, or supportive attitude toward AI in medicine/healthcare. | Y |
| Yacoub A. Yousef 2026 | P1 | AI in ophthalmology viewed as a complementary tool rather than a replacement for ophthalmologists | response scale not reported in the source article | Not reported | Dichotomization method not reported in primary study; proportion extracted from reported percentage | Neutral handling not explicitly reported; denominator retained as reported | 248 | 353 | 0.7025 | Mapped to P1 (Positive general attitude toward AI in healthcare) under operational definition: Proportion of students expressing a generally positive, favorable, or supportive attitude toward AI in medicine/healthcare. | Y |
| Omar A. Ghanem 2025 | P1 | General attitude towards the application of AI in medicine (strongly agree + agree) | Likert agreement scale; point count not reported in the source article | Not clearly reported | Agree + Strongly agree vs all other item responses | Neutral responses, if available, were excluded from numerator and retained in denominator | 159 | 423 | 0.3759 | Mapped to P1 (Positive general attitude toward AI in healthcare) under operational definition: Proportion of students expressing a generally positive, favorable, or supportive attitude toward AI in medicine/healthcare. | Y |
| Imad Chakri 2025 | P1 | AI in medicine perceived as a partner rather than a competitor | Author-reported proportion; response scale not reported in the source article | Not clearly reported | Author-reported proportion; numerator is the endorsed response category | Neutral handling not explicitly reported; denominator retained as reported | 482 | 580 | 0.8310 | Mapped to P1 (Positive general attitude toward AI in healthcare) under operational definition: Proportion of students expressing a generally positive, favorable, or supportive attitude toward AI in medicine/healthcare. | Y |
| Safia Elwaleed Elhassan 2025 | P1 | ChatGPT/other chat-based AI apps can enhance my medical education | Author-reported proportion; response scale not reported in the source article | Not clearly reported | Author-reported proportion; numerator is the endorsed response category | Neutral response category reported; not counted in numerator and retained in denominator unless source denominator was item-specific | 171 | 293 | 0.5836 | Mapped to P1 (Positive general attitude toward AI in healthcare) under operational definition: Proportion of students expressing a generally positive, favorable, or supportive attitude toward AI in medicine/healthcare. | Y |
| Samah Alfahl 2025 | P1 | AI would revolutionize education | 5-point Likert agreement scale: strongly disagree to strongly agree | 5 | Author-reported proportion; numerator is the endorsed response category | Neutral handling not explicitly reported; denominator retained as reported | 141 | 189 | 0.7460 | Mapped to P1 (Positive general attitude toward AI in healthcare) under operational definition: Proportion of students expressing a generally positive, favorable, or supportive attitude toward AI in medicine/healthcare. | Y |
| Afnan Almuhanna 2025 | P1 | Belief that radiology AI can improve radiologist efficiency | Likert agreement scale; point count not reported in the source article | Not clearly reported | Agree + Strongly agree vs all other item responses | Neutral responses, if available, were excluded from numerator and retained in denominator | 164 | 292 | 0.5616 | Mapped to P1 (Positive general attitude toward AI in healthcare) under operational definition: Proportion of students expressing a generally positive, favorable, or supportive attitude toward AI in medicine/healthcare. | Y |
| Abdul Sami 2025 | P1 | AI could improve healthcare | 5-point Likert agreement scale: strongly disagree to strongly agree | 5 | Author-reported proportion; numerator is the endorsed response category | Neutral handling not explicitly reported; denominator retained as reported | 564 | 702 | 0.8034 | Mapped to P1 (Positive general attitude toward AI in healthcare) under operational definition: Proportion of students expressing a generally positive, favorable, or supportive attitude toward AI in medicine/healthcare. | N |
| Ahmed Samir Abdelhafiz 2025 | P1 | ChatGPT provides accurate medical information (helpful) | Author-reported proportion; response scale not reported in the source article | Not clearly reported | Author-reported proportion; numerator is the endorsed response category | Neutral response category reported; not counted in numerator and retained in denominator unless source denominator was item-specific | 393 | 614 | 0.6401 | Mapped to P1 (Positive general attitude toward AI in healthcare) under operational definition: Proportion of students expressing a generally positive, favorable, or supportive attitude toward AI in medicine/healthcare. | Y |
| Shuo Duan 2025 | P1 | AI could have a positive impact on personalized learning and timely feedback (agree + strongly agree) | 5-point Likert agreement scale: strongly disagree to strongly agree | 5 | Agree + Strongly agree vs all other item responses | Neutral responses, if available, were excluded from numerator and retained in denominator | 341 | 553 | 0.6166 | Mapped to P1 (Positive general attitude toward AI in healthcare) under operational definition: Proportion of students expressing a generally positive, favorable, or supportive attitude toward AI in medicine/healthcare. | Y |
| Poorna Fernando 2025 | P1 | Believed AI would play a significant role in the future of medicine | Author-reported proportion; response scale not reported in the source article | Not clearly reported | Author-reported proportion; numerator is the endorsed response category | Neutral handling not explicitly reported; denominator retained as reported | 243 | 304 | 0.7993 | Mapped to P1 (Positive general attitude toward AI in healthcare) under operational definition: Proportion of students expressing a generally positive, favorable, or supportive attitude toward AI in medicine/healthcare. | Y |
| Juan José Gualda-Gea 2025 | P1 | Strong interest in the practical applications of AI in medicine | Author-reported proportion; response scale not reported in the source article | Not clearly reported | Author-reported proportion; numerator is the endorsed response category | Neutral handling not explicitly reported; denominator retained as reported | 19 | 29 | 0.6552 | Mapped to P1 (Positive general attitude toward AI in healthcare) under operational definition: Proportion of students expressing a generally positive, favorable, or supportive attitude toward AI in medicine/healthcare. | Y |
| Aleksandra Ignjatović 2025 | P1 | I feel optimistic about ChatGPT! | response scale not reported in the source article | Not reported | Dichotomization method not reported in primary study; proportion extracted from reported percentage | Neutral handling not explicitly reported; denominator retained as reported | 420 | 511 | 0.8219 | Mapped to P1 (Positive general attitude toward AI in healthcare) under operational definition: Proportion of students expressing a generally positive, favorable, or supportive attitude toward AI in medicine/healthcare. | Y |
| Emre Sanri 2025 | P1 | Positive views on AI's role in clinical decision-making processes | Author-reported proportion; response scale not reported in the source article | Not clearly reported | Author-reported proportion; numerator is the endorsed response category | Neutral handling not explicitly reported; denominator retained as reported | 96 | 120 | 0.800 | Mapped to P1 (Positive general attitude toward AI in healthcare) under operational definition: Proportion of students expressing a generally positive, favorable, or supportive attitude toward AI in medicine/healthcare. | Y |
| M Hassaan Shah 2025 | P1 | Belief that AI can enhance medical education | Author-reported proportion; response scale not reported in the source article | Not clearly reported | Author-reported proportion; numerator is the endorsed response category | Neutral response category reported; not counted in numerator and retained in denominator unless source denominator was item-specific | 142 | 200 | 0.7100 | Mapped to P1 (Positive general attitude toward AI in healthcare) under operational definition: Proportion of students expressing a generally positive, favorable, or supportive attitude toward AI in medicine/healthcare. | Y |
| Madhulatha Gopidesi 2025 | P1 | Positive attitude toward AI tools during the course of medicine | Author-reported proportion; response scale not reported in the source article | Not clearly reported | Author-reported proportion; numerator is the endorsed response category | Neutral handling not explicitly reported; denominator retained as reported | 197 | 210 | 0.9381 | Mapped to P1 (Positive general attitude toward AI in healthcare) under operational definition: Proportion of students expressing a generally positive, favorable, or supportive attitude toward AI in medicine/healthcare. | Y |
| Faiza Ejas 2025 | P1 | Support use of LLMs in healthcare (closest reported paraphrase; exact source wording not verified) | Author-reported proportion; response scale not reported in the source article | Not clearly reported | Author-reported proportion; numerator is the endorsed response category | Neutral handling not explicitly reported; denominator retained as reported | 608 | 1180 | 0.5153 | Mapped to P1 (Positive general attitude toward AI in healthcare) under operational definition: Proportion of students expressing a generally positive, favorable, or supportive attitude toward AI in medicine/healthcare. | Y |
| Seyyedeh Fatemeh Mousavi Baigi 2025 | P1 | Support development of AI in field of study (scores 6–10 on 0–10 scale) | Author-reported proportion; response scale not reported in the source article | Not clearly reported | Author-reported proportion; numerator is the endorsed response category | Neutral handling not explicitly reported; denominator retained as reported | 84 | 95 | 0.8842 | Mapped to P1 (Positive general attitude toward AI in healthcare) under operational definition: Proportion of students expressing a generally positive, favorable, or supportive attitude toward AI in medicine/healthcare. | Y |
| Robbi Miguel G. Falcon 2025 | P1 | AI had useful applications in the medical field (agree + strongly agree) | 5-point Likert agreement scale: strongly disagree to strongly agree | 5 | Agree + Strongly agree vs all other item responses | Neutral responses, if available, were excluded from numerator and retained in denominator | 20 | 20 | 1.0000 | Mapped to P1 (Positive general attitude toward AI in healthcare) under operational definition: Proportion of students expressing a generally positive, favorable, or supportive attitude toward AI in medicine/healthcare. | Y |
| Muhammad Farooq and Usmani (2025) | P1 | AI will have a positive impact on the learning experience | response scale not reported in the source article | Not reported | Dichotomization method not reported in primary study; proportion extracted from reported percentage | Neutral handling not explicitly reported; denominator retained as reported | 111 | 152 | 0.7303 | Mapped to P1 (Positive general attitude toward AI in healthcare) under operational definition: Proportion of students expressing a generally positive, favorable, or supportive attitude toward AI in medicine/healthcare. | N |
| Ahmed Hafez Allam 2024 | P1 | Artificial intelligence will revolutionize medicine in general (agree/strongly agree) | Likert agreement scale; point count not reported in the source article | Not clearly reported | Agree + Strongly agree vs all other item responses | Neutral responses, if available, were excluded from numerator and retained in denominator | 3815 | 4492 | 0.8493 | Mapped to P1 (Positive general attitude toward AI in healthcare) under operational definition: Proportion of students expressing a generally positive, favorable, or supportive attitude toward AI in medicine/healthcare. | Y |
| Kamel Jebreen 2024 | P1 | AI would revolutionize medical fields (agree/strongly agree) | Likert agreement scale; point count not reported in the source article | Not clearly reported | Agree + Strongly agree vs all other item responses | Neutral responses, if available, were excluded from numerator and retained in denominator | 240 | 349 | 0.6877 | Mapped to P1 (Positive general attitude toward AI in healthcare) under operational definition: Proportion of students expressing a generally positive, favorable, or supportive attitude toward AI in medicine/healthcare. | Y |
| Wenjuan Tao 2024 | P1 | Agree/strongly agree with the use of AI chatbot applications for study or work | 5-point Likert agreement scale: strongly disagree to strongly agree | 5 | Agree + Strongly agree vs all other item responses | Neutral responses, if available, were excluded from numerator and retained in denominator | 415 | 693 | 0.5988 | Mapped to P1 (Positive general attitude toward AI in healthcare) under operational definition: Proportion of students expressing a generally positive, favorable, or supportive attitude toward AI in medicine/healthcare. | Y |
| Fahad Abdullah Saeed Alwadani 2024 | P1 | AI will play an important role in healthcare (agree + strongly agree) | 5-point Likert agreement scale: strongly disagree to strongly agree | 5 | Agree + Strongly agree vs all other item responses | Neutral responses, if available, were excluded from numerator and retained in denominator | 264 | 303 | 0.8713 | Mapped to P1 (Positive general attitude toward AI in healthcare) under operational definition: Proportion of students expressing a generally positive, favorable, or supportive attitude toward AI in medicine/healthcare. | Y |
| Adnan Abu Hammour 2024 | P1 | Medical students can benefit from using ChatGPT (agree + strongly agree) | 5-point Likert agreement scale: strongly disagree to strongly agree | 5 | Agree + Strongly agree vs all other item responses | Neutral responses, if available, were excluded from numerator and retained in denominator | 212 | 244 | 0.8689 | Mapped to P1 (Positive general attitude toward AI in healthcare) under operational definition: Proportion of students expressing a generally positive, favorable, or supportive attitude toward AI in medicine/healthcare. | Y |
| Zainudheen Faroog 2024 | P1 | AI would be a helpful tool in ophthalmology (agree) | Author-reported proportion; response scale not reported in the source article | Not clearly reported | Author-reported proportion; numerator is the endorsed response category | Neutral handling not explicitly reported; denominator retained as reported | 340 | 345 | 0.9855 | Mapped to P1 (Positive general attitude toward AI in healthcare) under operational definition: Proportion of students expressing a generally positive, favorable, or supportive attitude toward AI in medicine/healthcare. | Y |
| Mohammed Hammad Jaber Amin 2024 | P1 | AI is essential in the medical field (agree + strongly agree) (closest reported paraphrase; exact source wording not verified) | Likert agreement scale; point count not reported in the source article | Not clearly reported | Agree + Strongly agree vs all other item responses | Neutral responses, if available, were excluded from numerator and retained in denominator | 603 | 762 | 0.7913 | Mapped to P1 (Positive general attitude toward AI in healthcare) under operational definition: Proportion of students expressing a generally positive, favorable, or supportive attitude toward AI in medicine/healthcare. | Y |
| Preetha Jackson 2024 | P1 | AI is an assistive technology for reducing medical errors | 5-point Likert agreement scale: strongly disagree to strongly agree | 5 | Author-reported proportion; numerator is the endorsed response category | Neutral handling not explicitly reported; denominator retained as reported | 186 | 325 | 0.5723 | Mapped to P1 (Positive general attitude toward AI in healthcare) under operational definition: Proportion of students expressing a generally positive, favorable, or supportive attitude toward AI in medicine/healthcare. | Y |
| Salita Angkurawaranon 2024 | P1 | Artificial intelligence will play an important role in healthcare (agree + strongly agree) | 5-point Likert agreement scale: strongly disagree to strongly agree | 5 | Agree + Strongly agree vs all other item responses | Neutral responses, if available, were excluded from numerator and retained in denominator | 330 | 352 | 0.9375 | Mapped to P1 (Positive general attitude toward AI in healthcare) under operational definition: Proportion of students expressing a generally positive, favorable, or supportive attitude toward AI in medicine/healthcare. | Y |
| Omar A. Daher 2024 | P1 | These developments in AI make medicine more exciting | 5-point Likert agreement scale: strongly disagree to strongly agree | 5 | Author-reported proportion; numerator is the endorsed response category | Neutral handling not explicitly reported; denominator retained as reported | 265 | 365 | 0.7260 | Mapped to P1 (Positive general attitude toward AI in healthcare) under operational definition: Proportion of students expressing a generally positive, favorable, or supportive attitude toward AI in medicine/healthcare. | Y |
| Lukas Weidener 2024 | P1 | S1: positively change medicine (agree + strongly agree) | 5-point Likert agreement scale: strongly disagree to strongly agree | 5 | Agree + Strongly agree vs all other item responses | Neutral responses, if available, were excluded from numerator and retained in denominator | 349 | 487 | 0.7166 | Mapped to P1 (Positive general attitude toward AI in healthcare) under operational definition: Proportion of students expressing a generally positive, favorable, or supportive attitude toward AI in medicine/healthcare. | Y |
| Dalia M. Alammari 2024 | P1 | AI can enrich medical education/learning | Author-reported proportion; response scale not reported in the source article | Not clearly reported | Author-reported proportion; numerator is the endorsed response category | Neutral handling not explicitly reported; denominator retained as reported | 184 | 207 | 0.8889 | Mapped to P1 (Positive general attitude toward AI in healthcare) under operational definition: Proportion of students expressing a generally positive, favorable, or supportive attitude toward AI in medicine/healthcare. | Y |
| Saif M I Alkhaaldi 2023 | P1 | AI will improve patient care | Author-reported proportion; response scale not reported in the source article | Not clearly reported | Author-reported proportion; numerator is the endorsed response category | Neutral handling not explicitly reported; denominator retained as reported | 155 | 265 | 0.5849 | Mapped to P1 (Positive general attitude toward AI in healthcare) under operational definition: Proportion of students expressing a generally positive, favorable, or supportive attitude toward AI in medicine/healthcare. | Y |
| Pasin Tangadulrat 2023 | P1 | Perceptions towards using AI chatbot for patient care (positive) | Author-reported proportion; response scale not reported in the source article | Not clearly reported | Author-reported proportion; numerator is the endorsed response category | Neutral handling not explicitly reported; denominator retained as reported | 94 | 124 | 0.7581 | Mapped to P1 (Positive general attitude toward AI in healthcare) under operational definition: Proportion of students expressing a generally positive, favorable, or supportive attitude toward AI in medicine/healthcare. | Y |
| Alvin Yong Zong Tung 2023 | P1 | AI will play an important role in healthcare | 5-point Likert agreement scale: strongly disagree to strongly agree | 5 | Author-reported proportion; numerator is the endorsed response category | Neutral handling not explicitly reported; denominator retained as reported | 263 | 301 | 0.8738 | Mapped to P1 (Positive general attitude toward AI in healthcare) under operational definition: Proportion of students expressing a generally positive, favorable, or supportive attitude toward AI in medicine/healthcare. | Y |
| Jonathon Stewart 2023 | P1 | Artificial intelligence will improve medicine in general | response scale not reported in the source article | Not reported | Dichotomization method not reported in primary study; proportion extracted from reported percentage | Neutral handling not explicitly reported; denominator retained as reported | 100 | 134 | 0.7463 | Mapped to P1 (Positive general attitude toward AI in healthcare) under operational definition: Proportion of students expressing a generally positive, favorable, or supportive attitude toward AI in medicine/healthcare. | Y |
| Ali Jasem Buabbas 2023 | P1 | Artificial intelligence will play an important role in healthcare | Author-reported proportion; response scale not reported in the source article | Not clearly reported | Author-reported proportion; numerator is the endorsed response category | Neutral handling not explicitly reported; denominator retained as reported | 349 | 352 | 0.9915 | Mapped to P1 (Positive general attitude toward AI in healthcare) under operational definition: Proportion of students expressing a generally positive, favorable, or supportive attitude toward AI in medicine/healthcare. | Y |
| George Doumat 2022 | P1 | The developments of AI make medicine more interesting to me | Author-reported proportion; response scale not reported in the source article | Not clearly reported | Author-reported proportion; numerator is the endorsed response category | Neutral handling not explicitly reported; denominator retained as reported | 147 | 206 | 0.7136 | Mapped to P1 (Positive general attitude toward AI in healthcare) under operational definition: Proportion of students expressing a generally positive, favorable, or supportive attitude toward AI in medicine/healthcare. | Y |
| Zaboor Ahmed 2022 | P1 | AI is essential in the field of medicine | Likert agreement scale; point count not reported in the source article | Not clearly reported | Author-reported proportion; numerator is the endorsed response category | Neutral handling not explicitly reported; denominator retained as reported | 183 | 247 | 0.7409 | Mapped to P1 (Positive general attitude toward AI in healthcare) under operational definition: Proportion of students expressing a generally positive, favorable, or supportive attitude toward AI in medicine/healthcare. | Y |
| David Shalom Liu 2022 | P1 | Excited to use artificial intelligence technologies | 5-point Likert agreement scale: strongly disagree to strongly agree | 5 | Author-reported proportion; numerator is the endorsed response category | Neutral handling not explicitly reported; denominator retained as reported | 308 | 388 | 0.7938 | Mapped to P1 (Positive general attitude toward AI in healthcare) under operational definition: Proportion of students expressing a generally positive, favorable, or supportive attitude toward AI in medicine/healthcare. | Y |
| Aidan Pucchio 2022 | P1 | Artificial intelligence will improve medicine in the future | Author-reported proportion; response scale not reported in the source article | Not clearly reported | Author-reported proportion; numerator is the endorsed response category | Neutral handling not explicitly reported; denominator retained as reported | 382 | 455 | 0.8396 | Mapped to P1 (Positive general attitude toward AI in healthcare) under operational definition: Proportion of students expressing a generally positive, favorable, or supportive attitude toward AI in medicine/healthcare. | Y |
| Stuart McLennan 2022 | P1 | AI has useful applications in medicine | Author-reported proportion; response scale not reported in the source article | Not clearly reported | Author-reported proportion; numerator is the endorsed response category | Neutral handling not explicitly reported; denominator retained as reported | 484 | 844 | 0.5735 | Mapped to P1 (Positive general attitude toward AI in healthcare) under operational definition: Proportion of students expressing a generally positive, favorable, or supportive attitude toward AI in medicine/healthcare. | Y |
| Cherry Sit 2020 | P1 | AI will play an important role in healthcare | Author-reported proportion; response scale not reported in the source article | Not clearly reported | Author-reported proportion; numerator is the endorsed response category | Neutral handling not explicitly reported; denominator retained as reported | 432 | 484 | 0.8926 | Mapped to P1 (Positive general attitude toward AI in healthcare) under operational definition: Proportion of students expressing a generally positive, favorable, or supportive attitude toward AI in medicine/healthcare. | Y |
| D. Pinto dos Santos 2019 | P1 | Artificial intelligence will improve medicine in general (agree entirely + rather agree) | 4-point Likert agreement scale | 4 | Author-reported proportion; numerator is the endorsed response category | Neutral handling not explicitly reported; denominator retained as reported | 220 | 263 | 0.8365 | Mapped to P1 (Positive general attitude toward AI in healthcare) under operational definition: Proportion of students expressing a generally positive, favorable, or supportive attitude toward AI in medicine/healthcare. | Y |
| Xi Cao 2026 | P2 | Broad knowledge coverage to complement one's own knowledge gaps | Author-reported proportion; response scale not reported in the source article | Not clearly reported | Author-reported proportion; numerator is the endorsed response category | Neutral handling not explicitly reported; denominator retained as reported | 779 | 1062 | 0.7335 | Mapped to P2 (AI perceived as beneficial to medical career) under operational definition: Proportion agreeing that AI will be beneficial, advantageous, or valuable for their future medical career or clinical practice. | Y |
| Amna Albusmait 2026 | P2 | Learning in AI will be beneficial for my career (agree + strongly agree) | 5-point Likert agreement scale: strongly disagree to strongly agree | 5 | Agree + Strongly agree vs all other item responses | Neutral responses, if available, were excluded from numerator and retained in denominator | 287 | 350 | 0.8200 | Mapped to P2 (AI perceived as beneficial to medical career) under operational definition: Proportion agreeing that AI will be beneficial, advantageous, or valuable for their future medical career or clinical practice. | Y |
| Alan Yuesheng Xu 2026 | P2 | Having ChatGPT as a resource would help them better manage patient care and make them more effective physicians | Author-reported proportion; response scale not reported in the source article | Not clearly reported | Author-reported proportion; numerator is the endorsed response category | Neutral handling not explicitly reported; denominator retained as reported | 136 | 157 | 0.8662 | Mapped to P2 (AI perceived as beneficial to medical career) under operational definition: Proportion agreeing that AI will be beneficial, advantageous, or valuable for their future medical career or clinical practice. | Y |
| Sumit Prasad 2026 | P2 | AI will be a highly required tool in my field | 5-point Likert agreement scale: strongly disagree to strongly agree | 5 | Agree + Strongly agree vs all other item responses | Neutral responses, if available, were excluded from numerator and retained in denominator | 244 | 315 | 0.7746 | Mapped to P2 (AI perceived as beneficial to medical career) under operational definition: Proportion agreeing that AI will be beneficial, advantageous, or valuable for their future medical career or clinical practice. | Y |
| Imad Chakri 2025 | P2 | AI education will benefit my career | Author-reported proportion; response scale not reported in the source article | Not clearly reported | Author-reported proportion; numerator is the endorsed response category | Neutral handling not explicitly reported; denominator retained as reported | 539 | 580 | 0.9293 | Mapped to P2 (AI perceived as beneficial to medical career) under operational definition: Proportion agreeing that AI will be beneficial, advantageous, or valuable for their future medical career or clinical practice. | Y |
| Bruna Giavina-Bianchi 2025 | P2 | AI would facilitate physicians’ work | Author-reported proportion; response scale not reported in the source article | Not clearly reported | Author-reported proportion; numerator is the endorsed response category | Neutral handling not explicitly reported; denominator retained as reported | 125 | 144 | 0.8681 | Mapped to P2 (AI perceived as beneficial to medical career) under operational definition: Proportion agreeing that AI will be beneficial, advantageous, or valuable for their future medical career or clinical practice. | Y |
| Anna Fußhöller 2025 | P2 | Students more familiar with AI have a career advantage | Author-reported proportion; response scale not reported in the source article | Not clearly reported | Author-reported proportion; numerator is the endorsed response category | Neutral handling not explicitly reported; denominator retained as reported | 52 | 84 | 0.6190 | Mapped to P2 (AI perceived as beneficial to medical career) under operational definition: Proportion agreeing that AI will be beneficial, advantageous, or valuable for their future medical career or clinical practice. | Y |
| Bayan Almehmadi 2025 | P2 | AI enables healthcare professionals to make more accurate decisions | Author-reported proportion; response scale not reported in the source article | Not clearly reported | Author-reported proportion; numerator is the endorsed response category | Neutral response category reported; not counted in numerator and retained in denominator unless source denominator was item-specific | 109 | 170 | 0.6412 | Mapped to P2 (AI perceived as beneficial to medical career) under operational definition: Proportion agreeing that AI will be beneficial, advantageous, or valuable for their future medical career or clinical practice. | Y |
| Muhammad Farooq and Usmani (2025) | P2 | AI will have a significant positive impact on future medical practice/career | Author-reported proportion; response scale not reported in the source article | Not clearly reported | Author-reported proportion; numerator is the endorsed response category | Neutral handling not explicitly reported; denominator retained as reported | 105 | 152 | 0.6908 | Mapped to P2 (AI perceived as beneficial to medical career) under operational definition: Proportion agreeing that AI will be beneficial, advantageous, or valuable for their future medical career or clinical practice. | N |
| Ahmed Hafez Allam 2024 | P2 | Teaching in artificial intelligence will be beneficial for my career (agree/strongly agree) | Likert agreement scale; point count not reported in the source article | Not clearly reported | Agree + Strongly agree vs all other item responses | Neutral responses, if available, were excluded from numerator and retained in denominator | 3183 | 4492 | 0.7086 | Mapped to P2 (AI perceived as beneficial to medical career) under operational definition: Proportion agreeing that AI will be beneficial, advantageous, or valuable for their future medical career or clinical practice. | Y |
| Fahad Abdullah Saeed Alwadani 2024 | P2 | Teaching in artificial intelligence will be beneficial for my career (agree + strongly agree) | 5-point Likert agreement scale: strongly disagree to strongly agree | 5 | Agree + Strongly agree vs all other item responses | Neutral responses, if available, were excluded from numerator and retained in denominator | 213 | 303 | 0.7030 | Mapped to P2 (AI perceived as beneficial to medical career) under operational definition: Proportion agreeing that AI will be beneficial, advantageous, or valuable for their future medical career or clinical practice. | Y |
| Salita Angkurawaranon 2024 | P2 | Teaching in artificial intelligence will be beneficial for my career (agree + strongly agree) | 5-point Likert agreement scale: strongly disagree to strongly agree | 5 | Agree + Strongly agree vs all other item responses | Neutral responses, if available, were excluded from numerator and retained in denominator | 312 | 352 | 0.8864 | Mapped to P2 (AI perceived as beneficial to medical career) under operational definition: Proportion agreeing that AI will be beneficial, advantageous, or valuable for their future medical career or clinical practice. | Y |
| Saif M I Alkhaaldi 2023 | P2 | ChatGPT would expand career opportunities | Author-reported proportion; response scale not reported in the source article | Not clearly reported | Author-reported proportion; numerator is the endorsed response category | Neutral handling not explicitly reported; denominator retained as reported | 78 | 265 | 0.2943 | Mapped to P2 (AI perceived as beneficial to medical career) under operational definition: Proportion agreeing that AI will be beneficial, advantageous, or valuable for their future medical career or clinical practice. | Y |
| Alvin Yong Zong Tung 2023 | P2 | Teaching in AI will be beneficial for my career | Author-reported proportion; response scale not reported in the source article | Not clearly reported | Author-reported proportion; numerator is the endorsed response category | Neutral handling not explicitly reported; denominator retained as reported | 214 | 301 | 0.7110 | Mapped to P2 (AI perceived as beneficial to medical career) under operational definition: Proportion agreeing that AI will be beneficial, advantageous, or valuable for their future medical career or clinical practice. | Y |
| Ali Jasem Buabbas 2023 | P2 | Learning about AI will benefit my career | Author-reported proportion; response scale not reported in the source article | Not clearly reported | Author-reported proportion; numerator is the endorsed response category | Neutral handling not explicitly reported; denominator retained as reported | 329 | 352 | 0.9347 | Mapped to P2 (AI perceived as beneficial to medical career) under operational definition: Proportion agreeing that AI will be beneficial, advantageous, or valuable for their future medical career or clinical practice. | Y |
| David Shalom Liu 2022 | P2 | Training in artificial intelligence concepts during medical school would be useful for their future | 5-point Likert agreement scale: strongly disagree to strongly agree | 5 | Author-reported proportion; numerator is the endorsed response category | Neutral handling not explicitly reported; denominator retained as reported | 355 | 388 | 0.9149 | Mapped to P2 (AI perceived as beneficial to medical career) under operational definition: Proportion agreeing that AI will be beneficial, advantageous, or valuable for their future medical career or clinical practice. | Y |
| Cherry Sit 2020 | P2 | Teaching in AI would be beneficial for their careers | 5-point Likert agreement scale: strongly disagree to strongly agree | 5 | Author-reported proportion; numerator is the endorsed response category | Neutral response category reported; not counted in numerator and retained in denominator unless source denominator was item-specific | 430 | 484 | 0.8884 | Mapped to P2 (AI perceived as beneficial to medical career) under operational definition: Proportion agreeing that AI will be beneficial, advantageous, or valuable for their future medical career or clinical practice. | Y |
| Ali Veysel Kara 2026 | P3 | Desire to receive formal AI-related education during medical school | Author-reported proportion; response scale not reported in the source article | Not clearly reported | Author-reported proportion; numerator is the endorsed response category | Neutral handling not explicitly reported; denominator retained as reported | 1065 | 1346 | 0.7912 | Mapped to P3 (Support for AI integration into medical curriculum) under operational definition: Proportion agreeing that AI should be taught in medical school, integrated into the curriculum, or that they want to receive AI training. | Y |
| Amna Albusmait 2026 | P3 | All medical students should receive teaching in AI (agree + strongly agree) | 5-point Likert agreement scale: strongly disagree to strongly agree | 5 | Agree + Strongly agree vs all other item responses | Neutral responses, if available, were excluded from numerator and retained in denominator | 242 | 350 | 0.6914 | Mapped to P3 (Support for AI integration into medical curriculum) under operational definition: Proportion agreeing that AI should be taught in medical school, integrated into the curriculum, or that they want to receive AI training. | Y |
| Alan Yuesheng Xu 2026 | P3 | Concepts of AI should be taught in medical school | Author-reported proportion; response scale not reported in the source article | Not clearly reported | Author-reported proportion; numerator is the endorsed response category | Neutral handling not explicitly reported; denominator retained as reported | 142 | 177 | 0.8023 | Mapped to P3 (Support for AI integration into medical curriculum) under operational definition: Proportion agreeing that AI should be taught in medical school, integrated into the curriculum, or that they want to receive AI training. | Y |
| Sumit Prasad 2026 | P3 | AI should be a part of the training system among students of medical fields | 5-point Likert agreement scale: strongly disagree to strongly agree | 5 | Agree + Strongly agree vs all other item responses | Neutral responses, if available, were excluded from numerator and retained in denominator | 256 | 316 | 0.8101 | Mapped to P3 (Support for AI integration into medical curriculum) under operational definition: Proportion agreeing that AI should be taught in medical school, integrated into the curriculum, or that they want to receive AI training. | Y |
| Omar A. Ghanem 2025 | P3 | Healthcare students should learn the basics of AI | Author-reported proportion; response scale not reported in the source article | Not clearly reported | Author-reported proportion; numerator is the endorsed response category | Neutral handling not explicitly reported; denominator retained as reported | 314 | 423 | 0.7423 | Mapped to P3 (Support for AI integration into medical curriculum) under operational definition: Proportion agreeing that AI should be taught in medical school, integrated into the curriculum, or that they want to receive AI training. | Y |
| Imad Chakri 2025 | P3 | AI should be part of medical education | Author-reported proportion; response scale not reported in the source article | Not clearly reported | Author-reported proportion; numerator is the endorsed response category | Neutral handling not explicitly reported; denominator retained as reported | 522 | 580 | 0.9000 | Mapped to P3 (Support for AI integration into medical curriculum) under operational definition: Proportion agreeing that AI should be taught in medical school, integrated into the curriculum, or that they want to receive AI training. | Y |
| Anna Fußhöller 2025 | P3 | Would appreciate more computer science courses to learn AI in medicine | Author-reported proportion; response scale not reported in the source article | Not clearly reported | Author-reported proportion; numerator is the endorsed response category | Neutral handling not explicitly reported; denominator retained as reported | 60 | 84 | 0.7143 | Mapped to P3 (Support for AI integration into medical curriculum) under operational definition: Proportion agreeing that AI should be taught in medical school, integrated into the curriculum, or that they want to receive AI training. | Y |
| Cecilia Tran 2025 | P3 | Generative AI should be implemented as a resource or taught in UGME/PGME curriculum | Author-reported proportion; response scale not reported in the source article | Not clearly reported | Author-reported proportion; numerator is the endorsed response category | Neutral handling not explicitly reported; denominator retained as reported | 126 | 164 | 0.7683 | Mapped to P3 (Support for AI integration into medical curriculum) under operational definition: Proportion agreeing that AI should be taught in medical school, integrated into the curriculum, or that they want to receive AI training. | Y |
| Zahra Arab-Borzu 2025 | P3 | AI should be included in my health education | Author-reported proportion; response scale not reported in the source article | Not clearly reported | Author-reported proportion; numerator is the endorsed response category | Neutral handling not explicitly reported; denominator retained as reported | 211 | 279 | 0.7563 | Mapped to P3 (Support for AI integration into medical curriculum) under operational definition: Proportion agreeing that AI should be taught in medical school, integrated into the curriculum, or that they want to receive AI training. | Y |
| Samah Alfahl 2025 | P3 | Healthcare students should learn about AI | 5-point Likert agreement scale: strongly disagree to strongly agree | 5 | Author-reported proportion; numerator is the endorsed response category | Neutral handling not explicitly reported; denominator retained as reported | 130 | 189 | 0.6878 | Mapped to P3 (Support for AI integration into medical curriculum) under operational definition: Proportion agreeing that AI should be taught in medical school, integrated into the curriculum, or that they want to receive AI training. | Y |
| Min Zhang 2025 | P3 | Support for integration of AILMs in current education | 5-point Likert agreement scale: strongly disagree to strongly agree | 5 | Dichotomization method not reported in primary study; proportion extracted from reported percentage | Neutral response category reported; not counted in numerator and retained in denominator unless source denominator was item-specific | 187 | 254 | 0.7362 | Mapped to P3 (Support for AI integration into medical curriculum) under operational definition: Proportion agreeing that AI should be taught in medical school, integrated into the curriculum, or that they want to receive AI training. | Y |
| Abdul Sami 2025 | P3 | Importance of integrating AI into medical education/creating dedicated AI tools | Author-reported proportion; response scale not reported in the source article | Not clearly reported | Author-reported proportion; numerator is the endorsed response category | Neutral handling not explicitly reported; denominator retained as reported | 562 | 702 | 0.8006 | Mapped to P3 (Support for AI integration into medical curriculum) under operational definition: Proportion agreeing that AI should be taught in medical school, integrated into the curriculum, or that they want to receive AI training. | N |
| Poorna Fernando 2025 | P3 | AI should be formally included in the medical curriculum | Author-reported proportion; response scale not reported in the source article | Not clearly reported | Author-reported proportion; numerator is the endorsed response category | Neutral handling not explicitly reported; denominator retained as reported | 258 | 304 | 0.8487 | Mapped to P3 (Support for AI integration into medical curriculum) under operational definition: Proportion agreeing that AI should be taught in medical school, integrated into the curriculum, or that they want to receive AI training. | Y |
| Aleksandra Ignjatović 2025 | P3 | AI should be included in medical school curricula and specialization programs (among nonusers) | Author-reported proportion; response scale not reported in the source article | Not clearly reported | Author-reported proportion; numerator is the endorsed response category | Neutral handling not explicitly reported; denominator retained as reported | 257 | 701 | 0.3666 | Mapped to P3 (Support for AI integration into medical curriculum) under operational definition: Proportion agreeing that AI should be taught in medical school, integrated into the curriculum, or that they want to receive AI training. | Y |
| Emre Sanri 2025 | P3 | Advocated broader integration of AI into the curriculum | Author-reported proportion; response scale not reported in the source article | Not clearly reported | Author-reported proportion; numerator is the endorsed response category | Neutral handling not explicitly reported; denominator retained as reported | 99 | 120 | 0.8250 | Mapped to P3 (Support for AI integration into medical curriculum) under operational definition: Proportion agreeing that AI should be taught in medical school, integrated into the curriculum, or that they want to receive AI training. | Y |
| M Hassaan Shah 2025 | P3 | Support integration of AI with traditional teaching methods | Author-reported proportion; response scale not reported in the source article | Not clearly reported | Author-reported proportion; numerator is the endorsed response category | Neutral response category reported; not counted in numerator and retained in denominator unless source denominator was item-specific | 156 | 200 | 0.7800 | Mapped to P3 (Support for AI integration into medical curriculum) under operational definition: Proportion agreeing that AI should be taught in medical school, integrated into the curriculum, or that they want to receive AI training. | Y |
| Mickaël Ringeval 2025 | P3 | Agreed that dHealth education should be a mandatory component of medical training | 5-point Likert agreement scale: strongly disagree to strongly agree | 5 | Author-reported proportion; numerator is the endorsed response category | Neutral handling not explicitly reported; denominator retained as reported | 306 | 361 | 0.8476 | Mapped to P3 (Support for AI integration into medical curriculum) under operational definition: Proportion agreeing that AI should be taught in medical school, integrated into the curriculum, or that they want to receive AI training. | Y |
| Seyyedeh Fatemeh Mousavi Baigi 2025 | P3 | AI fundamentals should be taught to healthcare students (scores 6–10 on 0–10 scale) | Likert agreement scale; point count not reported in the source article | Not clearly reported | Dichotomization method not reported in primary study; proportion extracted from reported percentage | Neutral handling not explicitly reported; denominator retained as reported | 75 | 95 | 0.7895 | Mapped to P3 (Support for AI integration into medical curriculum) under operational definition: Proportion agreeing that AI should be taught in medical school, integrated into the curriculum, or that they want to receive AI training. | Y |
| Robbi Miguel G. Falcon 2025 | P3 | Agreement with inclusion of AI-related topics in the medical curriculum | 5-point Likert agreement scale: strongly disagree to strongly agree | 5 | Author-reported proportion; numerator is the endorsed response category | Neutral handling not explicitly reported; denominator retained as reported | 20 | 20 | 1.0000 | Mapped to P3 (Support for AI integration into medical curriculum) under operational definition: Proportion agreeing that AI should be taught in medical school, integrated into the curriculum, or that they want to receive AI training. | Y |
| Muhammad Farooq and Usmani (2025) | P3 | AI-related skills should be mandatory in the medical education curriculum | Author-reported proportion; response scale not reported in the source article | Not clearly reported | Author-reported proportion; numerator is the endorsed response category | Neutral handling not explicitly reported; denominator retained as reported | 90 | 152 | 0.5921 | Mapped to P3 (Support for AI integration into medical curriculum) under operational definition: Proportion agreeing that AI should be taught in medical school, integrated into the curriculum, or that they want to receive AI training. | N |
| Ahmed Hafez Allam 2024 | P3 | All medical students should receive teaching in artificial intelligence (agree/strongly agree) | Likert agreement scale; point count not reported in the source article | Not clearly reported | Agree + Strongly agree vs all other item responses | Neutral responses, if available, were excluded from numerator and retained in denominator | 3132 | 4492 | 0.6972 | Mapped to P3 (Support for AI integration into medical curriculum) under operational definition: Proportion agreeing that AI should be taught in medical school, integrated into the curriculum, or that they want to receive AI training. | Y |
| Kamel Jebreen 2024 | P3 | Confirmed a dire need to include AI training in medical curricula | Likert agreement scale; point count not reported in the source article | Not clearly reported | Author-reported proportion; numerator is the endorsed response category | Neutral handling not explicitly reported; denominator retained as reported | 247 | 349 | 0.7077 | Mapped to P3 (Support for AI integration into medical curriculum) under operational definition: Proportion agreeing that AI should be taught in medical school, integrated into the curriculum, or that they want to receive AI training. | Y |
| Fahad Abdullah Saeed Alwadani 2024 | P3 | All medical students should receive teaching in artificial intelligence (agree + strongly agree) | 5-point Likert agreement scale: strongly disagree to strongly agree | 5 | Agree + Strongly agree vs all other item responses | Neutral responses, if available, were excluded from numerator and retained in denominator | 179 | 303 | 0.5908 | Mapped to P3 (Support for AI integration into medical curriculum) under operational definition: Proportion agreeing that AI should be taught in medical school, integrated into the curriculum, or that they want to receive AI training. | Y |
| Zainudheen Faroog 2024 | P3 | AI should be integrated into ophthalmology residency/fellowship training (agree) | response scale not reported in the source article | Not reported | Dichotomization method not reported in primary study; proportion extracted from reported percentage | Neutral response category reported; not counted in numerator and retained in denominator unless source denominator was item-specific | 332 | 345 | 0.9623 | Mapped to P3 (Support for AI integration into medical curriculum) under operational definition: Proportion agreeing that AI should be taught in medical school, integrated into the curriculum, or that they want to receive AI training. | Y |
| Preetha Jackson 2024 | P3 | Demand for structured AI training on reducing medical errors | Author-reported proportion; response scale not reported in the source article | Not clearly reported | Author-reported proportion; numerator is the endorsed response category | Neutral handling not explicitly reported; denominator retained as reported | 250 | 325 | 0.7692 | Mapped to P3 (Support for AI integration into medical curriculum) under operational definition: Proportion agreeing that AI should be taught in medical school, integrated into the curriculum, or that they want to receive AI training. | Y |
| Salita Angkurawaranon 2024 | P3 | All medical students should receive teaching in artificial intelligence (agree + strongly agree) | 5-point Likert agreement scale: strongly disagree to strongly agree | 5 | Agree + Strongly agree vs all other item responses | Neutral responses, if available, were excluded from numerator and retained in denominator | 271 | 352 | 0.7699 | Mapped to P3 (Support for AI integration into medical curriculum) under operational definition: Proportion agreeing that AI should be taught in medical school, integrated into the curriculum, or that they want to receive AI training. | Y |
| Omar A. Daher 2024 | P3 | All medical students should receive teaching in AI | 5-point Likert agreement scale: strongly disagree to strongly agree | 5 | Author-reported proportion; numerator is the endorsed response category | Neutral handling not explicitly reported; denominator retained as reported | 351 | 365 | 0.9616 | Mapped to P3 (Support for AI integration into medical curriculum) under operational definition: Proportion agreeing that AI should be taught in medical school, integrated into the curriculum, or that they want to receive AI training. | Y |
| Lukas Weidener 2024 | P3 | S13: AI should be part of medical education (agree + strongly agree) | 5-point Likert agreement scale: strongly disagree to strongly agree | 5 | Agree + Strongly agree vs all other item responses | Neutral responses, if available, were excluded from numerator and retained in denominator | 365 | 487 | 0.7495 | Mapped to P3 (Support for AI integration into medical curriculum) under operational definition: Proportion agreeing that AI should be taught in medical school, integrated into the curriculum, or that they want to receive AI training. | Y |
| Jason Luong 2025 | P3 | AI education should be part of medical training (agree + strongly agree) | 5-point Likert agreement scale: strongly disagree to strongly agree | 5 | Agree + Strongly agree vs all other item responses | Neutral responses, if available, were excluded from numerator and retained in denominator | 92 | 220 | 0.4182 | Mapped to P3 (Support for AI integration into medical curriculum) under operational definition: Proportion agreeing that AI should be taught in medical school, integrated into the curriculum, or that they want to receive AI training. | Y |
| Alan Y. Xu 2024 | P3 | All respondents believed AI should be taught in the medical curriculum | response scale not reported in the source article | Not reported | Dichotomization method not reported in primary study; proportion extracted from reported percentage | Neutral handling not explicitly reported; denominator retained as reported | 102 | 102 | 1.0000 | Mapped to P3 (Support for AI integration into medical curriculum) under operational definition: Proportion agreeing that AI should be taught in medical school, integrated into the curriculum, or that they want to receive AI training. | Y |
| Khaldoon Al-Roomi 2024 | P3 | Do you think that medical schools should use artificial intelligence (AI) in their curricula' | response scale not reported in the source article | Not reported | Dichotomization method not reported in primary study; proportion extracted from reported percentage | Neutral response category reported; not counted in numerator and retained in denominator unless source denominator was item-specific | 104 | 152 | 0.6842 | Mapped to P3 (Support for AI integration into medical curriculum) under operational definition: Proportion agreeing that AI should be taught in medical school, integrated into the curriculum, or that they want to receive AI training. | Y |
| Alvin Yong Zong Tung 2023 | P3 | All medical students should receive teaching in AI | 5-point Likert agreement scale: strongly disagree to strongly agree | 5 | Author-reported proportion; numerator is the endorsed response category | Neutral handling not explicitly reported; denominator retained as reported | 209 | 301 | 0.6944 | Mapped to P3 (Support for AI integration into medical curriculum) under operational definition: Proportion agreeing that AI should be taught in medical school, integrated into the curriculum, or that they want to receive AI training. | Y |
| Jonathon Stewart 2023 | P3 | Artificial intelligence education should be part of medical training | response scale not reported in the source article | Not reported | Dichotomization method not reported in primary study; proportion extracted from reported percentage | Neutral handling not explicitly reported; denominator retained as reported | 79 | 134 | 0.5896 | Mapped to P3 (Support for AI integration into medical curriculum) under operational definition: Proportion agreeing that AI should be taught in medical school, integrated into the curriculum, or that they want to receive AI training. | Y |
| Ali Jasem Buabbas 2023 | P3 | Medical students should receive AI teaching or training | Author-reported proportion; response scale not reported in the source article | Not clearly reported | Author-reported proportion; numerator is the endorsed response category | Neutral handling not explicitly reported; denominator retained as reported | 289 | 352 | 0.8210 | Mapped to P3 (Support for AI integration into medical curriculum) under operational definition: Proportion agreeing that AI should be taught in medical school, integrated into the curriculum, or that they want to receive AI training. | Y |
| Andrés Barreiro-Ares 2023 | P3 | Academic training in AI was valued positively | 5-point Likert agreement scale: strongly disagree to strongly agree | 5 | Author-reported proportion; numerator is the endorsed response category | Neutral handling not explicitly reported; denominator retained as reported | 225 | 281 | 0.8007 | Mapped to P3 (Support for AI integration into medical curriculum) under operational definition: Proportion agreeing that AI should be taught in medical school, integrated into the curriculum, or that they want to receive AI training. | Y |
| G. Caparrós Galán 2022 | P3 | Basic AI training should be included in the medical curriculum | Author-reported proportion; response scale not reported in the source article | Not clearly reported | Author-reported proportion; numerator is the endorsed response category | Neutral handling not explicitly reported; denominator retained as reported | 211 | 341 | 0.6188 | Mapped to P3 (Support for AI integration into medical curriculum) under operational definition: Proportion agreeing that AI should be taught in medical school, integrated into the curriculum, or that they want to receive AI training. | Y |
| Aidan Pucchio 2022 | P3 | Artificial intelligence should be a formally taught topic in medical education | Author-reported proportion; response scale not reported in the source article | Not clearly reported | Author-reported proportion; numerator is the endorsed response category | Neutral handling not explicitly reported; denominator retained as reported | 304 | 455 | 0.6681 | Mapped to P3 (Support for AI integration into medical curriculum) under operational definition: Proportion agreeing that AI should be taught in medical school, integrated into the curriculum, or that they want to receive AI training. | Y |
| Charlotte Blease 2022 | P3 | Discussion about artificial intelligence/machine learning should be part of medical training | response scale not reported in the source article | Not reported | Dichotomization method not reported in primary study; proportion extracted from reported percentage | Neutral handling not explicitly reported; denominator retained as reported | 190 | 242 | 0.7851 | Mapped to P3 (Support for AI integration into medical curriculum) under operational definition: Proportion agreeing that AI should be taught in medical school, integrated into the curriculum, or that they want to receive AI training. | Y |
| Cherry Sit 2020 | P3 | All medical students should receive training in AI as part of their medical degree | 5-point Likert agreement scale: strongly disagree to strongly agree | 5 | Author-reported proportion; numerator is the endorsed response category | Neutral response category reported; not counted in numerator and retained in denominator unless source denominator was item-specific | 378 | 484 | 0.7810 | Mapped to P3 (Support for AI integration into medical curriculum) under operational definition: Proportion agreeing that AI should be taught in medical school, integrated into the curriculum, or that they want to receive AI training. | Y |
| D. Pinto dos Santos 2019 | P3 | Artificial intelligence should be part of medical training (agree entirely + rather agree) | 4-point Likert agreement scale | 4 | Author-reported proportion; numerator is the endorsed response category | Neutral handling not explicitly reported; denominator retained as reported | 187 | 263 | 0.7110 | Mapped to P3 (Support for AI integration into medical curriculum) under operational definition: Proportion agreeing that AI should be taught in medical school, integrated into the curriculum, or that they want to receive AI training. | Y |
| Xi Cao 2026 | P4 | Do you think GenAI can replace healthcare professionals in clinical decision-making' (completely agree + partially agree) | Author-reported proportion; response scale not reported in the source article | Not clearly reported | Completely agree + Partially agree vs all other item responses | Neutral responses, if available, were excluded from numerator and retained in denominator | 461 | 1062 | 0.4341 | Mapped to P4 (Concern about physician replacement by AI) under operational definition: Proportion expressing concern, worry, or belief that AI could/will replace physicians or specific medical specialties. | Y |
| Amna Albusmait 2026 | P4 | Some specialties will be replaced by AI within their lifetime (agree + strongly agree) | 5-point Likert agreement scale: strongly disagree to strongly agree | 5 | Agree + Strongly agree vs all other item responses | Neutral responses, if available, were excluded from numerator and retained in denominator | 164 | 350 | 0.4686 | Mapped to P4 (Concern about physician replacement by AI) under operational definition: Proportion expressing concern, worry, or belief that AI could/will replace physicians or specific medical specialties. | Y |
| Alan Yuesheng Xu 2026 | P4 | AI would lead to a reduction in physician jobs | Author-reported proportion; response scale not reported in the source article | Not clearly reported | Author-reported proportion; numerator is the endorsed response category | Neutral handling not explicitly reported; denominator retained as reported | 63 | 177 | 0.3559 | Mapped to P4 (Concern about physician replacement by AI) under operational definition: Proportion expressing concern, worry, or belief that AI could/will replace physicians or specific medical specialties. | Y |
| Sumit Prasad 2026 | P4 | Some specialties are more prone to being replaced by AI than others | 5-point Likert agreement scale: strongly disagree to strongly agree | 5 | Agree + Strongly agree vs all other item responses | Neutral responses, if available, were excluded from numerator and retained in denominator | 180 | 315 | 0.5714 | Mapped to P4 (Concern about physician replacement by AI) under operational definition: Proportion expressing concern, worry, or belief that AI could/will replace physicians or specific medical specialties. | Y |
| Yacoub A. Yousef 2026 | P4 | AI may replace ophthalmologists’ roles | Author-reported proportion; response scale not reported in the source article | Not clearly reported | Author-reported proportion; numerator is the endorsed response category | Neutral handling not explicitly reported; denominator retained as reported | 105 | 353 | 0.2975 | Mapped to P4 (Concern about physician replacement by AI) under operational definition: Proportion expressing concern, worry, or belief that AI could/will replace physicians or specific medical specialties. | Y |
| Omar A. Ghanem 2025 | P4 | Some specialties are more prone to be replaced by AI than others | Author-reported proportion; response scale not reported in the source article | Not clearly reported | Author-reported proportion; numerator is the endorsed response category | Neutral handling not explicitly reported; denominator retained as reported | 216 | 423 | 0.5106 | Mapped to P4 (Concern about physician replacement by AI) under operational definition: Proportion expressing concern, worry, or belief that AI could/will replace physicians or specific medical specialties. | Y |
| Imad Chakri 2025 | P4 | Certain specialties could be supplanted by AI | Author-reported proportion; response scale not reported in the source article | Not clearly reported | Author-reported proportion; numerator is the endorsed response category | Neutral handling not explicitly reported; denominator retained as reported | 331 | 580 | 0.5707 | Mapped to P4 (Concern about physician replacement by AI) under operational definition: Proportion expressing concern, worry, or belief that AI could/will replace physicians or specific medical specialties. | Y |
| Bruna Giavina-Bianchi 2025 | P4 | AI will totally or partially replace medical imaging work | Author-reported proportion; response scale not reported in the source article | Not clearly reported | Author-reported proportion; numerator is the endorsed response category | Neutral handling not explicitly reported; denominator retained as reported | 43 | 145 | 0.2966 | Mapped to P4 (Concern about physician replacement by AI) under operational definition: Proportion expressing concern, worry, or belief that AI could/will replace physicians or specific medical specialties. | Y |
| Bayan Almehmadi 2025 | P4 | AI will replace the physician in the healthcare system | Author-reported proportion; response scale not reported in the source article | Not clearly reported | Author-reported proportion; numerator is the endorsed response category | Neutral handling not explicitly reported; denominator retained as reported | 36 | 170 | 0.2118 | Mapped to P4 (Concern about physician replacement by AI) under operational definition: Proportion expressing concern, worry, or belief that AI could/will replace physicians or specific medical specialties. | Y |
| Zahra Arab-Borzu 2025 | P4 | In the future, some healthcare professions may be replaced by AI | 5-point Likert agreement scale: strongly disagree to strongly agree | 5 | Dichotomization method not reported in primary study; proportion extracted from reported percentage | Neutral handling not explicitly reported; denominator retained as reported | 214 | 278 | 0.7698 | Mapped to P4 (Concern about physician replacement by AI) under operational definition: Proportion expressing concern, worry, or belief that AI could/will replace physicians or specific medical specialties. | Y |
| Samah Alfahl 2025 | P4 | AI may replace certain medical specialties (closest reported paraphrase; exact source wording not verified) | 5-point Likert agreement scale: strongly disagree to strongly agree | 5 | Author-reported proportion; numerator is the endorsed response category | Neutral handling not explicitly reported; denominator retained as reported | 88 | 189 | 0.4656 | Mapped to P4 (Concern about physician replacement by AI) under operational definition: Proportion expressing concern, worry, or belief that AI could/will replace physicians or specific medical specialties. | Y |
| Afnan Almuhanna 2025 | P4 | Concern that AI may replace radiologists | Likert agreement scale; point count not reported in the source article | Not clearly reported | Agree + Strongly agree vs all other item responses | Neutral responses, if available, were excluded from numerator and retained in denominator | 69 | 292 | 0.2363 | Mapped to P4 (Concern about physician replacement by AI) under operational definition: Proportion expressing concern, worry, or belief that AI could/will replace physicians or specific medical specialties. | Y |
| Abdul Sami 2025 | P4 | Fear that AI could replace human medical professionals | 5-point Likert agreement scale: strongly disagree to strongly agree | 5 | Author-reported proportion; numerator is the endorsed response category | Neutral handling not explicitly reported; denominator retained as reported | 314 | 702 | 0.4473 | Mapped to P4 (Concern about physician replacement by AI) under operational definition: Proportion expressing concern, worry, or belief that AI could/will replace physicians or specific medical specialties. | N |
| Mingchan Liu 2026 | P4 | Concern that AI development may lead to job substitution issues (agree + strongly agree) | Likert agreement scale; point count not reported in the source article | Not clearly reported | Agree + Strongly agree vs all other item responses | Neutral responses, if available, were excluded from numerator and retained in denominator | 423 | 788 | 0.5368 | Mapped to P4 (Concern about physician replacement by AI) under operational definition: Proportion expressing concern, worry, or belief that AI could/will replace physicians or specific medical specialties. | Y |
| Aleksandra Ignjatović 2025 | P4 | ChatGPT will threaten people's jobs! | response scale not reported in the source article | Not reported | Dichotomization method not reported in primary study; proportion extracted from reported percentage | Neutral handling not explicitly reported; denominator retained as reported | 258 | 511 | 0.5049 | Mapped to P4 (Concern about physician replacement by AI) under operational definition: Proportion expressing concern, worry, or belief that AI could/will replace physicians or specific medical specialties. | Y |
| Muhammad Farooq and Usmani (2025) | P4 | Concern about potential job displacement due to AI | Author-reported proportion; response scale not reported in the source article | Not clearly reported | Author-reported proportion; numerator is the endorsed response category | Neutral handling not explicitly reported; denominator retained as reported | 78 | 152 | 0.5132 | Mapped to P4 (Concern about physician replacement by AI) under operational definition: Proportion expressing concern, worry, or belief that AI could/will replace physicians or specific medical specialties. | N |
| Ahmed Hafez Allam 2024 | P4 | The human non-interventional physician will be replaced in the foreseeable future (agree/strongly agree) | Likert agreement scale; point count not reported in the source article | Not clearly reported | Agree + Strongly agree vs all other item responses | Neutral responses, if available, were excluded from numerator and retained in denominator | 645 | 4492 | 0.1436 | Mapped to P4 (Concern about physician replacement by AI) under operational definition: Proportion expressing concern, worry, or belief that AI could/will replace physicians or specific medical specialties. | Y |
| Fahad Abdullah Saeed Alwadani 2024 | P4 | Some specialties will be replaced by AI during my lifetime (agree + strongly agree) | 5-point Likert agreement scale: strongly disagree to strongly agree | 5 | Agree + Strongly agree vs all other item responses | Neutral responses, if available, were excluded from numerator and retained in denominator | 139 | 303 | 0.4587 | Mapped to P4 (Concern about physician replacement by AI) under operational definition: Proportion expressing concern, worry, or belief that AI could/will replace physicians or specific medical specialties. | Y |
| Zainudheen Faroog 2024 | P4 | AI in ophthalmology will replace ophthalmologists (agree) | response scale not reported in the source article | Not reported | Dichotomization method not reported in primary study; proportion extracted from reported percentage | Neutral response category reported; not counted in numerator and retained in denominator unless source denominator was item-specific | 49 | 345 | 0.1420 | Mapped to P4 (Concern about physician replacement by AI) under operational definition: Proportion expressing concern, worry, or belief that AI could/will replace physicians or specific medical specialties. | Y |
| Mohammed Hammad Jaber Amin 2024 | P4 | AI will replace physicians in the future (agree + strongly agree) | Likert agreement scale; point count not reported in the source article | Not clearly reported | Agree + Strongly agree vs all other item responses | Neutral responses, if available, were excluded from numerator and retained in denominator | 240 | 762 | 0.3150 | Mapped to P4 (Concern about physician replacement by AI) under operational definition: Proportion expressing concern, worry, or belief that AI could/will replace physicians or specific medical specialties. | Y |
| Preetha Jackson 2024 | P4 | Concern that AI may replace physicians | 5-point Likert agreement scale: strongly disagree to strongly agree | 5 | Author-reported proportion; numerator is the endorsed response category | Neutral handling not explicitly reported; denominator retained as reported | 122 | 325 | 0.3754 | Mapped to P4 (Concern about physician replacement by AI) under operational definition: Proportion expressing concern, worry, or belief that AI could/will replace physicians or specific medical specialties. | Y |
| Salita Angkurawaranon 2024 | P4 | Some specialties will be replaced by artificial intelligence during my lifetime (agree + strongly agree) | 5-point Likert agreement scale: strongly disagree to strongly agree | 5 | Agree + Strongly agree vs all other item responses | Neutral responses, if available, were excluded from numerator and retained in denominator | 159 | 352 | 0.4517 | Mapped to P4 (Concern about physician replacement by AI) under operational definition: Proportion expressing concern, worry, or belief that AI could/will replace physicians or specific medical specialties. | Y |
| Omar A. Daher 2024 | P4 | General physicians will be replaced in the foreseeable future (agree) | 5-point Likert agreement scale: strongly disagree to strongly agree | 5 | Author-reported proportion; numerator is the endorsed response category | Neutral handling not explicitly reported; denominator retained as reported | 90 | 365 | 0.2466 | Mapped to P4 (Concern about physician replacement by AI) under operational definition: Proportion expressing concern, worry, or belief that AI could/will replace physicians or specific medical specialties. | Y |
| Lukas Weidener 2024 | P4 | S4: reduce the number of jobs for medical staff (agree + strongly agree) | 5-point Likert agreement scale: strongly disagree to strongly agree | 5 | Agree + Strongly agree vs all other item responses | Neutral responses, if available, were excluded from numerator and retained in denominator | 167 | 487 | 0.3429 | Mapped to P4 (Concern about physician replacement by AI) under operational definition: Proportion expressing concern, worry, or belief that AI could/will replace physicians or specific medical specialties. | Y |
| Dalia M. Alammari 2024 | P4 | AI will replace traditional doctor roles | response scale not reported in the source article | Not reported | Dichotomization method not reported in primary study; proportion extracted from reported percentage | Neutral handling not explicitly reported; denominator retained as reported | 71 | 207 | 0.3430 | Mapped to P4 (Concern about physician replacement by AI) under operational definition: Proportion expressing concern, worry, or belief that AI could/will replace physicians or specific medical specialties. | Y |
| Kehinde Sunday Oluwadiya 2023 | P4 | AI could render physicians redundant | Author-reported proportion; response scale not reported in the source article | Not clearly reported | Author-reported proportion; numerator is the endorsed response category | Neutral handling not explicitly reported; denominator retained as reported | 408 | 708 | 0.5763 | Mapped to P4 (Concern about physician replacement by AI) under operational definition: Proportion expressing concern, worry, or belief that AI could/will replace physicians or specific medical specialties. | Y |
| Alvin Yong Zong Tung 2023 | P4 | Some specialties will be replaced by AI during my lifetime | 5-point Likert agreement scale: strongly disagree to strongly agree | 5 | Author-reported proportion; numerator is the endorsed response category | Neutral response category reported; not counted in numerator and retained in denominator unless source denominator was item-specific | 180 | 301 | 0.5980 | Mapped to P4 (Concern about physician replacement by AI) under operational definition: Proportion expressing concern, worry, or belief that AI could/will replace physicians or specific medical specialties. | Y |
| Zinah A. Al Hadithy 2023 | P4 | Every medical trainee should receive training on AI competencies | Author-reported proportion; response scale not reported in the source article | Not clearly reported | Author-reported proportion; numerator is the endorsed response category | Neutral handling not explicitly reported; denominator retained as reported | 174 | 221 | 0.7873 | Mapped to P4 (Concern about physician replacement by AI) under operational definition: Proportion expressing concern, worry, or belief that AI could/will replace physicians or specific medical specialties. | Y |
| George Doumat 2022 | P4 | Some human specialists will be replaced by AI in the near future | Author-reported proportion; response scale not reported in the source article | Not clearly reported | Author-reported proportion; numerator is the endorsed response category | Neutral handling not explicitly reported; denominator retained as reported | 115 | 206 | 0.5583 | Mapped to P4 (Concern about physician replacement by AI) under operational definition: Proportion expressing concern, worry, or belief that AI could/will replace physicians or specific medical specialties. | Y |
| David Shalom Liu 2022 | P4 | Less likely to choose certain specialties because of the anticipated integration of AI | 5-point Likert agreement scale: strongly disagree to strongly agree | 5 | Agree + Somewhat agree + Strongly agree vs all other item responses | Neutral responses, if available, were excluded from numerator and retained in denominator | 70 | 386 | 0.1813 | Mapped to P4 (Concern about physician replacement by AI) under operational definition: Proportion expressing concern, worry, or belief that AI could/will replace physicians or specific medical specialties. | Y |
| G. Caparrós Galán 2022 | P4 | Concern about the role of AI when choosing radiology as a specialty | Author-reported proportion; response scale not reported in the source article | Not clearly reported | Author-reported proportion; numerator is the endorsed response category | Neutral handling not explicitly reported; denominator retained as reported | 125 | 341 | 0.3666 | Mapped to P4 (Concern about physician replacement by AI) under operational definition: Proportion expressing concern, worry, or belief that AI could/will replace physicians or specific medical specialties. | Y |
| Aidan Pucchio 2022 | P4 | In the future some or all physicians will be replaced by AI | Author-reported proportion; response scale not reported in the source article | Not clearly reported | Author-reported proportion; numerator is the endorsed response category | Neutral handling not explicitly reported; denominator retained as reported | 94 | 461 | 0.2039 | Mapped to P4 (Concern about physician replacement by AI) under operational definition: Proportion expressing concern, worry, or belief that AI could/will replace physicians or specific medical specialties. | Y |
| Cherry Sit 2020 | P4 | Some specialties will be replaced by AI within their lifetime | 5-point Likert agreement scale: strongly disagree to strongly agree | 5 | Agree + Strongly agree vs all other item responses | Neutral responses, if available, were excluded from numerator and retained in denominator | 234 | 484 | 0.4835 | Mapped to P4 (Concern about physician replacement by AI) under operational definition: Proportion expressing concern, worry, or belief that AI could/will replace physicians or specific medical specialties. | Y |
| D. Pinto dos Santos 2019 | P4 | The human radiologist will be replaced in the foreseeable future (agree entirely + rather agree) | 4-point Likert agreement scale | 4 | Author-reported proportion; numerator is the endorsed response category | Neutral handling not explicitly reported; denominator retained as reported | 40 | 263 | 0.1521 | Mapped to P4 (Concern about physician replacement by AI) under operational definition: Proportion expressing concern, worry, or belief that AI could/will replace physicians or specific medical specialties. | Y |
| Ali Veysel Kara 2026 | P5 | Aware of AI in medicine | Author-reported proportion; response scale not reported in the source article | Not clearly reported | Author-reported proportion; numerator is the endorsed response category | Neutral handling not explicitly reported; denominator retained as reported | 1092 | 1346 | 0.8113 | Mapped to P5 (Self-reported familiarity or knowledge of AI) under operational definition: Proportion reporting familiarity, knowledge, understanding, or competence with AI concepts/tools. | Y |
| Amna Albusmait 2026 | P5 | I have an understanding of the basic computational principles of AI (agree + strongly agree) | 5-point Likert agreement scale: strongly disagree to strongly agree | 5 | Agree + Strongly agree vs all other item responses | Neutral responses, if available, were excluded from numerator and retained in denominator | 213 | 350 | 0.6086 | Mapped to P5 (Self-reported familiarity or knowledge of AI) under operational definition: Proportion reporting familiarity, knowledge, understanding, or competence with AI concepts/tools. | Y |
| Alan Yuesheng Xu 2026 | P5 | Current understanding of AI rated moderate or advanced | Author-reported proportion; response scale not reported in the source article | Not clearly reported | Author-reported proportion; numerator is the endorsed response category | Neutral handling not explicitly reported; denominator retained as reported | 128 | 177 | 0.7232 | Mapped to P5 (Self-reported familiarity or knowledge of AI) under operational definition: Proportion reporting familiarity, knowledge, understanding, or competence with AI concepts/tools. | Y |
| Sumit Prasad 2026 | P5 | Knowledge of the basics of AI | Author-reported proportion; response scale not reported in the source article | Not clearly reported | Yes/endorsed category vs No/non-endorsed category | No neutral option reported; denominator retained as reported | 239 | 317 | 0.7539 | Mapped to P5 (Self-reported familiarity or knowledge of AI) under operational definition: Proportion reporting familiarity, knowledge, understanding, or competence with AI concepts/tools. | Y |
| Omar A. Ghanem 2025 | P5 | Solid knowledge of the basics of AI | Author-reported proportion; response scale not reported in the source article | Not clearly reported | Author-reported proportion; numerator is the endorsed response category | Neutral handling not explicitly reported; denominator retained as reported | 192 | 423 | 0.4539 | Mapped to P5 (Self-reported familiarity or knowledge of AI) under operational definition: Proportion reporting familiarity, knowledge, understanding, or competence with AI concepts/tools. | Y |
| Weam Mohamed Meargni Ahmed 2025 | P5 | Aware of ChatGPT before the study | Author-reported proportion; response scale not reported in the source article | Not clearly reported | Author-reported proportion; numerator is the endorsed response category | Neutral handling not explicitly reported; denominator retained as reported | 949 | 1443 | 0.6577 | Mapped to P5 (Self-reported familiarity or knowledge of AI) under operational definition: Proportion reporting familiarity, knowledge, understanding, or competence with AI concepts/tools. | Y |
| Imad Chakri 2025 | P5 | Understood AI functioning | Author-reported proportion; response scale not reported in the source article | Not clearly reported | Author-reported proportion; numerator is the endorsed response category | Neutral handling not explicitly reported; denominator retained as reported | 64 | 580 | 0.1103 | Mapped to P5 (Self-reported familiarity or knowledge of AI) under operational definition: Proportion reporting familiarity, knowledge, understanding, or competence with AI concepts/tools. | Y |
| Bruna Giavina-Bianchi 2025 | P5 | Intermediate or high self-assessed knowledge of AI | Author-reported proportion; response scale not reported in the source article | Not clearly reported | Author-reported proportion; numerator is the endorsed response category | Neutral handling not explicitly reported; denominator retained as reported | 85 | 145 | 0.5862 | Mapped to P5 (Self-reported familiarity or knowledge of AI) under operational definition: Proportion reporting familiarity, knowledge, understanding, or competence with AI concepts/tools. | Y |
| Safia Elwaleed Elhassan 2025 | P5 | Familiar with ChatGPT | Author-reported proportion; response scale not reported in the source article | Not clearly reported | Author-reported proportion; numerator is the endorsed response category | Neutral handling not explicitly reported; denominator retained as reported | 273 | 293 | 0.9317 | Mapped to P5 (Self-reported familiarity or knowledge of AI) under operational definition: Proportion reporting familiarity, knowledge, understanding, or competence with AI concepts/tools. | Y |
| Bayan Almehmadi 2025 | P5 | Aware of AI | Author-reported proportion; response scale not reported in the source article | Not clearly reported | Yes/endorsed category vs No/non-endorsed category | No neutral option reported; denominator retained as reported | 163 | 170 | 0.9588 | Mapped to P5 (Self-reported familiarity or knowledge of AI) under operational definition: Proportion reporting familiarity, knowledge, understanding, or competence with AI concepts/tools. | Y |
| Zahra Arab-Borzu 2025 | P5 | Understands what the term “artificial intelligence” means | response scale not reported in the source article | Not reported | Dichotomization method not reported in primary study; proportion extracted from reported percentage | Neutral handling not explicitly reported; denominator retained as reported | 168 | 279 | 0.6022 | Mapped to P5 (Self-reported familiarity or knowledge of AI) under operational definition: Proportion reporting familiarity, knowledge, understanding, or competence with AI concepts/tools. | Y |
| Florina Onetiu 2025 | P5 | High familiarity (Likert ≥4) with AI-based tools | Author-reported proportion; response scale not reported in the source article | Not clearly reported | Author-reported proportion; numerator is the endorsed response category | Neutral handling not explicitly reported; denominator retained as reported | 66 | 161 | 0.4099 | Mapped to P5 (Self-reported familiarity or knowledge of AI) under operational definition: Proportion reporting familiarity, knowledge, understanding, or competence with AI concepts/tools. | Y |
| Samah Alfahl 2025 | P5 | Familiarity with the basic concepts of AI | 5-point Likert agreement scale: strongly disagree to strongly agree | 5 | Author-reported proportion; numerator is the endorsed response category | Neutral handling not explicitly reported; denominator retained as reported | 102 | 189 | 0.5397 | Mapped to P5 (Self-reported familiarity or knowledge of AI) under operational definition: Proportion reporting familiarity, knowledge, understanding, or competence with AI concepts/tools. | Y |
| Ahmed Samir Abdelhafiz 2025 | P5 | Heard of ChatGPT prior to the study | Author-reported proportion; response scale not reported in the source article | Not clearly reported | Author-reported proportion; numerator is the endorsed response category | Neutral handling not explicitly reported; denominator retained as reported | 584 | 614 | 0.9511 | Mapped to P5 (Self-reported familiarity or knowledge of AI) under operational definition: Proportion reporting familiarity, knowledge, understanding, or competence with AI concepts/tools. | Y |
| Mingchan Liu 2026 | P5 | Familiar with the fundamental concepts of AI, such as machine learning and deep learning (agree + strongly agree) | Likert agreement scale; point count not reported in the source article | Not clearly reported | Agree + Strongly agree vs all other item responses | Neutral responses, if available, were excluded from numerator and retained in denominator | 373 | 788 | 0.4734 | Mapped to P5 (Self-reported familiarity or knowledge of AI) under operational definition: Proportion reporting familiarity, knowledge, understanding, or competence with AI concepts/tools. | Y |
| Shuo Duan 2025 | P5 | Understanding of AI applications specific to medical education | Author-reported proportion; response scale not reported in the source article | Not clearly reported | Author-reported proportion; numerator is the endorsed response category | Neutral handling not explicitly reported; denominator retained as reported | 241 | 553 | 0.4358 | Mapped to P5 (Self-reported familiarity or knowledge of AI) under operational definition: Proportion reporting familiarity, knowledge, understanding, or competence with AI concepts/tools. | Y |
| Hamdah Hanifa 2025 | P5 | Awareness of AI in the medical field | 5-point Likert agreement scale: strongly disagree to strongly agree | 5 | Dichotomization method not reported in primary study; proportion extracted from reported percentage | Neutral handling not explicitly reported; denominator retained as reported | 439 | 564 | 0.7784 | Mapped to P5 (Self-reported familiarity or knowledge of AI) under operational definition: Proportion reporting familiarity, knowledge, understanding, or competence with AI concepts/tools. | Y |
| Poorna Fernando 2025 | P5 | Basic understanding of AI concepts | Author-reported proportion; response scale not reported in the source article | Not clearly reported | Author-reported proportion; numerator is the endorsed response category | Neutral handling not explicitly reported; denominator retained as reported | 213 | 304 | 0.7007 | Mapped to P5 (Self-reported familiarity or knowledge of AI) under operational definition: Proportion reporting familiarity, knowledge, understanding, or competence with AI concepts/tools. | Y |
| Aleksandra Ignjatović 2025 | P5 | Have you heard about the artificial intelligence type ChatGPT' | Author-reported proportion; response scale not reported in the source article | Not clearly reported | Author-reported proportion; numerator is the endorsed response category | Neutral handling not explicitly reported; denominator retained as reported | 962 | 1212 | 0.7937 | Mapped to P5 (Self-reported familiarity or knowledge of AI) under operational definition: Proportion reporting familiarity, knowledge, understanding, or competence with AI concepts/tools. | Y |
| Emre Sanri 2025 | P5 | Self-reported moderate/high AI knowledge | response scale not reported in the source article | Not reported | Dichotomization method not reported in primary study; proportion extracted from reported percentage | Neutral handling not explicitly reported; denominator retained as reported | 92 | 120 | 0.7667 | Mapped to P5 (Self-reported familiarity or knowledge of AI) under operational definition: Proportion reporting familiarity, knowledge, understanding, or competence with AI concepts/tools. | Y |
| M Hassaan Shah 2025 | P5 | Very/somewhat familiar with AI technologies | Author-reported proportion; response scale not reported in the source article | Not clearly reported | Yes/endorsed category vs No/non-endorsed category | No neutral option reported; denominator retained as reported | 162 | 200 | 0.8100 | Mapped to P5 (Self-reported familiarity or knowledge of AI) under operational definition: Proportion reporting familiarity, knowledge, understanding, or competence with AI concepts/tools. | Y |
| Madhulatha Gopidesi 2025 | P5 | Good knowledge about AI tools being used for studying medicine | Author-reported proportion; response scale not reported in the source article | Not clearly reported | Author-reported proportion; numerator is the endorsed response category | Neutral handling not explicitly reported; denominator retained as reported | 129 | 210 | 0.6143 | Mapped to P5 (Self-reported familiarity or knowledge of AI) under operational definition: Proportion reporting familiarity, knowledge, understanding, or competence with AI concepts/tools. | Y |
| Faiza Ejas 2025 | P5 | Awareness of LLMs before the survey | Author-reported proportion; response scale not reported in the source article | Not clearly reported | Author-reported proportion; numerator is the endorsed response category | Neutral handling not explicitly reported; denominator retained as reported | 898 | 1180 | 0.7610 | Mapped to P5 (Self-reported familiarity or knowledge of AI) under operational definition: Proportion reporting familiarity, knowledge, understanding, or competence with AI concepts/tools. | Y |
| Seyyedeh Fatemeh Mousavi Baigi 2025 | P5 | Defined AI correctly or partially correctly in one sentence | Author-reported proportion; response scale not reported in the source article | Not clearly reported | Author-reported proportion; numerator is the endorsed response category | Neutral handling not explicitly reported; denominator retained as reported | 52 | 95 | 0.5474 | Mapped to P5 (Self-reported familiarity or knowledge of AI) under operational definition: Proportion reporting familiarity, knowledge, understanding, or competence with AI concepts/tools. | Y |
| Muhammad Farooq and Usmani (2025) | P5 | Good knowledge about AI | Author-reported proportion; response scale not reported in the source article | Not clearly reported | Author-reported proportion; numerator is the endorsed response category | Neutral handling not explicitly reported; denominator retained as reported | 139 | 152 | 0.9145 | Mapped to P5 (Self-reported familiarity or knowledge of AI) under operational definition: Proportion reporting familiarity, knowledge, understanding, or competence with AI concepts/tools. | N |
| Khalid S. Alharkan 2025 | P5 | Reported familiarity with AI | Likert agreement scale; point count not reported in the source article | Not clearly reported | Author-reported proportion; numerator is the endorsed response category | Neutral handling not explicitly reported; denominator retained as reported | 320 | 525 | 0.6095 | Mapped to P5 (Self-reported familiarity or knowledge of AI) under operational definition: Proportion reporting familiarity, knowledge, understanding, or competence with AI concepts/tools. | Y |
| Ahmed Hafez Allam 2024 | P5 | High/moderate AI knowledge level | Author-reported proportion; response scale not reported in the source article | Not clearly reported | Author-reported proportion; numerator is the endorsed response category | Neutral response category reported; not counted in numerator and retained in denominator unless source denominator was item-specific | 578 | 4492 | 0.1287 | Mapped to P5 (Self-reported familiarity or knowledge of AI) under operational definition: Proportion reporting familiarity, knowledge, understanding, or competence with AI concepts/tools. | Y |
| Wenjuan Tao 2024 | P5 | Fairly/very familiar with what an AI chatbot is | 5-point Likert agreement scale: strongly disagree to strongly agree | 5 | Author-reported proportion; numerator is the endorsed response category | Neutral handling not explicitly reported; denominator retained as reported | 202 | 693 | 0.2915 | Mapped to P5 (Self-reported familiarity or knowledge of AI) under operational definition: Proportion reporting familiarity, knowledge, understanding, or competence with AI concepts/tools. | Y |
| Conner Ganjavi 2024 | P5 | Had heard of ChatGPT | Author-reported proportion; response scale not reported in the source article | Not clearly reported | Author-reported proportion; numerator is the endorsed response category | Neutral handling not explicitly reported; denominator retained as reported | 398 | 415 | 0.9590 | Mapped to P5 (Self-reported familiarity or knowledge of AI) under operational definition: Proportion reporting familiarity, knowledge, understanding, or competence with AI concepts/tools. | Y |
| Fahad Abdullah Saeed Alwadani 2024 | P5 | I have an understanding of the basic computational principles of AI (agree + strongly agree) | 5-point Likert agreement scale: strongly disagree to strongly agree | 5 | Agree + Strongly agree vs all other item responses | Neutral responses, if available, were excluded from numerator and retained in denominator | 115 | 303 | 0.3795 | Mapped to P5 (Self-reported familiarity or knowledge of AI) under operational definition: Proportion reporting familiarity, knowledge, understanding, or competence with AI concepts/tools. | Y |
| Adnan Abu Hammour 2024 | P5 | Claimed to have knowledge of AI but not in clinical settings | 5-point Likert agreement scale: strongly disagree to strongly agree | 5 | Author-reported proportion; numerator is the endorsed response category | Neutral handling not explicitly reported; denominator retained as reported | 136 | 221 | 0.6154 | Mapped to P5 (Self-reported familiarity or knowledge of AI) under operational definition: Proportion reporting familiarity, knowledge, understanding, or competence with AI concepts/tools. | Y |
| Zainudheen Faroog 2024 | P5 | Heard of the use of AI in ophthalmology | Author-reported proportion; response scale not reported in the source article | Not clearly reported | Author-reported proportion; numerator is the endorsed response category | Neutral handling not explicitly reported; denominator retained as reported | 294 | 345 | 0.8522 | Mapped to P5 (Self-reported familiarity or knowledge of AI) under operational definition: Proportion reporting familiarity, knowledge, understanding, or competence with AI concepts/tools. | Y |
| Mohammed Hammad Jaber Amin 2024 | P5 | Do you know what artificial intelligence is' | Author-reported proportion; response scale not reported in the source article | Not clearly reported | Author-reported proportion; numerator is the endorsed response category | Neutral handling not explicitly reported; denominator retained as reported | 642 | 762 | 0.8425 | Mapped to P5 (Self-reported familiarity or knowledge of AI) under operational definition: Proportion reporting familiarity, knowledge, understanding, or competence with AI concepts/tools. | Y |
| Salita Angkurawaranon 2024 | P5 | Understanding of the basic computational principles of artificial intelligence (agree + strongly agree) | 5-point Likert agreement scale: strongly disagree to strongly agree | 5 | Agree + Strongly agree vs all other item responses | Neutral responses, if available, were excluded from numerator and retained in denominator | 111 | 352 | 0.3153 | Mapped to P5 (Self-reported familiarity or knowledge of AI) under operational definition: Proportion reporting familiarity, knowledge, understanding, or competence with AI concepts/tools. | Y |
| Omar A. Daher 2024 | P5 | I can define the basic concepts and terminology of AI (agree + strongly agree) | 5-point Likert agreement scale: strongly disagree to strongly agree | 5 | Agree + Strongly agree vs all other item responses | Neutral responses, if available, were excluded from numerator and retained in denominator | 96 | 365 | 0.2630 | Mapped to P5 (Self-reported familiarity or knowledge of AI) under operational definition: Proportion reporting familiarity, knowledge, understanding, or competence with AI concepts/tools. | Y |
| Anamaria Malešević 2024 | P5 | Familiar with the concept of AI | Author-reported proportion; response scale not reported in the source article | Not clearly reported | Author-reported proportion; numerator is the endorsed response category | Neutral handling not explicitly reported; denominator retained as reported | 650 | 1701 | 0.3821 | Mapped to P5 (Self-reported familiarity or knowledge of AI) under operational definition: Proportion reporting familiarity, knowledge, understanding, or competence with AI concepts/tools. | Y |
| Mohammad Hamad 2024 | P5 | Awareness of AI usage in the medical field | Author-reported proportion; response scale not reported in the source article | Not clearly reported | Yes/endorsed category vs No/non-endorsed category | No neutral option reported; denominator retained as reported | 631 | 858 | 0.7354 | Mapped to P5 (Self-reported familiarity or knowledge of AI) under operational definition: Proportion reporting familiarity, knowledge, understanding, or competence with AI concepts/tools. | Y |
| Dalia M. Alammari 2024 | P5 | Have a background/familiarity with AI | Author-reported proportion; response scale not reported in the source article | Not clearly reported | Author-reported proportion; numerator is the endorsed response category | Neutral handling not explicitly reported; denominator retained as reported | 156 | 207 | 0.7536 | Mapped to P5 (Self-reported familiarity or knowledge of AI) under operational definition: Proportion reporting familiarity, knowledge, understanding, or competence with AI concepts/tools. | Y |
| Gamze Özbek Güven 2024 | P5 | Heard about artificial intelligence before | Author-reported proportion; response scale not reported in the source article | Not clearly reported | Author-reported proportion; numerator is the endorsed response category | Neutral handling not explicitly reported; denominator retained as reported | 530 | 542 | 0.9779 | Mapped to P5 (Self-reported familiarity or knowledge of AI) under operational definition: Proportion reporting familiarity, knowledge, understanding, or competence with AI concepts/tools. | Y |
| Alan Y. Xu 2024 | P5 | Heard of LLM-based chatbots such as ChatGPT/Bard/Bing/Claude | response scale not reported in the source article | Not reported | Dichotomization method not reported in primary study; proportion extracted from reported percentage | Neutral handling not explicitly reported; denominator retained as reported | 102 | 102 | 1.0000 | Mapped to P5 (Self-reported familiarity or knowledge of AI) under operational definition: Proportion reporting familiarity, knowledge, understanding, or competence with AI concepts/tools. | Y |
| Khaldoon Al-Roomi 2024 | P5 | What is your current level of familiarity with artificial intelligence (AI)' (very/somewhat familiar) | response scale not reported in the source article | Not reported | Dichotomization method not reported in primary study; proportion extracted from reported percentage | Neutral handling not explicitly reported; denominator retained as reported | 133 | 152 | 0.8750 | Mapped to P5 (Self-reported familiarity or knowledge of AI) under operational definition: Proportion reporting familiarity, knowledge, understanding, or competence with AI concepts/tools. | Y |
| Alvin Yong Zong Tung 2023 | P5 | I understand the basic computational principles of AI | 5-point Likert agreement scale: strongly disagree to strongly agree | 5 | Author-reported proportion; numerator is the endorsed response category | Neutral response category reported; not counted in numerator and retained in denominator unless source denominator was item-specific | 93 | 301 | 0.3090 | Mapped to P5 (Self-reported familiarity or knowledge of AI) under operational definition: Proportion reporting familiarity, knowledge, understanding, or competence with AI concepts/tools. | Y |
| Jonathon Stewart 2023 | P5 | I have a basic understanding of what artificial intelligence is | Likert agreement scale; point count not reported in the source article | Not clearly reported | Agree + Somewhat agree + Strongly agree vs all other item responses | Neutral responses, if available, were excluded from numerator and retained in denominator | 114 | 134 | 0.8507 | Mapped to P5 (Self-reported familiarity or knowledge of AI) under operational definition: Proportion reporting familiarity, knowledge, understanding, or competence with AI concepts/tools. | Y |
| Ali Jasem Buabbas 2023 | P5 | Understood the basic principles of AI | Author-reported proportion; response scale not reported in the source article | Not clearly reported | Author-reported proportion; numerator is the endorsed response category | Neutral handling not explicitly reported; denominator retained as reported | 213 | 352 | 0.6051 | Mapped to P5 (Self-reported familiarity or knowledge of AI) under operational definition: Proportion reporting familiarity, knowledge, understanding, or competence with AI concepts/tools. | Y |
| Andrés Barreiro-Ares 2023 | P5 | Claimed that they knew what AI is | Author-reported proportion; response scale not reported in the source article | Not clearly reported | Author-reported proportion; numerator is the endorsed response category | Neutral handling not explicitly reported; denominator retained as reported | 223 | 281 | 0.7936 | Mapped to P5 (Self-reported familiarity or knowledge of AI) under operational definition: Proportion reporting familiarity, knowledge, understanding, or competence with AI concepts/tools. | Y |
| Zaboor Ahmed 2022 | P5 | Basic knowledge of AI | Author-reported proportion; response scale not reported in the source article | Not clearly reported | Author-reported proportion; numerator is the endorsed response category | Neutral handling not explicitly reported; denominator retained as reported | 170 | 247 | 0.6883 | Mapped to P5 (Self-reported familiarity or knowledge of AI) under operational definition: Proportion reporting familiarity, knowledge, understanding, or competence with AI concepts/tools. | Y |
| G. Caparrós Galán 2022 | P5 | Considered that they clearly understood what artificial intelligence is | Author-reported proportion; response scale not reported in the source article | Not clearly reported | Yes/endorsed category vs No/non-endorsed category | No neutral option reported; denominator retained as reported | 177 | 341 | 0.5191 | Mapped to P5 (Self-reported familiarity or knowledge of AI) under operational definition: Proportion reporting familiarity, knowledge, understanding, or competence with AI concepts/tools. | Y |
| Aidan Pucchio 2022 | P5 | I could provide examples of AI/ML/neural network/deep learning applications in medicine | Likert agreement scale; point count not reported in the source article | Not clearly reported | Author-reported proportion; numerator is the endorsed response category | Neutral response category reported; not counted in numerator and retained in denominator unless source denominator was item-specific | 216 | 463 | 0.4665 | Mapped to P5 (Self-reported familiarity or knowledge of AI) under operational definition: Proportion reporting familiarity, knowledge, understanding, or competence with AI concepts/tools. | Y |
| Stuart McLennan 2022 | P5 | Feeling well informed about AI in medicine | Author-reported proportion; response scale not reported in the source article | Not clearly reported | Author-reported proportion; numerator is the endorsed response category | Neutral handling not explicitly reported; denominator retained as reported | 300 | 844 | 0.3555 | Mapped to P5 (Self-reported familiarity or knowledge of AI) under operational definition: Proportion reporting familiarity, knowledge, understanding, or competence with AI concepts/tools. | Y |
| Charlotte Blease 2022 | P5 | Have you heard of machine learning' | Author-reported proportion; response scale not reported in the source article | Not clearly reported | Yes/endorsed category vs No/non-endorsed category | No neutral option reported; denominator retained as reported | 137 | 242 | 0.5661 | Mapped to P5 (Self-reported familiarity or knowledge of AI) under operational definition: Proportion reporting familiarity, knowledge, understanding, or competence with AI concepts/tools. | Y |
| Elena A Wood 2021 | P5 | Aware of AI topics in medicine | Author-reported proportion; response scale not reported in the source article | Not clearly reported | Author-reported proportion; numerator is the endorsed response category | Neutral handling not explicitly reported; denominator retained as reported | 35 | 117 | 0.2991 | Mapped to P5 (Self-reported familiarity or knowledge of AI) under operational definition: Proportion reporting familiarity, knowledge, understanding, or competence with AI concepts/tools. | Y |
| Cherry Sit 2020 | P5 | Have an understanding of the basic computational principles that underpin AI | 5-point Likert agreement scale: strongly disagree to strongly agree | 5 | Agree + Strongly agree vs all other item responses | Neutral responses, if available, were excluded from numerator and retained in denominator | 214 | 484 | 0.4421 | Mapped to P5 (Self-reported familiarity or knowledge of AI) under operational definition: Proportion reporting familiarity, knowledge, understanding, or competence with AI concepts/tools. | Y |
| D. Pinto dos Santos 2019 | P5 | Do you personally have a basic understanding of the technologies used in these topics' | Author-reported proportion; response scale not reported in the source article | Not clearly reported | Yes/endorsed category vs No/non-endorsed category | No neutral option reported; denominator retained as reported | 81 | 263 | 0.3080 | Mapped to P5 (Self-reported familiarity or knowledge of AI) under operational definition: Proportion reporting familiarity, knowledge, understanding, or competence with AI concepts/tools. | Y |
| Xi Cao 2026 | P6 | Prior experience with generative AI | Author-reported categorical response/proportion; exact response options not fully reported | Not fully reported | Author-reported proportion; numerator is the endorsed response category | Neutral handling not explicitly reported; denominator retained as reported | 1056 | 1062 | 0.9944 | Mapped to P6 (Prior use of or exposure to AI tools) under operational definition: Proportion reporting having personally used any AI tool (ChatGPT, clinical DSS, imaging AI, etc.) or having had formal/informal exposure to AI. | Y |
| Ali Veysel Kara 2026 | P6 | Use of AI for education or clinical simulation | Author-reported categorical response/proportion; exact response options not fully reported | Not fully reported | Author-reported proportion; numerator is the endorsed response category | Neutral handling not explicitly reported; denominator retained as reported | 934 | 1346 | 0.6939 | Mapped to P6 (Prior use of or exposure to AI tools) under operational definition: Proportion reporting having personally used any AI tool (ChatGPT, clinical DSS, imaging AI, etc.) or having had formal/informal exposure to AI. | Y |
| Rixiang Xu 2026 | P6 | Use of AI for learning tasks and clinical case analysis | 7-point Likert agreement scale | 7 | Author-reported proportion; numerator is the endorsed response category | Neutral handling not explicitly reported; denominator retained as reported | 654 | 661 | 0.9894 | Mapped to P6 (Prior use of or exposure to AI tools) under operational definition: Proportion reporting having personally used any AI tool (ChatGPT, clinical DSS, imaging AI, etc.) or having had formal/informal exposure to AI. | Y |
| Alan Yuesheng Xu 2026 | P6 | Prior use of ChatGPT | Author-reported categorical response/proportion; exact response options not fully reported | Not fully reported | Author-reported proportion; numerator is the endorsed response category | Neutral handling not explicitly reported; denominator retained as reported | 157 | 177 | 0.887 | Mapped to P6 (Prior use of or exposure to AI tools) under operational definition: Proportion reporting having personally used any AI tool (ChatGPT, clinical DSS, imaging AI, etc.) or having had formal/informal exposure to AI. | Y |
| Na Hu 2025 | P6 | Use of ChatGPT for information searching or assignments | Author-reported proportion; response scale not reported in the source article | Not clearly reported | Author-reported proportion; numerator is the endorsed response category | Neutral handling not explicitly reported; denominator retained as reported | 713 | 1133 | 0.6293 | Mapped to P6 (Prior use of or exposure to AI tools) under operational definition: Proportion reporting having personally used any AI tool (ChatGPT, clinical DSS, imaging AI, etc.) or having had formal/informal exposure to AI. | Y |
| Omar A. Ghanem 2025 | P6 | Prior use of generative AI for research | Author-reported categorical response/proportion; exact response options not fully reported | Not fully reported | Dichotomization method not reported in primary study; proportion extracted from reported percentage | Neutral handling not explicitly reported; denominator retained as reported | 318 | 423 | 0.7518 | Mapped to P6 (Prior use of or exposure to AI tools) under operational definition: Proportion reporting having personally used any AI tool (ChatGPT, clinical DSS, imaging AI, etc.) or having had formal/informal exposure to AI. | Y |
| Weam Mohamed Meargni Ahmed 2025 | P6 | Prior practical use of AI | Author-reported categorical response/proportion; exact response options not fully reported | Not fully reported | Author-reported proportion; numerator is the endorsed response category | Neutral handling not explicitly reported; denominator retained as reported | 605 | 1443 | 0.4193 | Mapped to P6 (Prior use of or exposure to AI tools) under operational definition: Proportion reporting having personally used any AI tool (ChatGPT, clinical DSS, imaging AI, etc.) or having had formal/informal exposure to AI. | Y |
| Bruna Giavina-Bianchi 2025 | P6 | Exposure to a melanoma-detection AI scenario | Author-reported proportion; response scale not reported in the source article | Not clearly reported | Author-reported proportion; numerator is the endorsed response category | Neutral handling not explicitly reported; denominator retained as reported | 61 | 145 | 0.4207 | Mapped to P6 (Prior use of or exposure to AI tools) under operational definition: Proportion reporting having personally used any AI tool (ChatGPT, clinical DSS, imaging AI, etc.) or having had formal/informal exposure to AI. | Y |
| Anna Fußhöller 2025 | P6 | Use of ChatGPT for medical education | Author-reported categorical response/proportion; exact response options not fully reported | Not fully reported | Author-reported proportion; numerator is the endorsed response category | Neutral handling not explicitly reported; denominator retained as reported | 64 | 84 | 0.7619 | Mapped to P6 (Prior use of or exposure to AI tools) under operational definition: Proportion reporting having personally used any AI tool (ChatGPT, clinical DSS, imaging AI, etc.) or having had formal/informal exposure to AI. | Y |
| Cecilia Tran 2025 | P6 | Active use of generative AI | Author-reported categorical response/proportion; exact response options not fully reported | Not fully reported | Author-reported proportion; numerator is the endorsed response category | Neutral handling not explicitly reported; denominator retained as reported | 131 | 166 | 0.7892 | Mapped to P6 (Prior use of or exposure to AI tools) under operational definition: Proportion reporting having personally used any AI tool (ChatGPT, clinical DSS, imaging AI, etc.) or having had formal/informal exposure to AI. | Y |
| Bayan Almehmadi 2025 | P6 | Have you used ChatGPT? | Author-reported categorical response/proportion; exact response options not fully reported | Not fully reported | Yes/endorsed category vs No/non-endorsed category | No neutral option reported; denominator retained as reported | 160 | 170 | 0.9412 | Mapped to P6 (Prior use of or exposure to AI tools) under operational definition: Proportion reporting having personally used any AI tool (ChatGPT, clinical DSS, imaging AI, etc.) or having had formal/informal exposure to AI. | Y |
| Samah Alfahl 2025 | P6 | Prior use of AI tools for studying or research | Author-reported categorical response/proportion; exact response options not fully reported | Not fully reported | Author-reported proportion; numerator is the endorsed response category | Neutral handling not explicitly reported; denominator retained as reported | 98 | 189 | 0.5185 | Mapped to P6 (Prior use of or exposure to AI tools) under operational definition: Proportion reporting having personally used any AI tool (ChatGPT, clinical DSS, imaging AI, etc.) or having had formal/informal exposure to AI. | Y |
| Min Zhang 2025 | P6 | Frequency of use of AI large language models | 5-point Likert agreement scale: strongly disagree to strongly agree | 5 | Dichotomization method not reported in primary study; proportion extracted from reported percentage | Neutral response category reported; not counted in numerator and retained in denominator unless source denominator was item-specific | 241 | 254 | 0.9488 | Mapped to P6 (Prior use of or exposure to AI tools) under operational definition: Proportion reporting having personally used any AI tool (ChatGPT, clinical DSS, imaging AI, etc.) or having had formal/informal exposure to AI. | Y |
| Abdul Sami 2025 | P6 | Prior use of AI tools in medical education | response scale not reported in the source article | Not reported | Dichotomization method not reported in primary study; proportion extracted from reported percentage | Neutral handling not explicitly reported; denominator retained as reported | 416 | 702 | 0.5926 | Mapped to P6 (Prior use of or exposure to AI tools) under operational definition: Proportion reporting having personally used any AI tool (ChatGPT, clinical DSS, imaging AI, etc.) or having had formal/informal exposure to AI. | N |
| Ahmed Samir Abdelhafiz 2025 | P6 | Prior use of ChatGPT | Author-reported categorical response/proportion; exact response options not fully reported | Not fully reported | Yes/endorsed category vs No/non-endorsed category | No neutral option reported; denominator retained as reported | 482 | 614 | 0.7850 | Mapped to P6 (Prior use of or exposure to AI tools) under operational definition: Proportion reporting having personally used any AI tool (ChatGPT, clinical DSS, imaging AI, etc.) or having had formal/informal exposure to AI. | Y |
| Mingchan Liu 2026 | P6 | Use of AI tools for information searching or learning | Author-reported categorical response/proportion; exact response options not fully reported | Not fully reported | Dichotomization method not reported in primary study; proportion extracted from reported percentage | Neutral handling not explicitly reported; denominator retained as reported | 720 | 788 | 0.9137 | Mapped to P6 (Prior use of or exposure to AI tools) under operational definition: Proportion reporting having personally used any AI tool (ChatGPT, clinical DSS, imaging AI, etc.) or having had formal/informal exposure to AI. | Y |
| Hamdah Hanifa 2025 | P6 | Prior experience with AI | Dichotomous Yes/No response | 2 | Yes/endorsed category vs No/non-endorsed category | No neutral option reported; denominator retained as reported | 157 | 564 | 0.2784 | Mapped to P6 (Prior use of or exposure to AI tools) under operational definition: Proportion reporting having personally used any AI tool (ChatGPT, clinical DSS, imaging AI, etc.) or having had formal/informal exposure to AI. | Y |
| Juan José Gualda-Gea 2025 | P6 | Prior use of chatbots for medical information | Author-reported categorical response/proportion; exact response options not fully reported | Not fully reported | Author-reported proportion; numerator is the endorsed response category | Neutral handling not explicitly reported; denominator retained as reported | 9 | 29 | 0.3103 | Mapped to P6 (Prior use of or exposure to AI tools) under operational definition: Proportion reporting having personally used any AI tool (ChatGPT, clinical DSS, imaging AI, etc.) or having had formal/informal exposure to AI. | Y |
| Aleksandra Ignjatović 2025 | P6 | Prior use of ChatGPT | Author-reported categorical response/proportion; exact response options not fully reported | Not fully reported | Author-reported proportion; numerator is the endorsed response category | Neutral handling not explicitly reported; denominator retained as reported | 511 | 1212 | 0.4216 | Mapped to P6 (Prior use of or exposure to AI tools) under operational definition: Proportion reporting having personally used any AI tool (ChatGPT, clinical DSS, imaging AI, etc.) or having had formal/informal exposure to AI. | Y |
| M Hassaan Shah 2025 | P6 | Use of AI tools for studying or research | Author-reported categorical response/proportion; exact response options not fully reported | Not fully reported | Yes/endorsed category vs No/non-endorsed category | No neutral option reported; denominator retained as reported | 118 | 200 | 0.5900 | Mapped to P6 (Prior use of or exposure to AI tools) under operational definition: Proportion reporting having personally used any AI tool (ChatGPT, clinical DSS, imaging AI, etc.) or having had formal/informal exposure to AI. | Y |
| Madhulatha Gopidesi 2025 | P6 | Use of at least one AI tool in medical studies | Author-reported categorical response/proportion; exact response options not fully reported | Not fully reported | Author-reported proportion; numerator is the endorsed response category | Neutral handling not explicitly reported; denominator retained as reported | 169 | 210 | 0.8048 | Mapped to P6 (Prior use of or exposure to AI tools) under operational definition: Proportion reporting having personally used any AI tool (ChatGPT, clinical DSS, imaging AI, etc.) or having had formal/informal exposure to AI. | Y |
| Faiza Ejas 2025 | P6 | Have you heard about LLMs? | Author-reported categorical response/proportion; exact response options not fully reported | Not fully reported | Yes/endorsed category vs No/non-endorsed category | No neutral option reported; denominator retained as reported | 646 | 898 | 0.7194 | Mapped to P6 (Prior use of or exposure to AI tools) under operational definition: Proportion reporting having personally used any AI tool (ChatGPT, clinical DSS, imaging AI, etc.) or having had formal/informal exposure to AI. | Y |
| Adhari AlZaabi 2025 | P6 | Use of AI-based information with professional knowledge | Author-reported proportion; response scale not reported in the source article | Not clearly reported | Author-reported proportion; numerator is the endorsed response category | Neutral handling not explicitly reported; denominator retained as reported | 67 | 84 | 0.7976 | Mapped to P6 (Prior use of or exposure to AI tools) under operational definition: Proportion reporting having personally used any AI tool (ChatGPT, clinical DSS, imaging AI, etc.) or having had formal/informal exposure to AI. | Y |
| Khalid S. Alharkan 2025 | P6 | Prior use or development of AI products | Author-reported categorical response/proportion; exact response options not fully reported | Not fully reported | Dichotomization method not reported in primary study; proportion extracted from reported percentage | Neutral handling not explicitly reported; denominator retained as reported | 320 | 525 | 0.6095 | Mapped to P6 (Prior use of or exposure to AI tools) under operational definition: Proportion reporting having personally used any AI tool (ChatGPT, clinical DSS, imaging AI, etc.) or having had formal/informal exposure to AI. | Y |
| Adrián E. González-Bravo 2025 | P6 | Prior use of AI tools in medical education | Author-reported categorical response/proportion; exact response options not fully reported | Not fully reported | Author-reported proportion; numerator is the endorsed response category | Neutral handling not explicitly reported; denominator retained as reported | 141 | 194 | 0.7268 | Mapped to P6 (Prior use of or exposure to AI tools) under operational definition: Proportion reporting having personally used any AI tool (ChatGPT, clinical DSS, imaging AI, etc.) or having had formal/informal exposure to AI. | Y |
| Wenjuan Tao 2024 | P6 | Use of AI chatbots for studying | Author-reported categorical response/proportion; exact response options not fully reported | Not fully reported | Author-reported proportion; numerator is the endorsed response category | Neutral handling not explicitly reported; denominator retained as reported | 199 | 693 | 0.2872 | Mapped to P6 (Prior use of or exposure to AI tools) under operational definition: Proportion reporting having personally used any AI tool (ChatGPT, clinical DSS, imaging AI, etc.) or having had formal/informal exposure to AI. | Y |
| Conner Ganjavi 2024 | P6 | Prior use of an AI tool | Author-reported categorical response/proportion; exact response options not fully reported | Not fully reported | Author-reported proportion; numerator is the endorsed response category | Neutral handling not explicitly reported; denominator retained as reported | 216 | 415 | 0.5205 | Mapped to P6 (Prior use of or exposure to AI tools) under operational definition: Proportion reporting having personally used any AI tool (ChatGPT, clinical DSS, imaging AI, etc.) or having had formal/informal exposure to AI. | Y |
| Fahad Abdullah Saeed Alwadani 2024 | P6 | Prior training on artificial intelligence | Author-reported proportion; response scale not reported in the source article | Not clearly reported | Author-reported proportion; numerator is the endorsed response category | Neutral handling not explicitly reported; denominator retained as reported | 42 | 303 | 0.1386 | Mapped to P6 (Prior use of or exposure to AI tools) under operational definition: Proportion reporting having personally used any AI tool (ChatGPT, clinical DSS, imaging AI, etc.) or having had formal/informal exposure to AI. | Y |
| Adnan Abu Hammour 2024 | P6 | Prior exposure to ChatGPT during medical training | response scale not reported in the source article | Not reported | Dichotomization method not reported in primary study; proportion extracted from reported percentage | Neutral handling not explicitly reported; denominator retained as reported | 177 | 244 | 0.7254 | Mapped to P6 (Prior use of or exposure to AI tools) under operational definition: Proportion reporting having personally used any AI tool (ChatGPT, clinical DSS, imaging AI, etc.) or having had formal/informal exposure to AI. | Y |
| Mohammed Hammad Jaber Amin 2024 | P6 | Prior practical application of AI | response scale not reported in the source article | Not reported | Dichotomization method not reported in primary study; proportion extracted from reported percentage | Neutral handling not explicitly reported; denominator retained as reported | 267 | 762 | 0.3504 | Mapped to P6 (Prior use of or exposure to AI tools) under operational definition: Proportion reporting having personally used any AI tool (ChatGPT, clinical DSS, imaging AI, etc.) or having had formal/informal exposure to AI. | Y |
| Lukas Weidener 2024 | P6 | Prior use of AI in medicine | response scale not reported in the source article | Not reported | Dichotomization method not reported in primary study; proportion extracted from reported percentage | Neutral handling not explicitly reported; denominator retained as reported | 189 | 487 | 0.3881 | Mapped to P6 (Prior use of or exposure to AI tools) under operational definition: Proportion reporting having personally used any AI tool (ChatGPT, clinical DSS, imaging AI, etc.) or having had formal/informal exposure to AI. | Y |
| Abeer Al Shahrani 2024 | P6 | Use of AI applications such as ChatGPT | Author-reported categorical response/proportion; exact response options not fully reported | Not fully reported | Yes/endorsed category vs No/non-endorsed category | No neutral option reported; denominator retained as reported | 533 | 572 | 0.9318 | Mapped to P6 (Prior use of or exposure to AI tools) under operational definition: Proportion reporting having personally used any AI tool (ChatGPT, clinical DSS, imaging AI, etc.) or having had formal/informal exposure to AI. | Y |
| Rohankumar Gandhi 2024 | P6 | Prior use of AI tools | Author-reported categorical response/proportion; exact response options not fully reported | Not fully reported | Dichotomization method not reported in primary study; proportion extracted from reported percentage | Neutral handling not explicitly reported; denominator retained as reported | 278 | 605 | 0.4595 | Mapped to P6 (Prior use of or exposure to AI tools) under operational definition: Proportion reporting having personally used any AI tool (ChatGPT, clinical DSS, imaging AI, etc.) or having had formal/informal exposure to AI. | Y |
| Alan Y. Xu 2024 | P6 | Prior use of large language models for medical tasks | Author-reported categorical response/proportion; exact response options not fully reported | Not fully reported | Author-reported proportion; numerator is the endorsed response category | Neutral handling not explicitly reported; denominator retained as reported | 70 | 102 | 0.6863 | Mapped to P6 (Prior use of or exposure to AI tools) under operational definition: Proportion reporting having personally used any AI tool (ChatGPT, clinical DSS, imaging AI, etc.) or having had formal/informal exposure to AI. | Y |
| Khaldoon Al-Roomi 2024 | P6 | Use of AI applications | Author-reported proportion; response scale not reported in the source article | Not clearly reported | Author-reported proportion; numerator is the endorsed response category | Neutral handling not explicitly reported; denominator retained as reported | 67 | 152 | 0.4408 | Mapped to P6 (Prior use of or exposure to AI tools) under operational definition: Proportion reporting having personally used any AI tool (ChatGPT, clinical DSS, imaging AI, etc.) or having had formal/informal exposure to AI. | Y |
| Zinah A. Al Hadithy 2023 | P6 | Prior exposure to artificial intelligence | Author-reported categorical response/proportion; exact response options not fully reported | Not fully reported | Dichotomization method not reported in primary study; proportion extracted from reported percentage | Neutral handling not explicitly reported; denominator retained as reported | 54 | 221 | 0.2443 | Mapped to P6 (Prior use of or exposure to AI tools) under operational definition: Proportion reporting having personally used any AI tool (ChatGPT, clinical DSS, imaging AI, etc.) or having had formal/informal exposure to AI. | Y |
| Zaboor Ahmed 2022 | P6 | Prior use of artificial intelligence tools | Author-reported proportion; response scale not reported in the source article | Not clearly reported | Author-reported proportion; numerator is the endorsed response category | Neutral handling not explicitly reported; denominator retained as reported | 20 | 247 | 0.0810 | Mapped to P6 (Prior use of or exposure to AI tools) under operational definition: Proportion reporting having personally used any AI tool (ChatGPT, clinical DSS, imaging AI, etc.) or having had formal/informal exposure to AI. | Y |
| Xi Cao 2026 | P7 | Willingness to use generative AI in the future | Author-reported categorical response/proportion; exact response options not fully reported | Not fully reported | Author-reported proportion; numerator is the endorsed response category | Neutral handling not explicitly reported; denominator retained as reported | 762 | 1062 | 0.7175 | Mapped to P7 (Willingness to learn or adopt AI) under operational definition: Proportion expressing willingness, readiness, or intention to learn about AI, use AI tools, or integrate AI into their future practice. | Y |
| Alan Yuesheng Xu 2026 | P7 | Intention to continue using ChatGPT or similar AI chatbots | Author-reported proportion; response scale not reported in the source article | Not clearly reported | Author-reported proportion; numerator is the endorsed response category | Neutral handling not explicitly reported; denominator retained as reported | 135 | 157 | 0.8599 | Mapped to P7 (Willingness to learn or adopt AI) under operational definition: Proportion expressing willingness, readiness, or intention to learn about AI, use AI tools, or integrate AI into their future practice. | Y |
| Yacoub A. Yousef 2026 | P7 | Cautious adoption of AI-assisted ophthalmology interventions | response scale not reported in the source article | Not reported | Dichotomization method not reported in primary study; proportion extracted from reported percentage | Neutral handling not explicitly reported; denominator retained as reported | 277 | 353 | 0.7847 | Mapped to P7 (Willingness to learn or adopt AI) under operational definition: Proportion expressing willingness, readiness, or intention to learn about AI, use AI tools, or integrate AI into their future practice. | Y |
| Na Hu 2025 | P7 | Willingness to use ChatGPT for academic research | Author-reported proportion; response scale not reported in the source article | Not clearly reported | Author-reported proportion; numerator is the endorsed response category | Neutral response category reported; not counted in numerator and retained in denominator unless source denominator was item-specific | 433 | 713 | 0.6073 | Mapped to P7 (Willingness to learn or adopt AI) under operational definition: Proportion expressing willingness, readiness, or intention to learn about AI, use AI tools, or integrate AI into their future practice. | Y |
| Bruna Giavina-Bianchi 2025 | P7 | Intention to use reliable and available AI solutions | Author-reported categorical response/proportion; exact response options not fully reported | Not fully reported | Author-reported proportion; numerator is the endorsed response category | Neutral handling not explicitly reported; denominator retained as reported | 107 | 145 | 0.7379 | Mapped to P7 (Willingness to learn or adopt AI) under operational definition: Proportion expressing willingness, readiness, or intention to learn about AI, use AI tools, or integrate AI into their future practice. | Y |
| Safia Elwaleed Elhassan 2025 | P7 | Plan to incorporate ChatGPT or similar AI apps into learning | Author-reported proportion; response scale not reported in the source article | Not clearly reported | Author-reported proportion; numerator is the endorsed response category | Neutral response category reported; not counted in numerator and retained in denominator unless source denominator was item-specific | 149 | 293 | 0.5085 | Mapped to P7 (Willingness to learn or adopt AI) under operational definition: Proportion expressing willingness, readiness, or intention to learn about AI, use AI tools, or integrate AI into their future practice. | Y |
| Cecilia Tran 2025 | P7 | Willingness to learn and integrate generative AI into future practice | Author-reported categorical response/proportion; exact response options not fully reported | Not fully reported | Author-reported proportion; numerator is the endorsed response category | Neutral handling not explicitly reported; denominator retained as reported | 154 | 165 | 0.9333 | Mapped to P7 (Willingness to learn or adopt AI) under operational definition: Proportion expressing willingness, readiness, or intention to learn about AI, use AI tools, or integrate AI into their future practice. | Y |
| Zahra Arab-Borzu 2025 | P7 | Readiness to adopt AI in healthcare practice | response scale not reported in the source article | Not reported | Dichotomization method not reported in primary study; proportion extracted from reported percentage | Neutral handling not explicitly reported; denominator retained as reported | 229 | 280 | 0.8179 | Mapped to P7 (Willingness to learn or adopt AI) under operational definition: Proportion expressing willingness, readiness, or intention to learn about AI, use AI tools, or integrate AI into their future practice. | Y |
| Min Zhang 2025 | P7 | Willingness to use AI large language models in medical education | Author-reported categorical response/proportion; exact response options not fully reported | Not fully reported | Author-reported proportion; numerator is the endorsed response category | Neutral handling not explicitly reported; denominator retained as reported | 194 | 249 | 0.7791 | Mapped to P7 (Willingness to learn or adopt AI) under operational definition: Proportion expressing willingness, readiness, or intention to learn about AI, use AI tools, or integrate AI into their future practice. | Y |
| Ahmed Samir Abdelhafiz 2025 | P7 | Willingness to use ChatGPT for studies | 5-point Likert agreement scale: strongly disagree to strongly agree | 5 | Author-reported proportion; numerator is the endorsed response category | Neutral response category reported; not counted in numerator and retained in denominator unless source denominator was item-specific | 441 | 614 | 0.7182 | Mapped to P7 (Willingness to learn or adopt AI) under operational definition: Proportion expressing willingness, readiness, or intention to learn about AI, use AI tools, or integrate AI into their future practice. | Y |
| Shuo Duan 2025 | P7 | Willingness to adopt AI in medical education | response scale not reported in the source article | Not reported | Dichotomization method not reported in primary study; proportion extracted from reported percentage | Neutral handling not explicitly reported; denominator retained as reported | 253 | 553 | 0.4575 | Mapped to P7 (Willingness to learn or adopt AI) under operational definition: Proportion expressing willingness, readiness, or intention to learn about AI, use AI tools, or integrate AI into their future practice. | Y |
| Juan José Gualda-Gea 2025 | P7 | Interest in practical applications of AI in medicine | response scale not reported in the source article | Not reported | Dichotomization method not reported in primary study; proportion extracted from reported percentage | Neutral handling not explicitly reported; denominator retained as reported | 24 | 29 | 0.8276 | Mapped to P7 (Willingness to learn or adopt AI) under operational definition: Proportion expressing willingness, readiness, or intention to learn about AI, use AI tools, or integrate AI into their future practice. | Y |
| M Hassaan Shah 2025 | P7 | Interest in AI workshops or training programs | Author-reported proportion; response scale not reported in the source article | Not clearly reported | Yes/endorsed category vs No/non-endorsed category | No neutral option reported; denominator retained as reported | 135 | 200 | 0.6750 | Mapped to P7 (Willingness to learn or adopt AI) under operational definition: Proportion expressing willingness, readiness, or intention to learn about AI, use AI tools, or integrate AI into their future practice. | Y |
| Abeer F. Almarzouki 2025 | P7 | Readiness to adopt AI in clinical care | response scale not reported in the source article | Not reported | Dichotomization method not reported in primary study; proportion extracted from reported percentage | Neutral handling not explicitly reported; denominator retained as reported | 229 | 354 | 0.6469 | Mapped to P7 (Willingness to learn or adopt AI) under operational definition: Proportion expressing willingness, readiness, or intention to learn about AI, use AI tools, or integrate AI into their future practice. | Y |
| Kamel Jebreen 2024 | P7 | Interest in studying AI applications in medicine | Author-reported proportion; response scale not reported in the source article | Not clearly reported | Author-reported proportion; numerator is the endorsed response category | Neutral handling not explicitly reported; denominator retained as reported | 228 | 349 | 0.6533 | Mapped to P7 (Willingness to learn or adopt AI) under operational definition: Proportion expressing willingness, readiness, or intention to learn about AI, use AI tools, or integrate AI into their future practice. | Y |
| Lukas Weidener 2024 | P7 | Willingness to learn about AI in medicine | 5-point Likert agreement scale: strongly disagree to strongly agree | 5 | Dichotomization method not reported in primary study; proportion extracted from reported percentage | Neutral handling not explicitly reported; denominator retained as reported | 404 | 487 | 0.8296 | Mapped to P7 (Willingness to learn or adopt AI) under operational definition: Proportion expressing willingness, readiness, or intention to learn about AI, use AI tools, or integrate AI into their future practice. | Y |
| Anamaria Malešević 2024 | P7 | Willingness to adopt AI in healthcare | Author-reported categorical response/proportion; exact response options not fully reported | Not fully reported | Author-reported proportion; numerator is the endorsed response category | Neutral handling not explicitly reported; denominator retained as reported | 762 | 1701 | 0.4480 | Mapped to P7 (Willingness to learn or adopt AI) under operational definition: Proportion expressing willingness, readiness, or intention to learn about AI, use AI tools, or integrate AI into their future practice. | Y |
| Rohankumar Gandhi 2024 | P7 | Concern about the learning curve for adopting AI | response scale not reported in the source article | Not reported | Dichotomization method not reported in primary study; proportion extracted from reported percentage | Neutral handling not explicitly reported; denominator retained as reported | 444 | 605 | 0.7339 | Mapped to P7 (Willingness to learn or adopt AI) under operational definition: Proportion expressing willingness, readiness, or intention to learn about AI, use AI tools, or integrate AI into their future practice. | Y |
| Alan Y. Xu 2024 | P7 | Intention to continue using large language models in future practice | Author-reported categorical response/proportion; exact response options not fully reported | Not fully reported | Author-reported proportion; numerator is the endorsed response category | Neutral handling not explicitly reported; denominator retained as reported | 55 | 70 | 0.7857 | Mapped to P7 (Willingness to learn or adopt AI) under operational definition: Proportion expressing willingness, readiness, or intention to learn about AI, use AI tools, or integrate AI into their future practice. | Y |
| Aidan Pucchio 2022 | P7 | Desire to learn more about artificial intelligence | Author-reported proportion; response scale not reported in the source article | Not clearly reported | Author-reported proportion; numerator is the endorsed response category | Neutral handling not explicitly reported; denominator retained as reported | 360 | 462 | 0.7792 | Mapped to P7 (Willingness to learn or adopt AI) under operational definition: Proportion expressing willingness, readiness, or intention to learn about AI, use AI tools, or integrate AI into their future practice. | Y |
| Charlotte Blease 2022 | P7 | Plan to learn about AI or machine learning in medicine | Likert agreement scale; point count not reported in the source article | Not clearly reported | Agree + Somewhat agree + Strongly agree vs all other item responses | Neutral responses, if available, were excluded from numerator and retained in denominator | 99 | 241 | 0.4108 | Mapped to P7 (Willingness to learn or adopt AI) under operational definition: Proportion expressing willingness, readiness, or intention to learn about AI, use AI tools, or integrate AI into their future practice. | Y |
| Elena A Wood 2021 | P7 | Willingness to adopt new technology in medical education | response scale not reported in the source article | Not reported | Dichotomization method not reported in primary study; proportion extracted from reported percentage | Neutral handling not explicitly reported; denominator retained as reported | 103 | 117 | 0.8803 | Mapped to P7 (Willingness to learn or adopt AI) under operational definition: Proportion expressing willingness, readiness, or intention to learn about AI, use AI tools, or integrate AI into their future practice. | Y |
| Xi Cao 2026 | P8 | Concern about responsibility for patient safety when using GenAI | response scale not reported in the source article | Not reported | Dichotomization method not reported in primary study; proportion extracted from reported percentage | Neutral handling not explicitly reported; denominator retained as reported | 697 | 1062 | 0.6563 | Mapped to P8 (Ethical concerns about AI in healthcare) under operational definition: Proportion expressing concern about AI ethics: privacy, bias, accountability, transparency, patient safety, or informed consent. | Y |
| Ali Veysel Kara 2026 | P8 | At least one ethical concern about AI in medicine | Author-reported categorical response/proportion; exact response options not fully reported | Not fully reported | Author-reported proportion; numerator is the endorsed response category | Neutral handling not explicitly reported; denominator retained as reported | 907 | 1346 | 0.6738 | Mapped to P8 (Ethical concerns about AI in healthcare) under operational definition: Proportion expressing concern about AI ethics: privacy, bias, accountability, transparency, patient safety, or informed consent. | Y |
| Yacoub A. Yousef 2026 | P8 | Ethical concern about bias in AI | Author-reported categorical response/proportion; exact response options not fully reported | Not fully reported | Author-reported proportion; numerator is the endorsed response category | Neutral handling not explicitly reported; denominator retained as reported | 246 | 353 | 0.6969 | Mapped to P8 (Ethical concerns about AI in healthcare) under operational definition: Proportion expressing concern about AI ethics: privacy, bias, accountability, transparency, patient safety, or informed consent. | Y |
| Na Hu 2025 | P8 | Concern about ChatGPT misinformation or plagiarism | Author-reported proportion; response scale not reported in the source article | Not clearly reported | Author-reported proportion; numerator is the endorsed response category | Neutral handling not explicitly reported; denominator retained as reported | 871 | 1133 | 0.7688 | Mapped to P8 (Ethical concerns about AI in healthcare) under operational definition: Proportion expressing concern about AI ethics: privacy, bias, accountability, transparency, patient safety, or informed consent. | Y |
| Omar A. Ghanem 2025 | P8 | Ethical and privacy concerns about AI | Author-reported proportion; response scale not reported in the source article | Not clearly reported | Author-reported proportion; numerator is the endorsed response category | Neutral handling not explicitly reported; denominator retained as reported | 284 | 423 | 0.6714 | Mapped to P8 (Ethical concerns about AI in healthcare) under operational definition: Proportion expressing concern about AI ethics: privacy, bias, accountability, transparency, patient safety, or informed consent. | Y |
| Anna Fußhöller 2025 | P8 | Concern about misinformation from ChatGPT | Author-reported proportion; response scale not reported in the source article | Not clearly reported | Author-reported proportion; numerator is the endorsed response category | Neutral handling not explicitly reported; denominator retained as reported | 55 | 75 | 0.7333 | Mapped to P8 (Ethical concerns about AI in healthcare) under operational definition: Proportion expressing concern about AI ethics: privacy, bias, accountability, transparency, patient safety, or informed consent. | Y |
| Safia Elwaleed Elhassan 2025 | P8 | Ethical concerns about ChatGPT academic integrity, privacy, or confidentiality | response scale not reported in the source article | Not reported | Dichotomization method not reported in primary study; proportion extracted from reported percentage | Neutral handling not explicitly reported; denominator retained as reported | 226 | 293 | 0.7713 | Mapped to P8 (Ethical concerns about AI in healthcare) under operational definition: Proportion expressing concern about AI ethics: privacy, bias, accountability, transparency, patient safety, or informed consent. | Y |
| Cecilia Tran 2025 | P8 | Concern about generative AI accuracy, bias, or stigma | Author-reported categorical response/proportion; exact response options not fully reported | Not fully reported | Author-reported proportion; numerator is the endorsed response category | Neutral handling not explicitly reported; denominator retained as reported | 131 | 166 | 0.7892 | Mapped to P8 (Ethical concerns about AI in healthcare) under operational definition: Proportion expressing concern about AI ethics: privacy, bias, accountability, transparency, patient safety, or informed consent. | Y |
| Bayan Almehmadi 2025 | P8 | Ethical concern about AI privacy, misinformation, or dehumanization | Author-reported proportion; response scale not reported in the source article | Not clearly reported | Author-reported proportion; numerator is the endorsed response category | Neutral handling not explicitly reported; denominator retained as reported | 99 | 170 | 0.5824 | Mapped to P8 (Ethical concerns about AI in healthcare) under operational definition: Proportion expressing concern about AI ethics: privacy, bias, accountability, transparency, patient safety, or informed consent. | Y |
| Zahra Arab-Borzu 2025 | P8 | Concern about AI risks, bias, and ethical or legal issues | response scale not reported in the source article | Not reported | Dichotomization method not reported in primary study; proportion extracted from reported percentage | Neutral handling not explicitly reported; denominator retained as reported | 169 | 279 | 0.6057 | Mapped to P8 (Ethical concerns about AI in healthcare) under operational definition: Proportion expressing concern about AI ethics: privacy, bias, accountability, transparency, patient safety, or informed consent. | Y |
| Florina Onetiu 2025 | P8 | Concern about AI data security and clinical responsibility | response scale not reported in the source article | Not reported | Dichotomization method not reported in primary study; proportion extracted from reported percentage | Neutral handling not explicitly reported; denominator retained as reported | 115 | 161 | 0.7143 | Mapped to P8 (Ethical concerns about AI in healthcare) under operational definition: Proportion expressing concern about AI ethics: privacy, bias, accountability, transparency, patient safety, or informed consent. | Y |
| Ahmed Samir Abdelhafiz 2025 | P8 | Concern about privacy risks associated with ChatGPT | 5-point Likert agreement scale: strongly disagree to strongly agree | 5 | Author-reported proportion; numerator is the endorsed response category | Neutral response category reported; not counted in numerator and retained in denominator unless source denominator was item-specific | 438 | 614 | 0.7134 | Mapped to P8 (Ethical concerns about AI in healthcare) under operational definition: Proportion expressing concern about AI ethics: privacy, bias, accountability, transparency, patient safety, or informed consent. | Y |
| Mingchan Liu 2026 | P8 | Concern about privacy breaches during AI tool use | Likert agreement scale; point count not reported in the source article | Not clearly reported | Author-reported proportion; numerator is the endorsed response category | Neutral response category reported; not counted in numerator and retained in denominator unless source denominator was item-specific | 382 | 788 | 0.4848 | Mapped to P8 (Ethical concerns about AI in healthcare) under operational definition: Proportion expressing concern about AI ethics: privacy, bias, accountability, transparency, patient safety, or informed consent. | Y |
| Poorna Fernando 2025 | P8 | Ethical concerns about AI in healthcare | response scale not reported in the source article | Not reported | Dichotomization method not reported in primary study; proportion extracted from reported percentage | Neutral handling not explicitly reported; denominator retained as reported | 182 | 304 | 0.5987 | Mapped to P8 (Ethical concerns about AI in healthcare) under operational definition: Proportion expressing concern about AI ethics: privacy, bias, accountability, transparency, patient safety, or informed consent. | Y |
| Juan José Gualda-Gea 2025 | P8 | Concern about moral implications of integrating AI tools | Author-reported proportion; response scale not reported in the source article | Not clearly reported | Author-reported proportion; numerator is the endorsed response category | Neutral handling not explicitly reported; denominator retained as reported | 14 | 29 | 0.4828 | Mapped to P8 (Ethical concerns about AI in healthcare) under operational definition: Proportion expressing concern about AI ethics: privacy, bias, accountability, transparency, patient safety, or informed consent. | Y |
| Aleksandra Ignjatović 2025 | P8 | Concern about academic integrity in AI use | response scale not reported in the source article | Not reported | Dichotomization method not reported in primary study; proportion extracted from reported percentage | Neutral handling not explicitly reported; denominator retained as reported | 286 | 511 | 0.5597 | Mapped to P8 (Ethical concerns about AI in healthcare) under operational definition: Proportion expressing concern about AI ethics: privacy, bias, accountability, transparency, patient safety, or informed consent. | Y |
| Faiza Ejas 2025 | P8 | Acknowledgement of ethical challenges of large language models in research | Author-reported categorical response/proportion; exact response options not fully reported | Not fully reported | Author-reported proportion; numerator is the endorsed response category | Neutral handling not explicitly reported; denominator retained as reported | 581 | 1180 | 0.4924 | Mapped to P8 (Ethical concerns about AI in healthcare) under operational definition: Proportion expressing concern about AI ethics: privacy, bias, accountability, transparency, patient safety, or informed consent. | Y |
| Robbi Miguel G. Falcon 2025 | P8 | Concern about AI data privacy, manipulation, confidentiality, and transparency | 5-point Likert agreement scale: strongly disagree to strongly agree | 5 | Author-reported proportion; numerator is the endorsed response category | Neutral handling not explicitly reported; denominator retained as reported | 16 | 20 | 0.8000 | Mapped to P8 (Ethical concerns about AI in healthcare) under operational definition: Proportion expressing concern about AI ethics: privacy, bias, accountability, transparency, patient safety, or informed consent. | Y |
| Muhammad Farooq and Usmani (2025) | P8 | Ethical concern about AI data privacy, bias, and transparency | Author-reported proportion; response scale not reported in the source article | Not clearly reported | Author-reported proportion; numerator is the endorsed response category | Neutral handling not explicitly reported; denominator retained as reported | 86 | 152 | 0.5658 | Mapped to P8 (Ethical concerns about AI in healthcare) under operational definition: Proportion expressing concern about AI ethics: privacy, bias, accountability, transparency, patient safety, or informed consent. | N |
| Wenjuan Tao 2024 | P8 | Concern about data privacy breaches and misdiagnosis risks | Author-reported categorical response/proportion; exact response options not fully reported | Not fully reported | Author-reported proportion; numerator is the endorsed response category | Neutral handling not explicitly reported; denominator retained as reported | 635 | 693 | 0.9163 | Mapped to P8 (Ethical concerns about AI in healthcare) under operational definition: Proportion expressing concern about AI ethics: privacy, bias, accountability, transparency, patient safety, or informed consent. | Y |
| Adnan Abu Hammour 2024 | P8 | Concern about ChatGPT inaccuracies and reliability | Author-reported proportion; response scale not reported in the source article | Not clearly reported | Author-reported proportion; numerator is the endorsed response category | Neutral handling not explicitly reported; denominator retained as reported | 191 | 244 | 0.7828 | Mapped to P8 (Ethical concerns about AI in healthcare) under operational definition: Proportion expressing concern about AI ethics: privacy, bias, accountability, transparency, patient safety, or informed consent. | Y |
| Zainudheen Faroog 2024 | P8 | Concern about potential patient harm from ophthalmology AI | Author-reported proportion; response scale not reported in the source article | Not clearly reported | Author-reported proportion; numerator is the endorsed response category | Neutral response category reported; not counted in numerator and retained in denominator unless source denominator was item-specific | 8 | 345 | 0.0232 | Mapped to P8 (Ethical concerns about AI in healthcare) under operational definition: Proportion expressing concern about AI ethics: privacy, bias, accountability, transparency, patient safety, or informed consent. | Y |
| Mohammed Hammad Jaber Amin 2024 | P8 | Concern about ethical implications of AI in medical practice | Author-reported proportion; response scale not reported in the source article | Not clearly reported | Author-reported proportion; numerator is the endorsed response category | Neutral handling not explicitly reported; denominator retained as reported | 285 | 762 | 0.3740 | Mapped to P8 (Ethical concerns about AI in healthcare) under operational definition: Proportion expressing concern about AI ethics: privacy, bias, accountability, transparency, patient safety, or informed consent. | Y |
| Preetha Jackson 2024 | P8 | Concern that AI could reduce the humanistic aspect of medicine | 5-point Likert agreement scale: strongly disagree to strongly agree | 5 | Author-reported proportion; numerator is the endorsed response category | Neutral handling not explicitly reported; denominator retained as reported | 174 | 325 | 0.5354 | Mapped to P8 (Ethical concerns about AI in healthcare) under operational definition: Proportion expressing concern about AI ethics: privacy, bias, accountability, transparency, patient safety, or informed consent. | Y |
| Lukas Weidener 2024 | P8 | Ethical concern about AI in medicine | Author-reported proportion; response scale not reported in the source article | Not clearly reported | Author-reported proportion; numerator is the endorsed response category | Neutral handling not explicitly reported; denominator retained as reported | 464 | 487 | 0.9528 | Mapped to P8 (Ethical concerns about AI in healthcare) under operational definition: Proportion expressing concern about AI ethics: privacy, bias, accountability, transparency, patient safety, or informed consent. | Y |
| Anamaria Malešević 2024 | P8 | Concern about informed consent in AI decision-making | response scale not reported in the source article | Not reported | Dichotomization method not reported in primary study; proportion extracted from reported percentage | Neutral handling not explicitly reported; denominator retained as reported | 1005 | 1701 | 0.5908 | Mapped to P8 (Ethical concerns about AI in healthcare) under operational definition: Proportion expressing concern about AI ethics: privacy, bias, accountability, transparency, patient safety, or informed consent. | Y |
| Khaldoon Al-Roomi 2024 | P8 | Ethical issues in using AI applications in medical studies | Author-reported proportion; response scale not reported in the source article | Not clearly reported | Author-reported proportion; numerator is the endorsed response category | Neutral handling not explicitly reported; denominator retained as reported | 29 | 151 | 0.1921 | Mapped to P8 (Ethical concerns about AI in healthcare) under operational definition: Proportion expressing concern about AI ethics: privacy, bias, accountability, transparency, patient safety, or informed consent. | Y |
| Saif M I Alkhaaldi 2023 | P8 | Concern that AI could reduce humanism and patient trust | Author-reported categorical response/proportion; exact response options not fully reported | Not fully reported | Author-reported proportion; numerator is the endorsed response category | Neutral response category reported; not counted in numerator and retained in denominator unless source denominator was item-specific | 168 | 265 | 0.6340 | Mapped to P8 (Ethical concerns about AI in healthcare) under operational definition: Proportion expressing concern about AI ethics: privacy, bias, accountability, transparency, patient safety, or informed consent. | Y |
| Kehinde Sunday Oluwadiya 2023 | P8 | Concern that AI could dehumanise health care | Author-reported proportion; response scale not reported in the source article | Not clearly reported | Author-reported proportion; numerator is the endorsed response category | Neutral handling not explicitly reported; denominator retained as reported | 488 | 691 | 0.7062 | Mapped to P8 (Ethical concerns about AI in healthcare) under operational definition: Proportion expressing concern about AI ethics: privacy, bias, accountability, transparency, patient safety, or informed consent. | Y |
| Ali Veysel Kara 2026 | P9 | Reliability of AI-generated information | Likert agreement scale; point count not reported in the source article | Not clearly reported | Dichotomization method not reported in primary study; proportion extracted from reported percentage | Neutral handling not explicitly reported; denominator retained as reported | 532 | 1346 | 0.3952 | Mapped to P9 (Trust in AI-assisted clinical decisions) under operational definition: Proportion expressing trust or confidence in AI-generated clinical recommendations, diagnoses, or treatment plans. | Y |
| Sumit Prasad 2026 | P9 | Trust that clinical AI will be more accurate than physicians | 5-point Likert agreement scale: strongly disagree to strongly agree | 5 | Agree + Strongly agree vs all other item responses | Neutral responses, if available, were excluded from numerator and retained in denominator | 33 | 317 | 0.1041 | Mapped to P9 (Trust in AI-assisted clinical decisions) under operational definition: Proportion expressing trust or confidence in AI-generated clinical recommendations, diagnoses, or treatment plans. | Y |
| Omar A. Ghanem 2025 | P9 | Trust in AI-generated medical diagnoses | Author-reported proportion; response scale not reported in the source article | Not clearly reported | Author-reported proportion; numerator is the endorsed response category | Neutral handling not explicitly reported; denominator retained as reported | 188 | 423 | 0.4444 | Mapped to P9 (Trust in AI-assisted clinical decisions) under operational definition: Proportion expressing trust or confidence in AI-generated clinical recommendations, diagnoses, or treatment plans. | Y |
| Anna Fußhöller 2025 | P9 | Trust in ChatGPT outputs | Author-reported categorical response/proportion; exact response options not fully reported | Not fully reported | Author-reported proportion; numerator is the endorsed response category | Neutral handling not explicitly reported; denominator retained as reported | 35 | 84 | 0.4167 | Mapped to P9 (Trust in AI-assisted clinical decisions) under operational definition: Proportion expressing trust or confidence in AI-generated clinical recommendations, diagnoses, or treatment plans. | Y |
| Abdul Sami 2025 | P9 | Trust in AI-provided learning and medical information | 5-point Likert agreement scale: strongly disagree to strongly agree | 5 | Author-reported proportion; numerator is the endorsed response category | Neutral handling not explicitly reported; denominator retained as reported | 410 | 702 | 0.584 | Mapped to P9 (Trust in AI-assisted clinical decisions) under operational definition: Proportion expressing trust or confidence in AI-generated clinical recommendations, diagnoses, or treatment plans. | N |
| Ahmed Samir Abdelhafiz 2025 | P9 | Trust in technology such as ChatGPT | 5-point Likert agreement scale: strongly disagree to strongly agree | 5 | Author-reported proportion; numerator is the endorsed response category | Neutral response category reported; not counted in numerator and retained in denominator unless source denominator was item-specific | 296 | 614 | 0.4821 | Mapped to P9 (Trust in AI-assisted clinical decisions) under operational definition: Proportion expressing trust or confidence in AI-generated clinical recommendations, diagnoses, or treatment plans. | Y |
| Juan José Gualda-Gea 2025 | P9 | Trust concern related to biases in AI tools | Author-reported proportion; response scale not reported in the source article | Not clearly reported | Author-reported proportion; numerator is the endorsed response category | Neutral handling not explicitly reported; denominator retained as reported | 17 | 29 | 0.5862 | Mapped to P9 (Trust in AI-assisted clinical decisions) under operational definition: Proportion expressing trust or confidence in AI-generated clinical recommendations, diagnoses, or treatment plans. | Y |
| Alan Y. Xu 2024 | P9 | Perceived accuracy of information from large language models | Author-reported categorical response/proportion; exact response options not fully reported | Not fully reported | Author-reported proportion; numerator is the endorsed response category | Neutral handling not explicitly reported; denominator retained as reported | 54 | 70 | 0.7714 | Mapped to P9 (Trust in AI-assisted clinical decisions) under operational definition: Proportion expressing trust or confidence in AI-generated clinical recommendations, diagnoses, or treatment plans. | Y |
| Pasin Tangadulrat 2023 | P9 | Perceived evidence basis of AI-generated public health answers | Author-reported proportion; response scale not reported in the source article | Not clearly reported | Author-reported proportion; numerator is the endorsed response category | Neutral handling not explicitly reported; denominator retained as reported | 110 | 124 | 0.8871 | Mapped to P9 (Trust in AI-assisted clinical decisions) under operational definition: Proportion expressing trust or confidence in AI-generated clinical recommendations, diagnoses, or treatment plans. | Y |

**Table S12. Verification of harmonized study-domain contributions against source articles.**

| Study (first author, year) | Domain | Events | Total | Source | Locator | Brief justification |
| --- | --- | --- | --- | --- | --- | --- |
| Ali Veysel Kara 2026 | P6 | 934 | 1346 | Source article | p. 9, Awareness and Use of Artificial Intelligence | 30.6% never used AI among n=1346, so any use = 69.4%, matching 934/1346 after rounding. |
| Ali Veysel Kara 2026 | P9 | 532 | 1346 | Source article | Table 3, p. 19, Q19 | 404 + 128 = 532 of n=1346. |
| Rixiang Xu 2026 | P6 | 654 | 661 | Source article | Table 1, p. 4 | 661 - 7 never = 654 any use. |
| Yacoub A. Yousef 2026 | P7 | 277 | 353 | Source article | Table 2, p. 4 | Medical-student-specific count 277/353. |
| Na Hu 2025 | P8 | 871 | 1133 | Source article | p. 4, misinformation concern result | 76.9% of n=1133 corresponds to 871. |
| Omar A. Ghanem 2025 | P6 | 318 | 423 | Source article and supplementary material | Table 6, row 5 | 423 - 105 never = 318 any use. |
| Omar A. Ghanem 2025 | P8 | 284 | 423 | Source article and supplementary material | Table 5, row 10 | 202 agree + 82 strongly agree = 284. |
| Omar A. Ghanem 2025 | P9 | 188 | 423 | Source article and supplementary material | Supplement Table 13; main article p. 4 | Source reports 44.4%; phase counts 35 + 153 = 188. |
| Safia Elwaleed Elhassan 2025 | P7 | 149 | 293 | Source article | Table 3, p. 4 | Agree 149, neutral 96, disagree 48. |
| Safia Elwaleed Elhassan 2025 | P8 | 226 | 293 | Source article | Table 3, p. 4 | Agree 226 (77.1%). |
| Bayan Almehmadi 2025 | P8 | 99 | 170 | Source article | Table 4, p. 6 | 76 agree + 23 strongly agree = 99. |
| Zahra Arab-Borzu 2025 | P7 | 229 | 280 | Source article | Table 4, p. 4 | 106 + 123 = 229. |
| Zahra Arab-Borzu 2025 | P8 | 169 | 279 | Source article | Table 6, p. 5 | Item-specific denominator is 279; event = 169. |
| Florina Onetiu 2025 | P8 | 115 | 161 | Source article | Table 2 | 71.40% agreement among n=161 corresponds to 115. |
| Samah Alfahl 2025 | P4 | 88 | 189 | Source article | p. 3, Q16 | 46.48% of n=189 corresponds to 88. |
| Min Zhang 2025 | P6 | 241 | 254 | Source article | frequency of AILM use result | 254 - 13 never = 241 any use. |
| Abdul Sami 2025 | P6 | 416 | 702 | Source article | p. 6 | Value verified; study excluded from the 96-study synthesis. |
| Muhammad Farooq and Usmani (2025) | P1 | 111 | 152 | Source article | Reported AI-education results | Value verified; study excluded from the 96-study synthesis. |
| Ahmed Samir Abdelhafiz 2025 | P6 | 482 | 614 | Source article | p. 3; Table 1 | Yes 482 (78.5%). |
| Ahmed Samir Abdelhafiz 2025 | P7 | 441 | 614 | Source article | p. 5 | Agree 441, neutral 132, disagree 41. |
| Mingchan Liu 2026 | P6 | 720 | 788 | Source article | p. 5, text below Figure 2 | 91.24% of n=788 corresponds to 720. |
| Shuo Duan 2025 | P7 | 253 | 553 | Source article | p. 4; Supplement Table 9 | 45.75% of n=553 corresponds to 253. |
| Hamdah Hanifa 2025 | P6 | 157 | 564 | Source article | Table 1, p. 4 | Experience with AI: yes 157 (27.8%), no 407. |
| Poorna Fernando 2025 | P8 | 182 | 304 | Source article | p. 3 | 60% of n=304 corresponds to 182. |
| Juan Jose Gualda-Gea 2025 | P7 | 24 | 29 | Source article | p. 5, Influence on Specialty Choice | 24 (83%). |
| Aleksandra Ignjatovic 2025 | P8 | 286 | 511 | Source article and supplementary material | Table 2, row 20 | 286 (56.0%) among students who used ChatGPT, n=511. |
| Faiza Ejas 2025 | P1 | 608 | 1180 | Source article | p. 4 | 608 participants (52%) supported LLM use in healthcare. |
| Khalid S. Alharkan 2025 | P6 | 320 | 525 | Source article | p. 3, Figure 1 | 61% of n=525 corresponds to 320. |
| Abeer F. Almarzouki 2025 | P7 | 229 | 354 | Source article | Table 1, p. 3 | 137 agree + 92 strongly agree = 229. |
| Adnan Abu Hammour 2024 | P6 | 177 | 244 | Source article | p. 5/14 | 72.5% any use among n=244 corresponds to 177. |
| Mohammed Hammad Jaber Amin 2024 | P1 | 603 | 762 | Source article | Table item on AI essential in medical field | 366 agree + 237 strongly agree = 603. |
| Mohammed Hammad Jaber Amin 2024 | P6 | 267 | 762 | Source article | p. 5 | 267 (35%) had ever practically applied AI. |
| Mohammed Hammad Jaber Amin 2024 | P8 | 285 | 762 | Source article | Table 4 | 186 agree + 99 strongly agree = 285. |
| Lukas Weidener 2024 | P7 | 404 | 487 | Source article and supplementary material | Table S2, S20 | 320 + 84 = 404. |
| Anamaria Malesevic 2024 | P7 | 762 | 1701 | Source article | p. 5 | 44.8% of n=1701 corresponds to 762. |
| Anamaria Malesevic 2024 | P8 | 1005 | 1701 | Source article | abstract; p. 1 | 59.1% of n=1701 corresponds to 1005. |
| Rohankumar Gandhi 2024 | P7 | 444 | 605 | Source article | Table 5, p. 6 | 181/201 postgraduates + 263/404 undergraduates = 444/605. |
| Rohankumar Gandhi 2024 | P6 | 278 | 605 | Source article | Table 5, p. 6 | 141 postgraduates + 137 undergraduates = 278/605. |
| Khaldoon Al-Roomi 2024 | P8 | 29 | 151 | Source article | Table 3, p. 5 | Table reports Yes 29 (19.2%) and No 122 (80.8%); the Yes response maps to having an ethical concern. |
| Pasin Tangadulrat 2023 | P9 | 110 | 124 | Source article and supplementary material | Multimedia Appendix 2, Figure 2A; article p. 3 | Medical-student positive perception is 88.7%; 110/124 matches source rounding. |
| Kehinde Sunday Oluwadiya 2023 | P8 | 488 | 691 | Source article | Table 5, p. 7 | Student row reports Yes 488 (70.6%), No 123, and Maybe 80; item counts sum to 691. |
| Elena A Wood 2021 | P7 | 103 | 117 | Source article | Table 1, p. 3 | 33 AI in patient care + 70 both = 103 of 117 students. |

Of the 42 lower-confidence rows re-checked against source articles, 38 were confirmed and 4 were corrected. The 4 corrections were: S11 P9 (trust) 178/423→188/423; S48 P7 230/354→229/354; S74 P8 122/152→29/151; S78 P8 500/708→488/691.

**Table S13. Feasibility-screened exploratory meta-regressions that were run.**

| **Domain** | **Moderator** | **k** | **P value** | **Moderator levels (k)** |
| --- | --- | --- | --- | --- |
| P1 | Publication period | 44 | .976 | 2023 onward (k=37); pre-2023 (k=7) |
| P1 | AI type | 44 | .005 | Broad/general AI (k=22); chatGPT/LLM or generative AI (k=14); domain-specific AI (k=8) |
| P1 | JBI risk of bias | 44 | .943 | Low (k=4); moderate (k=40) |
| P2 | AI type | 16 | .040 | Broad/general AI (k=8); chatGPT/LLM or generative AI (k=5); domain-specific AI (k=3) |
| P2 | JBI risk of bias | 16 | .714 | Low (k=4); moderate (k=12) |
| P3 | Publication period | 38 | .404 | 2023 onward (k=33); pre-2023 (k=5) |
| P3 | AI type | 38 | .322 | Broad/general AI (k=22); chatGPT/LLM or generative AI (k=8); domain-specific AI (k=8) |
| P3 | JBI risk of bias | 38 | .135 | Low (k=7); moderate (k=31) |
| P4 | Publication period | 32 | .174 | 2023 onward (k=26); pre-2023 (k=6) |
| P4 | AI type | 32 | .013 | Broad/general AI (k=17); chatGPT/LLM or generative AI (k=6); domain-specific AI (k=9) |
| P4 | JBI risk of bias | 32 | .171 | Low (k=4); moderate (k=28) |
| P5 | Publication period | 52 | .043 | 2023 onward (k=44); pre-2023 (k=8) |
| P5 | AI type | 52 | .008 | Broad/general AI (k=30); chatGPT/LLM or generative AI (k=14); domain-specific AI (k=8) |
| P5 | JBI risk of bias | 52 | .894 | Low (k=8); moderate (k=44) |
| P6 | AI type | 36 | .306 | Broad/general AI (k=18); chatGPT/LLM or generative AI (k=18) |
| P6 | JBI risk of bias | 36 | .101 | Low (k=8); moderate (k=28) |
| P7 | Publication period | 22 | .936 | 2023 onward (k=19); pre-2023 (k=3) |
| P7 | World Bank income | 22 | .651 | High income (k=11); low/lower-middle income (k=6); upper-middle income (k=5) |
| P7 | JBI risk of bias | 22 | .656 | Low (k=5); moderate (k=17) |
| P8 | JBI risk of bias | 28 | .723 | Low (k=6); moderate (k=22) |

All meta-regressions are exploratory; P values are nominal and not adjusted for multiplicity, and do not constitute confirmatory or causal evidence.

**Table S14. Meta-regressions not run and reasons.**

| **Domain** | **Moderator** | **k** | **Status** | **Reason** |
| --- | --- | --- | --- | --- |
| P1 | World Bank income | 44 | Not performed | Fewer than 3 studies in at least 1 moderator level: Mixed income |
| P2 | Publication period | 16 | Not performed | Fewer than 3 studies in at least 1 moderator level: Pre-2023 |
| P2 | World Bank income | 16 | Not performed | Fewer than 3 studies in at least 1 moderator level: Low/lower-middle income; Mixed income |
| P3 | World Bank income | 38 | Not performed | Fewer than 3 studies in at least 1 moderator level: Mixed income |
| P4 | World Bank income | 32 | Not performed | Fewer than 3 studies in at least 1 moderator level: Mixed income |
| P5 | World Bank income | 52 | Not performed | Fewer than 3 studies in at least 1 moderator level: Mixed income |
| P6 | Publication period | 36 | Not performed | Fewer than 3 studies in at least 1 moderator level: Pre-2023 |
| P6 | World Bank income | 36 | Not performed | Fewer than 3 studies in at least 1 moderator level: Mixed income |
| P7 | AI type | 22 | Not performed | Fewer than 3 studies in at least 1 moderator level: Domain-specific AI |
| P8 | Publication period | 28 | Not performed | Only one moderator level |
| P8 | World Bank income | 28 | Not performed | Fewer than 3 studies in at least 1 moderator level: Mixed income |
| P8 | AI type | 28 | Not performed | Fewer than 3 studies in at least 1 moderator level: Domain-specific AI |
| P9 | Publication period | 8 | Not performed | k < 10 |
| P9 | World Bank income | 8 | Not performed | k < 10 |
| P9 | AI type | 8 | Not performed | k < 10 |
| P9 | JBI risk of bias | 8 | Not performed | k < 10 |

All meta-regressions are exploratory; P values are nominal and not adjusted for multiplicity, and do not constitute confirmatory or causal evidence.

**Table S15. Leave-one-out sensitivity analyses for all 9 proportion domains.**

| **Domain** | **k** | **Primary pooled %** | **Leave-one-out pooled range %** | **Maximum shift, percentage points** | **Interpretation** |
| --- | --- | --- | --- | --- | --- |
| P1: Positive attitude | 44 | 76.9% | 76.1-77.7 | 0.8 | No single-study omission changed the overall interpretation |
| P2: Career benefit | 16 | 78.4% | 77.2-81.2 | 2.8 | No single-study omission changed the overall interpretation |
| P3: Curricular integration | 38 | 76.6% | 75.6-77.5 | 1.0 | No single-study omission changed the overall interpretation |
| P4: Replacement concern | 32 | 39.9% | 38.7-40.9 | 1.3 | No single-study omission changed the overall interpretation |
| P5: Familiarity/knowledge | 52 | 63.3% | 62.1-64.4 | 1.1 | No single-study omission changed the overall interpretation |
| P6: Prior AI use | 36 | 63.2% | 61.6-64.9 | 1.7 | No single-study omission changed the overall interpretation |
| P7: Willingness to adopt | 22 | 71.5% | 70.2-72.9 | 1.3 | No single-study omission changed the overall interpretation |
| P8: Ethical concerns | 28 | 62.8% | 61.2-65.6 | 2.8 | No single-study omission changed the overall interpretation |
| P9: Trust in AI | 8 | 50.6% | 44.1-57.3 | 6.7 | No single-study omission changed the overall interpretation |

*Leave-one-out values were calculated from the final 96-study dataset and covered the four retained studies flagged in the participant-independence audit. No separate simultaneous-exclusion analysis was performed. Maximum shift is the largest absolute change from the primary pooled estimate.*

**Table S16. Egger regression test results for small-study effects across proportion domains where feasible.**

| **Domain** | **k** | **Status** | **Egger P value** | **Interpretation** |
| --- | --- | --- | --- | --- |
| P1: Positive attitude | 44 | Performed | <.001 | Small-study effects detected; interpret cautiously |
| P2: Career benefit | 16 | Performed | .129 | No statistically significant funnel-plot asymmetry was detected; this does not exclude small-study effects. |
| P3: Curricular integration | 38 | Performed | <.001 | Small-study effects detected; interpret cautiously |
| P4: Replacement concern | 32 | Performed | .461 | No statistically significant funnel-plot asymmetry was detected; this does not exclude small-study effects. |
| P5: Familiarity/knowledge | 52 | Performed | <.001 | Small-study effects detected; interpret cautiously |
| P6: Prior AI use | 36 | Performed | <.001 | Small-study effects detected; interpret cautiously |
| P7: Willingness to adopt | 22 | Performed | <.001 | Small-study effects detected; interpret cautiously |
| P8: Ethical concerns | 28 | Performed | .475 | No statistically significant funnel-plot asymmetry was detected; this does not exclude small-study effects. |
| P9: Trust in AI | 8 | Not performed (k<10) | Not performed | Egger test not run because k < 10 |

*Following Sterne et al, funnel plot asymmetry is referred to as small-study effects rather than publication bias, as asymmetry may arise from multiple sources. P9 was not tested because k<10.*

**Table S17. Detailed GRADE certainty assessment for the 9 proportional self-report domains.**

| **Outcome** | **Participants (studies); design** | **Pooled absolute proportion, % (95% CI); 95% PI; I²; τ²** | **Risk of bias** | **Inconsistency** | **Indirectness** | **Imprecision** | **Other considerations** | **Certainty** |
| --- | --- | --- | --- | --- | --- | --- | --- | --- |
| P1: Positive attitude | 20,806 (44); cross-sectional | 76.9 (72.2-81.4); PI 42.2-98.3; I²=98.3%; τ²=0.031 | Seriousᵃ | Seriousᵇ | Seriousᶜ | Seriousᵈ | Small-study effects detectedᶠ | ⊕○○○  Very low |
| P2: Career benefit | 9,799 (16); cross-sectional | 78.4 (69.5-86.2); PI 45.3-98.3; I²=98.0%; τ²=0.025 | Seriousᵃ | Seriousᵇ | Seriousᶜ | Seriousᵈ | No additional concern from feasible Egger testᵍ | ⊕○○○  Very low |
| P3: Curricular integration | 16,308 (38); cross-sectional | 76.6 (71.8-81.1); PI 47.8-96.1; I²=97.2%; τ²=0.021 | Seriousᵃ | Seriousᵇ | Seriousᶜ | Seriousᵈ | Small-study effects detectedᶠ | ⊕○○○  Very low |
| P4: Replacement concern | 16,642 (32); cross-sectional | 39.9 (33.6-46.5); PI 6.6-80.1; I²=98.8%; τ²=0.042 | Seriousᵃ | Seriousᵇ | Seriousᶜ | Seriousᵈ | No additional concern from feasible Egger testᵍ | ⊕○○○  Very low |
| P5: Familiarity/knowledge | 27,817 (52); cross-sectional | 63.3 (55.9-70.3); PI 7.8-100.0; I²=99.5%; τ²=0.097 | Seriousᵃ | Seriousᵇ | Seriousᶜ | Seriousᵈ | Small-study effects detectedᶠ | ⊕○○○  Very low |
| P6: Prior AI use | 17,374 (36); cross-sectional | 63.2 (53.2-72.6); PI 8.5-100.0; I²=99.4%; τ²=0.091 | Seriousᵃ | Seriousᵇ | Seriousᶜ | Seriousᵈ | Small-study effects detectedᶠ | ⊕○○○  Very low |
| P7: Willingness to learn/adopt | 9,199 (22); cross-sectional | 71.5 (64.8-77.8); PI 37.9-95.4; I²=97.7%; τ²=0.026 | Seriousᵃ | Seriousᵇ | Seriousᶜ | Seriousᵈ | Small-study effects detectedᶠ | ⊕○○○  Very low |
| P8: Ethical concerns | 14,571 (28); cross-sectional | 62.8 (53.9-71.3); PI 21.9-95.0; I²=98.8%; τ²=0.042 | Seriousᵃ | Seriousᵇ | Seriousᶜ | Seriousᵈ | No additional concern from feasible Egger testᵍ | ⊕○○○  Very low |
| P9: Trust in AI-assisted decisions | 3,007 (8); cross-sectional | 50.6 (28.5-72.6); PI 7.3-93.3; I²=98.1%; τ²=0.041 | Seriousᵃ | Seriousᵇ | Seriousᶜ | Seriousᵉ | Egger test not performed (k<10)ʰ | ⊕○○○  Very low |

Table S17 is the detailed GRADE evidence profile supporting the reader-facing Summary of Findings table in the main text. All outcomes were based on cross-sectional self-report studies, and certainty was rated for the pooled absolute proportion. ᵃ Serious risk of bias: convenience or voluntary sampling, unclear response-rate denominators, frequent use of nonvalidated or adapted instruments, and self-selection; 79 of 96 studies were review-defined moderate risk. ᵇ Serious inconsistency: 95% prediction intervals were very wide across all domains and forest plots showed marked between-study dispersion; I² is reported as a complementary model statistic but was not used to quantify the practical magnitude of heterogeneity. ᶜ Serious indirectness: survey items and AI referents were non-equivalent and required operational harmonization. ᵈ Serious imprecision: wide prediction intervals indicate substantial uncertainty about the value expected in a new comparable setting despite large aggregate sample sizes. ᵉ For P9, only 8 studies contributed and both the confidence interval and prediction interval were wide. ᶠ Egger regression indicated small-study effects; this was treated as a possible publication-bias signal, not as proof of publication bias, because funnel asymmetry may also arise from heterogeneity and outcome-definition differences. ᵍ No statistically significant asymmetry was detected in a feasible Egger test; this does not exclude publication bias. ʰ Egger testing was not performed because k<10. Because the review synthesizes non-comparative single-group proportions, comparator and relative-effect columns are not applicable. AI: artificial intelligence; CI: confidence interval; GRADE: Grading of Recommendations Assessment, Development and Evaluation; JBI: Joanna Briggs Institute; k: number of studies; PI: prediction interval.

**Figure S1**. Forest plot of AI perceived as beneficial to career (domain P2) from the random-effects meta-analysis (k=16; N=9,799). Pooled proportion: 78.4% (95% CI 69.5% to 86.2%). The 95% prediction interval (45.3%-98.3%) is printed beneath the pooled estimate and represents the range in which the true proportion may be expected for a new comparable setting under the random-effects model. Complementary model statistics are I²=98.0% and τ²=0.025; P<.001. Random-effects model: between-study variance τ² with Hartung-Knapp-Sidik-Jonkman-adjusted CI.

**Figure S2**. Forest plot of support for AI curricular integration (domain P3) from the random-effects meta-analysis (k=38; N=16,308). Pooled proportion: 76.6% (95% CI 71.8% to 81.1%). The 95% prediction interval (47.8%-96.1%) is printed beneath the pooled estimate and represents the range in which the true proportion may be expected for a new comparable setting under the random-effects model. Complementary model statistics are I²=97.2% and τ²=0.021; P<.001. Random-effects model: between-study variance τ² with Hartung-Knapp-Sidik-Jonkman-adjusted CI.

**Figure S3**. Forest plot of concern about physician replacement (domain P4) from the random-effects meta-analysis (k=32; N=16,642). Pooled proportion: 39.9% (95% CI 33.6% to 46.5%). The 95% prediction interval (6.6%-80.1%) is printed beneath the pooled estimate and represents the range in which the true proportion may be expected for a new comparable setting under the random-effects model. Complementary model statistics are I²=98.8% and τ²=0.042; P<.001. Random-effects model: between-study variance τ² with Hartung-Knapp-Sidik-Jonkman-adjusted CI.

**Figure S4**. Forest plot of prior AI use (domain P6) from the random-effects meta-analysis (k=36; N=17,374). Pooled proportion: 63.2% (95% CI 53.2% to 72.6%). The 95% prediction interval (8.5%-100.0%) is printed beneath the pooled estimate and represents the range in which the true proportion may be expected for a new comparable setting under the random-effects model. Complementary model statistics are I²=99.4% and τ²=0.091; P<.001. Random-effects model: between-study variance τ² with Hartung-Knapp-Sidik-Jonkman-adjusted CI.

**Figure S5**. Forest plot of willingness to learn about or adopt AI (domain P7) from the random-effects meta-analysis (k=22; N=9,199). Pooled proportion: 71.5% (95% CI 64.8% to 77.8%). The 95% prediction interval (37.9%-95.4%) is printed beneath the pooled estimate and represents the range in which the true proportion may be expected for a new comparable setting under the random-effects model. Complementary model statistics are I²=97.7% and τ²=0.026; P<.001. Random-effects model: between-study variance τ² with Hartung-Knapp-Sidik-Jonkman-adjusted CI.

**Figure S6**. Forest plot of ethical concerns about AI (domain P8) from the random-effects meta-analysis (k=28; N=14,571). Pooled proportion: 62.8% (95% CI 53.9% to 71.3%). The 95% prediction interval (21.9%-95.0%) is printed beneath the pooled estimate and represents the range in which the true proportion may be expected for a new comparable setting under the random-effects model. Complementary model statistics are I²=98.8% and τ²=0.042; P<.001. Random-effects model: between-study variance τ² with Hartung-Knapp-Sidik-Jonkman-adjusted CI.

**Figure S7**. Forest plot of trust in AI-assisted decisions (domain P9) from the random-effects meta-analysis (k=8; N=3,007). Pooled proportion: 50.6% (95% CI 28.5% to 72.6%). The 95% prediction interval (7.3%-93.3%) is printed beneath the pooled estimate and represents the range in which the true proportion may be expected for a new comparable setting under the random-effects model. Complementary model statistics are I²=98.1% and τ²=0.041; P<.001. Random-effects model: between-study variance τ² with Hartung-Knapp-Sidik-Jonkman-adjusted CI.

**Figure S8.** Subgroup forest plot of Positive attitude toward AI (domain P1) stratified by AI type (overall k=44; N=20,806). Overall pooled proportion: 76.9% (95% CI 72.2% to 81.4%); I²=98.3%; between-group QM=10.67; P=.005. Random-effects model: between-study variance τ² with Hartung-Knapp-Sidik-Jonkman-adjusted CI.

**Figure S9**. Subgroup forest plot of Positive attitude toward AI (domain P1) stratified by JBI risk of bias (overall k=44; N=20,806). Overall pooled proportion: 76.9% (95% CI 72.2% to 81.4%); I²=98.3%; between-group QM=0.01; P=.943. Random-effects model: between-study variance τ² with Hartung-Knapp-Sidik-Jonkman-adjusted CI.

**Figure S10**. Subgroup forest plot of Positive attitude toward AI (domain P1) stratified by Publication period (overall k=44; N=20,806). Overall pooled proportion: 76.9% (95% CI 72.2% to 81.4%); I²=98.3%; between-group QM=0.00; P=.976. Random-effects model: between-study variance τ² with Hartung-Knapp-Sidik-Jonkman-adjusted CI.

**Figure S11.** Subgroup forest plot of Positive attitude toward AI (domain P1) stratified by World Bank income (overall k=44; N=20,806). Overall pooled proportion: 76.9% (95% CI 72.2% to 81.4%); I²=98.3%; between-group QM=1.46; P=.691. Random-effects model: between-study variance τ² with Hartung-Knapp-Sidik-Jonkman-adjusted CI.

**Figure S12**. Subgroup forest plot of Support for AI curricular integration (domain P3) stratified by AI type (overall k=38; N=16,308). Overall pooled proportion: 76.6% (95% CI 71.8% to 81.1%); I²=97.2%; between-group QM=2.27; P=.322. Random-effects model: between-study variance τ² with Hartung-Knapp-Sidik-Jonkman-adjusted CI.

**Figure S13**. Subgroup forest plot of Support for AI curricular integration (domain P3) stratified by JBI risk of bias (overall k=38; N=16,308). Overall pooled proportion: 76.6% (95% CI 71.8% to 81.1%); I²=97.2%; between-group QM=2.23; P=.135. Random-effects model: between-study variance τ² with Hartung-Knapp-Sidik-Jonkman-adjusted CI.

**Figure S14**. Subgroup forest plot of Support for AI curricular integration (domain P3) stratified by Publication period (overall k=38; N=16,308). Overall pooled proportion: 76.6% (95% CI 71.8% to 81.1%); I²=97.2%; between-group QM=0.70; P=.404. Random-effects model: between-study variance τ² with Hartung-Knapp-Sidik-Jonkman-adjusted CI.

**Figure S15.** Subgroup forest plot of Support for AI curricular integration (domain P3) stratified by World Bank income (overall k=38; N=16,308). Overall pooled proportion: 76.6% (95% CI 71.8% to 81.1%); I²=97.2%; between-group QM=8.24; P=.041. Random-effects model: between-study variance τ² with Hartung-Knapp-Sidik-Jonkman-adjusted CI.

**Figure S16**. Subgroup forest plot of Concern about physician replacement (domain P4) stratified by AI type (overall k=32; N=16,642). Overall pooled proportion: 39.9% (95% CI 33.6% to 46.5%); I²=98.8%; between-group QM=8.72; P=.013. Random-effects model: between-study variance τ² with Hartung-Knapp-Sidik-Jonkman-adjusted CI.

**Figure S17**. Subgroup forest plot of Concern about physician replacement (domain P4) stratified by JBI risk of bias (overall k=32; N=16,642). Overall pooled proportion: 39.9% (95% CI 33.6% to 46.5%); I²=98.8%; between-group QM=1.87; P=.171. Random-effects model: between-study variance τ² with Hartung-Knapp-Sidik-Jonkman-adjusted CI.

**Figure S18**. Subgroup forest plot of Concern about physician replacement (domain P4) stratified by Publication period (overall k=32; N=16,642). Overall pooled proportion: 39.9% (95% CI 33.6% to 46.5%); I²=98.8%; between-group QM=1.85; P=.174. Random-effects model: between-study variance τ² with Hartung-Knapp-Sidik-Jonkman-adjusted CI.

**Figure S19**. Subgroup forest plot of Concern about physician replacement (domain P4) stratified by World Bank income (overall k=32; N=16,642). Overall pooled proportion: 39.9% (95% CI 33.6% to 46.5%); I²=98.8%; between-group test not run: Fewer than 3 studies in at least 1 moderator level: Mixed income. Random-effects model: between-study variance τ² with Hartung-Knapp-Sidik-Jonkman-adjusted CI.

**Figure S20**. Subgroup forest plot of Self-reported familiarity/knowledge (domain P5) stratified by AI type (overall k=52; N=27,817). Overall pooled proportion: 63.3% (95% CI 55.9% to 70.3%); I²=99.5%; between-group QM=9.64; P=.008. Random-effects model: between-study variance τ² with Hartung-Knapp-Sidik-Jonkman-adjusted CI.

**Figure S21**. Subgroup forest plot of Self-reported familiarity/knowledge (domain P5) stratified by JBI risk of bias (overall k=52; N=27,817). Overall pooled proportion: 63.3% (95% CI 55.9% to 70.3%); I²=99.5%; between-group QM=0.02; P=.894. Random-effects model: between-study variance τ² with Hartung-Knapp-Sidik-Jonkman-adjusted CI.

**Figure S22**. Subgroup forest plot of Self-reported familiarity/knowledge (domain P5) stratified by Publication period (overall k=52; N=27,817). Overall pooled proportion: 63.3% (95% CI 55.9% to 70.3%); I²=99.5%; between-group QM=4.08; P=.043. Random-effects model: between-study variance τ² with Hartung-Knapp-Sidik-Jonkman-adjusted CI.

**Figure S23**. Subgroup forest plot of Self-reported familiarity/knowledge (domain P5) stratified by World Bank income (overall k=52; N=27,817). Overall pooled proportion: 63.3% (95% CI 55.9% to 70.3%); I²=99.5%; between-group QM=1.50; P=.682. Random-effects model: between-study variance τ² with Hartung-Knapp-Sidik-Jonkman-adjusted CI.

**Figure S24.** Subgroup forest plot of Prior AI use (domain P6) stratified by AI type (overall k=36; N=17,374). Overall pooled proportion: 63.2% (95% CI 53.2% to 72.6%); I²=99.4%; between-group QM=1.05; P=.306. Random-effects model: between-study variance τ² with Hartung-Knapp-Sidik-Jonkman-adjusted CI.

**Figure S25**. Subgroup forest plot of Prior AI use (domain P6) stratified by JBI risk of bias (overall k=36; N=17,374). Overall pooled proportion: 63.2% (95% CI 53.2% to 72.6%); I²=99.4%; between-group QM=2.70; P=.101. Random-effects model: between-study variance τ² with Hartung-Knapp-Sidik-Jonkman-adjusted CI.

**Figure S26**. Subgroup forest plot of Prior AI use (domain P6) stratified by Publication period (overall k=36; N=17,374). Overall pooled proportion: 63.2% (95% CI 53.2% to 72.6%); I²=99.4%; between-group test not run: Fewer than 3 studies in at least 1 moderator level: Pre-2023. Random-effects model: between-study variance τ² with Hartung-Knapp-Sidik-Jonkman-adjusted CI.

**Figure S27.** Subgroup forest plot of Prior AI use (domain P6) stratified by World Bank income (overall k=36; N=17,374). Overall pooled proportion: 63.2% (95% CI 53.2% to 72.6%); I²=99.4%; between-group test not run: Fewer than 3 studies in at least 1 moderator level: Mixed income. Random-effects model: between-study variance τ² with Hartung-Knapp-Sidik-Jonkman-adjusted CI.

**Figure S28**. Leave-one-out sensitivity analysis for Positive attitude toward AI (domain P1; k=44). Each row shows the pooled proportion after omitting one study. Overall estimate: 76.9%; leave-one-out range 76.1% to 77.7%; maximum absolute shift 0.8 percentage points. Random-effects model: between-study variance τ² with Hartung-Knapp-Sidik-Jonkman-adjusted CI.

**Figure S29.** Leave-one-out sensitivity analysis for AI perceived as beneficial to career (domain P2; k=16). Each row shows the pooled proportion after omitting one study. Overall estimate: 78.4%; leave-one-out range 77.2% to 81.2%; maximum absolute shift 2.8 percentage points. Random-effects model: between-study variance τ² with Hartung-Knapp-Sidik-Jonkman-adjusted CI.

**Figure S30**. Leave-one-out sensitivity analysis for Support for AI curricular integration (domain P3; k=38). Each row shows the pooled proportion after omitting one study. Overall estimate: 76.6%; leave-one-out range 75.6% to 77.5%; maximum absolute shift 1.0 percentage points. Random-effects model: between-study variance τ² with Hartung-Knapp-Sidik-Jonkman-adjusted CI.

**Figure S31**. Leave-one-out sensitivity analysis for Concern about physician replacement (domain P4; k=32). Each row shows the pooled proportion after omitting one study. Overall estimate: 39.9%; leave-one-out range 38.7% to 40.9%; maximum absolute shift 1.3 percentage points. Random-effects model: between-study variance τ² with Hartung-Knapp-Sidik-Jonkman-adjusted CI.

**Figure S32**. Leave-one-out sensitivity analysis for Self-reported familiarity/knowledge (domain P5; k=52). Each row shows the pooled proportion after omitting one study. Overall estimate: 63.3%; leave-one-out range 62.1% to 64.4%; maximum absolute shift 1.1 percentage points. Random-effects model: between-study variance τ² with Hartung-Knapp-Sidik-Jonkman-adjusted CI.

**Figure S33**. Leave-one-out sensitivity analysis for Prior AI use (domain P6; k=36). Each row shows the pooled proportion after omitting one study. Overall estimate: 63.2%; leave-one-out range 61.6% to 64.9%; maximum absolute shift 1.7 percentage points. Random-effects model: between-study variance τ² with Hartung-Knapp-Sidik-Jonkman-adjusted CI.

**Figure S34**. Leave-one-out sensitivity analysis for Willingness to learn/adopt AI (domain P7; k=22). Each row shows the pooled proportion after omitting one study. Overall estimate: 71.5%; leave-one-out range 70.2% to 72.9%; maximum absolute shift 1.3 percentage points. Random-effects model: between-study variance τ² with Hartung-Knapp-Sidik-Jonkman-adjusted CI.

**Figure S35**. Leave-one-out sensitivity analysis for Ethical concerns about AI (domain P8; k=28). Each row shows the pooled proportion after omitting one study. Overall estimate: 62.8%; leave-one-out range 61.2% to 65.6%; maximum absolute shift 2.8 percentage points. Random-effects model: between-study variance τ² with Hartung-Knapp-Sidik-Jonkman-adjusted CI.

**Figure S36**. Leave-one-out sensitivity analysis for Trust in AI-assisted decisions (domain P9; k=8). Each row shows the pooled proportion after omitting one study. Overall estimate: 50.6%; leave-one-out range 44.1% to 57.3%; maximum absolute shift 6.7 percentage points. Random-effects model: between-study variance τ² with Hartung-Knapp-Sidik-Jonkman-adjusted CI.

**Figure S37.** Contour-enhanced funnel plot for Positive attitude toward AI (domain P1; k=44) on the Freeman-Tukey transformed proportion scale. Egger test P<.001.

**Figure S38**. Contour-enhanced funnel plot for AI perceived as beneficial to career (domain P2; k=16) on the Freeman-Tukey transformed proportion scale. Egger test P=.129.

**Figure S39**. Contour-enhanced funnel plot for Support for AI curricular integration (domain P3; k=38) on the Freeman-Tukey transformed proportion scale. Egger test P<.001.

**Figure S40**. Contour-enhanced funnel plot for Concern about physician replacement (domain P4; k=32) on the Freeman-Tukey transformed proportion scale. Egger test P=.461.

**Figure S41**. Contour-enhanced funnel plot for Self-reported familiarity/knowledge (domain P5; k=52) on the Freeman-Tukey transformed proportion scale. Egger test P<.001.

**Figure S42**. Contour-enhanced funnel plot for Prior AI use (domain P6; k=36) on the Freeman-Tukey transformed proportion scale. Egger test P<.001.

**Figure S43.** Contour-enhanced funnel plot for Willingness to learn/adopt AI (domain P7; k=22) on the Freeman-Tukey transformed proportion scale. Egger test P<.001.

**Figure S44**. Contour-enhanced funnel plot for Ethical concerns about AI (domain P8; k=28) on the Freeman-Tukey transformed proportion scale. Egger test P=.475.

**Figure S45.** Forest plot of MAIRS-MS total readiness score from the random-effects continuous meta-analysis (k=12; N=5,795). Pooled mean: 62.02 (95% CI 49.47 to 74.57); I²=100.0%; τ²=2017.434; P<.001. Random-effects model: between-study variance τ² with Hartung-Knapp-Sidik-Jonkman-adjusted CI. This secondary continuous-outcome synthesis is presented descriptively; the pooled mean is not interpreted as a transportable value or used as a basis for curricular conclusions.

**Figure S46.** Forest plot of MAIRS-MS cognition from the random-effects continuous meta-analysis (k=11; N=5,612). Pooled mean: 21.00 (95% CI 16.65 to 25.35); I²=100.0%; τ²=156.523; P<.001. Random-effects model: between-study variance τ² with Hartung-Knapp-Sidik-Jonkman-adjusted CI. This secondary continuous-outcome synthesis is presented descriptively; the pooled mean is not interpreted as a transportable value or used as a basis for curricular conclusions.

**Figure S47.** Forest plot of MAIRS-MS ability from the random-effects continuous meta-analysis (k=11; N=5,612). Pooled mean: 22.12 (95% CI 17.48 to 26.77); I²=100.0%; τ²=184.212; P<.001. Random-effects model: between-study variance τ² with Hartung-Knapp-Sidik-Jonkman-adjusted CI. This secondary continuous-outcome synthesis is presented descriptively; the pooled mean is not interpreted as a transportable value or used as a basis for curricular conclusions.

**Figure S48.** Forest plot of MAIRS-MS vision from the random-effects continuous meta-analysis (k=11; N=5,612). Pooled mean: 8.63 (95% CI 7.18 to 10.07); I²=99.9%; τ²=8.377; P<.001. Random-effects model: between-study variance τ² with Hartung-Knapp-Sidik-Jonkman-adjusted CI. This secondary continuous-outcome synthesis is presented descriptively; the pooled mean is not interpreted as a transportable value or used as a basis for curricular conclusions.

**Figure S49.** Forest plot of MAIRS-MS ethics from the random-effects continuous meta-analysis (k=11; N=5,612). Pooled mean: 9.27 (95% CI 7.64 to 10.91); I²=99.9%; τ²=10.559; P<.001. Random-effects model: between-study variance τ² with Hartung-Knapp-Sidik-Jonkman-adjusted CI. This secondary continuous-outcome synthesis is presented descriptively; the pooled mean is not interpreted as a transportable value or used as a basis for curricular conclusions.

**Figure S50.** Bubble plot for exploratory meta-regression of Positive attitude toward AI (domain P1) by AI type (k=44; P=.005). Points are sized by inverse-variance model weight; fitted meta-regression line and 95% CI band are shown. Exploratory analysis; nominal, multiplicity-unadjusted P values. The categorical AI-type moderator must not be interpreted as a continuous linear trend.

**Figure S51.** Bubble plot for exploratory meta-regression of AI perceived as beneficial to career (domain P2) by AI type (k=16; P=.040). Points are sized by inverse-variance model weight; fitted meta-regression line and 95% CI band are shown. Exploratory analysis; nominal, multiplicity-unadjusted P values. The categorical AI-type moderator must not be interpreted as a continuous linear trend.

**Figure S52.** Bubble plot for exploratory meta-regression of Concern about physician replacement (domain P4) by AI type (k=32; P=.013). Points are sized by inverse-variance model weight; fitted meta-regression line and 95% CI band are shown. Exploratory analysis; nominal, multiplicity-unadjusted P values. The categorical AI-type moderator must not be interpreted as a continuous linear trend.

**Figure S53.** Bubble plot for exploratory meta-regression of Self-reported familiarity/knowledge (domain P5) by AI type (k=52; P=.008). Points are sized by inverse-variance model weight; fitted meta-regression line and 95% CI band are shown. Exploratory analysis; nominal, multiplicity-unadjusted P values. The categorical AI-type moderator must not be interpreted as a continuous linear trend.

**Figure S54.** Bubble plot for exploratory meta-regression of self-reported familiarity/knowledge (domain P5) by publication period (2023 or later vs before 2023; k=52; P=.043). Points are sized by inverse-variance model weight; the line connects the model-estimated proportions for the two publication-period categories, and the shaded band represents the corresponding 95% CI. Exploratory analysis; nominal, multiplicity-unadjusted P value. Publication period was treated as a binary categorical moderator, and the association should not be interpreted as a continuous temporal trend or causal effect.
