# Supplementary material for: Medical Students’ Attitudes, Perceptions, and Self-Reported Familiarity With AI in Health Care: Systematic Review and Meta-Analysis
Source: JMIR Med Educ. 2026 Aug 18;12:e89411. doi: 10.2196/89411 (PMC13484950; doi:10.2196/89411)
Supplement: Supplementary file 3 [file mededu-v12-e89411-s003.docx]

PRISMA 2020 Expanded Checklist, PRISMA 2020 for Abstracts Checklist, and PRISMA-S Checklist

# PRISMA 2020 Main Checklist

| **Topic** | **No.** | **Item** | **Location where item is reported** |
| --- | --- | --- | --- |
| **TITLE** |  |  |  |
| **Title** | 1 | Identify the report as a systematic review. | Title page |
| **ABSTRACT** |  |  |  |
| **Abstract** | 2 | See the PRISMA 2020 for Abstracts checklist | See PRISMA 2020 for Abstracts checklist below |
| **INTRODUCTION** |  |  |  |
| **Rationale** | 3 | Describe the rationale for the review in the context of existing knowledge. | Introduction, paragraphs 1-4 |
| **Objectives** | 4 | Provide an explicit statement of the objective(s) or question(s) the review addresses. | Introduction, paragraph 5 |
| **METHODS** |  |  |  |
| **Eligibility criteria** | 5 | Specify the inclusion and exclusion criteria for the review and how studies were grouped for the syntheses. | Methods, Eligibility Criteria |
| **Information sources** | 6 | Specify all databases, registers, websites, organisations, reference lists and other sources searched or consulted to identify studies. Specify the date when each source was last searched or consulted. | Methods, Information Sources and Search Strategy |
| **Search strategy** | 7 | Present the full search strategies for all databases, registers and websites, including any filters and limits used. | Multimedia Appendix 1 |
| **Selection process** | 8 | Specify the methods used to decide whether a study met the inclusion criteria of the review, including how many reviewers screened each record and each report retrieved, whether they worked independently, and if applicable, details of automation tools used in the process. | Methods, Selection Process and Data Collection |
| **Data collection process** | 9 | Specify the methods used to collect data from reports, including how many reviewers collected data from each report, whether they worked independently, any processes for obtaining or confirming data from study investigators, and if applicable, details of automation tools used in the process. | Methods, Selection Process and Data Collection |
| **Data items** | 10a | List and define all outcomes for which data were sought. Specify whether all results that were compatible with each outcome domain in each study were sought (e.g. for all measures, time points, analyses), and if not, the methods used to decide which results to collect. | Methods, Data Items and Outcome Domains |
|  | 10b | List and define all other variables for which data were sought (e.g. participant and intervention characteristics, funding sources). Describe any assumptions made about any missing or unclear information. | Methods, Selection Process and Data Collection |
| **Study risk of bias assessment** | 11 | Specify the methods used to assess risk of bias in the included studies, including details of the tool(s) used, how many reviewers assessed each study and whether they worked independently, and if applicable, details of automation tools used in the process. | Methods, Risk of Bias Assessment |
| **Effect measures** | 12 | Specify for each outcome the effect measure(s) (e.g. risk ratio, mean difference) used in the synthesis or presentation of results. | Methods, Synthesis Methods |
| **Synthesis methods** | 13a | Describe the processes used to decide which studies were eligible for each synthesis (e.g. tabulating the study intervention characteristics and comparing against the planned groups for each synthesis (item 5)). | Methods, Eligibility Criteria; Methods, Data Items and Outcome Domains |
|  | 13b | Describe any methods required to prepare the data for presentation or synthesis, such as handling of missing summary statistics, or data conversions. | Methods, Synthesis Methods (Freeman-Tukey double-arcsine transformation) |
|  | 13c | Describe any methods used to tabulate or visually display results of individual studies and syntheses. | Results, Proportion Outcomes; Table 3; Figures 2-4; Multimedia Appendix 2, Figures S1-S54 |
|  | 13d | Describe any methods used to synthesize results and provide a rationale for the choice(s). If meta-analysis was performed, describe the model(s), method(s) to identify the presence and extent of statistical heterogeneity, and software package(s) used. | Methods, Synthesis Methods |
|  | 13e | Describe any methods used to explore possible causes of heterogeneity among study results (e.g. subgroup analysis, meta-regression). | Methods, Synthesis Methods (subgroup analyses and meta-regression) |
|  | 13f | Describe any sensitivity analyses conducted to assess robustness of the synthesized results. | Methods, Synthesis Methods (leave-one-out sensitivity analyses) |
| **Reporting bias assessment** | 14 | Describe any methods used to assess risk of bias due to missing results in a synthesis (arising from reporting biases). | Methods, Synthesis Methods (Egger regression test for small-study effects) |
| **Certainty assessment** | 15 | Describe any methods used to assess certainty (or confidence) in the body of evidence for an outcome. | Methods, Synthesis Methods (GRADE) |
| **RESULTS** |  |  |  |
| **Study selection** | 16a | Describe the results of the search and selection process, from the number of records identified in the search to the number of studies included in the review, ideally using a flow diagram. | Results, Study Selection; Figure 1 |
|  | 16b | Cite studies that might appear to meet the inclusion criteria, but which were excluded, and explain why they were excluded. | Results, Study Selection; Multimedia Appendix 2, Table S2 |
| **Study characteristics** | 17 | Cite each included study and present its characteristics. | Results, Study Characteristics; Table 1; Multimedia Appendix 2, Table S6 |
| **Risk of bias in studies** | 18 | Present assessments of risk of bias for each included study. | Results, Risk of Bias and Participant Independence; Table 2; Multimedia Appendix 2, Table S7 |
| **Results of individual studies** | 19 | For all outcomes, present, for each study: (a) summary statistics for each group (where appropriate) and (b) an effect estimate and its precision (e.g. confidence/credible interval), ideally using structured tables or plots. | Results, Proportion Outcomes; Multimedia Appendix 2, Table S9 |
| **Results of syntheses** | 20a | For each synthesis, briefly summarise the characteristics and risk of bias among contributing studies. | Results, Proportion Outcomes; Table 3 |
|  | 20b | Present results of all statistical syntheses conducted. If meta-analysis was done, present for each the summary estimate and its precision (e.g. confidence/credible interval) and measures of statistical heterogeneity. If comparing groups, describe the direction of the effect. | Results, Proportion Outcomes and Continuous Outcomes and Certainty of Evidence; Table 3; Figures 2-4; Multimedia Appendix 2, Table S10 and Figures S45-S49. |
|  | 20c | Present results of all investigations of possible causes of heterogeneity among study results. | Results, Subgroup Analyses and Meta-Regression; Table 4; Multimedia Appendix 2, Tables S13-S14, Figures S50-S54 |
|  | 20d | Present results of all sensitivity analyses conducted to assess the robustness of the synthesized results. | Results, Sensitivity Analyses; Table 5; Multimedia Appendix 2, Table S15 |
| **Reporting biases** | 21 | Present assessments of risk of bias due to missing results (arising from reporting biases) for each synthesis assessed. | Results, Small-Study Effects; Table 5; Multimedia Appendix 2, Table S16 |
| **Certainty of evidence** | 22 | Present assessments of certainty (or confidence) in the body of evidence for each outcome assessed. | Results, Risk of Bias and Participant Independence; Table 6; Multimedia Appendix 2, Table S17 |
| **DISCUSSION** |  |  |  |
| **Discussion** | 23a | Provide a general interpretation of the results in the context of other evidence. | Discussion, Principal Findings; Discussion, Comparison With Prior Work |
|  | 23b | Discuss any limitations of the evidence included in the review. | Discussion, Strengths and Limitations |
|  | 23c | Discuss any limitations of the review processes used. | Discussion, Strengths and Limitations |
|  | 23d | Discuss implications of the results for practice, policy, and future research. | Discussion, Implications for AI Literacy Curriculum Development |
| **OTHER INFORMATION** |  |  |  |
| **Registration and protocol** | 24a | Provide registration information for the review, including register name and registration number, or state that the review was not registered. | Methods, Study Registration and Reporting; PROSPERO CRD420251120543 |
|  | 24b | Indicate where the review protocol can be accessed, or state that a protocol was not prepared. | The protocol is accessible via the PROSPERO registry |
|  | 24c | Describe and explain any amendments to information provided at registration or in the protocol. | Deviations from the registered protocol: databases expanded from 3 to 6; outcome domains specified as 9 proportion domains and 5 pooled MAIRS-MS outcomes (the total score plus cognition, ability, vision, and ethics), with other noncomparable continuous measures summarized descriptively; GRADE certainty assessment added; participant-independence audit added. |
| **Support** | 25 | Describe sources of financial or non-financial support for the review, and the role of the funders or sponsors in the review. | Funding Statement |
| **Competing interests** | 26 | Declare any competing interests of review authors. | Conflicts of Interest |
| **Availability of data, code and other materials** | 27 | Report which of the following are publicly available and where they can be found: template data collection forms; data extracted from included studies; data used for all analyses; analytic code; any other materials used in the review. | Data Availability; Multimedia Appendix 2 |

*From:* Page MJ, McKenzie JE, Bossuyt PM, Boutron I, Hoffmann TC, Mulrow CD, et al. The PRISMA 2020 statement: an updated guideline for reporting systematic reviews. MetaArXiv. 2020, September 14. DOI: 10.31222/osf.io/v7gm2. For more information, visit: <www.prisma-statement.org>

# PRISMA 2020 for Abstracts Checklist

| **Section and Topic** | **Item #** | **Checklist item** | **Reported (Yes/No)** |
| --- | --- | --- | --- |
| **TITLE** | | |  |
| Title | 1 | Identify the report as a systematic review. | Yes — Title page |
| **BACKGROUND** | | |  |
| Objectives | 2 | Provide an explicit statement of the main objective(s) or question(s) the review addresses. | Yes — Abstract, Objective |
| **METHODS** | | |  |
| Eligibility criteria | 3 | Specify the inclusion and exclusion criteria for the review. | Yes — Abstract, Methods |
| Information sources | 4 | Specify the information sources (e.g. databases, registers) used to identify studies and the date when each was last searched. | Yes — Abstract, Methods (6 databases, inception to April 1, 2026) |
| Risk of bias | 5 | Specify the methods used to assess risk of bias in the included studies. | Yes — Abstract, Methods (JBI checklist) |
| Synthesis of results | 6 | Specify the methods used to present and synthesise results. | Yes — Abstract, Methods (Freeman-Tukey, HKSJ, prediction intervals, GRADE) |
| **RESULTS** | | |  |
| Included studies | 7 | Give the total number of included studies and participants and summarise relevant characteristics of studies. | Yes — Abstract, Results (96 studies, >45,000 medical students, 37 countries) |
| Synthesis of results | 8 | Present results for main outcomes, preferably indicating the number of included studies and participants for each. If meta-analysis was done, report the summary estimate and confidence/credible interval. If comparing groups, indicate the direction of the effect (i.e. which group is favoured). | Yes — Abstract, Results (8 principal domain estimates with CIs and outcome-specific study and participant counts) |
| **DISCUSSION** | | |  |
| Limitations of evidence | 9 | Provide a brief summary of the limitations of the evidence included in the review (e.g. study risk of bias, inconsistency and imprecision). | Yes — Abstract, Conclusions (wide prediction intervals, very low GRADE certainty) |
| Interpretation | 10 | Provide a general interpretation of the results and important implications. | Yes — Abstract, Conclusions |
| **OTHER** | | |  |
| Funding | 11 | Specify the primary source of funding for the review. | No — funding is reported in the Funding Statement rather than in the abstract |
| Registration | 12 | Provide the register name and registration number. | Yes — Abstract, Methods (PROSPERO CRD420251120543) |

*From:*  Page MJ, McKenzie JE, Bossuyt PM, Boutron I, Hoffmann TC, Mulrow CD, et al. The PRISMA 2020 statement: an updated guideline for reporting systematic reviews. BMJ 2021;372:n71. doi: 10.1136/bmj.n71. This work is licensed under CC BY 4.0. To view a copy of this license, visit <https://creativecommons.org/licenses/by/4.0/>

**PRISMA-S Checklist**

| **Section/topic** | **#** | **Checklist item** | **Location(s) Reported** |
| --- | --- | --- | --- |
| **INFORMATION SOURCES AND METHODS** | | | |
| Database name | 1 | Name each individual database searched, stating the platform for each. | Methods, Information Sources (PubMed/MEDLINE, Embase via Embase.com, Web of Science via Clarivate, Scopus via Elsevier, PsycINFO via Ovid, Cochrane CENTRAL via Cochrane Library) |
| Multi-database searching | 2 | If databases were searched simultaneously on a single platform, state the name of the platform, listing all of the databases searched. | Each database was searched individually on its native platform; no multi-database platform was used |
| Study registries | 3 | List any study registries searched. | PROSPERO was searched for prior systematic reviews; trial registries were not searched as only cross-sectional surveys were eligible |
| Online resources and browsing | 4 | Describe any online or print source purposefully searched or browsed (e.g., tables of contents, print conference proceedings, web sites), and how this was done. | Methods, Information Sources (Google Scholar first 200 results screened) |
| Citation searching | 5 | Indicate whether cited references or citing references were examined, and describe any methods used for locating cited/citing references (e.g., browsing reference lists, using a citation index, setting up email alerts for references citing included studies). | Methods, Information Sources (backward citation tracking of all included studies and 3 prior reviews; forward citation tracking via Scopus) |
| Contacts | 6 | Indicate whether additional studies or data were sought by contacting authors, experts, manufacturers, or others. | Authors/experts were not contacted for additional data; this is noted as a limitation in the Discussion |
| Other methods | 7 | Describe any additional information sources or search methods used. | No additional methods beyond those described |
| **SEARCH STRATEGIES** | | | |
| Full search strategies | 8 | Include the search strategies for each database and information source, copied and pasted exactly as run. | Multimedia Appendix 1 (full strategies for all 6 databases, copied exactly as executed) |
| Limits and restrictions | 9 | Specify that no limits were used, or describe any limits or restrictions applied to a search (e.g., date or time period, language, study design) and provide justification for their use. | Methods, Eligibility Criteria (no restrictions by language, publication date, or geography) |
| Search filters | 10 | Indicate whether published search filters were used (as originally designed or modified), and if so, cite the filter(s) used. | No published search filters were used; the search strategy was developed de novo |
| Prior work | 11 | Indicate when search strategies from other literature reviews were adapted or reused for a substantive part or all of the search, citing the previous review(s). | Methods, Information Sources (search strategies informed by 3 prior systematic reviews [12-14]) |
| Updates | 12 | Report the methods used to update the search(es) (e.g., rerunning searches, email alerts). | Methods, Information Sources (search last updated April 1, 2026) |
| Dates of searches | 13 | For each search strategy, provide the date when the last search occurred. | Multimedia Appendix 1 (all searches: inception to April 1, 2026) |
| **PEER REVIEW** | | | |
| Peer review | 14 | Describe any search peer review process. | A formal PRESS peer-review process was not conducted; the search strategy was developed by an information-trained reviewer and verified internally by a second reviewer |
| **MANAGING RECORDS** | | | |
| Total Records | 15 | Document the total number of records identified from each database and other information sources. | Results, Study Selection; Figure 1 (PubMed 2387; Embase 4132; WoS 1617; Scopus 4494; PsycINFO 204; CENTRAL 97; Other 77; Total 13,008) |
| Deduplication | 16 | Describe the processes and any software used to deduplicate records from multiple database searches and other information sources. | Methods, Selection Process (EndNote 21; automated matching by DOI, title, author, year, journal; manual verification; 6649 duplicates removed; 6359 unique); Multimedia Appendix 2, Table S1 |
|  |  |  |  |
| PRISMA-S: An Extension to the PRISMA Statement for Reporting Literature Searches in Systematic Reviews | | |  |
| Rethlefsen ML, Kirtley S, Waffenschmidt S, Ayala AP, Moher D, Page MJ, Koffel JB, PRISMA-S Group. | | |  |
| Last updated February 27, 2020. | |  |  |
